# Maternal and umbilical cord serum lipids in gestational diabetes predict offspring insulin secretion and resistance at the age of nine years

**DOI:** 10.1007/s11306-025-02281-9

**Published:** 2025-06-22

**Authors:** Mikael Huhtala, Tapani Rönnemaa, Kristiina Tertti, Harri Niinikoski, Elisa Paavilainen

**Affiliations:** 1 Department of Obstetrics and Gynecology, Turku University Hospital, University of Turku, Kiinamyllynkatu 4-8, Turku, FI- 20014 Finland; 2 Department of Obstetrics and Gynecology, Turku University Hospital, Kiinamyllynkatu 4-8, Turku, FI-20521 Finland; 3 Department of Medicine, University of Turku, Turku, FI-20014 Finland; 4 Division of Medicine, Turku University Hospital, Kiinamyllynkatu 4-8, Turku, FI-20521 Finland; 5 Department of Pediatrics and Adolescent Medicine, University of Turku and University Hospital of Turku, Turku, Finland

**Keywords:** Gestational diabetes, Insulin resistance, Insulin secretion, Metformin, Metabolomics, Lipidomics

## Abstract

**Introduction:**

Maternal metabolism in pregnancy is a major determinant of intrauterine milieu and is assumed to have long-term consequences in the offspring.

**Objectives:**

To study whether maternal or cord serum lipids are related to measures of insulin resistance and β-cell function in childhood.

**Methods:**

This is a secondary analysis of a previous trial in which women with newly diagnosed gestational diabetes were randomized to metformin versus insulin treatment. Maternal serum lipids were measured during pregnancy and umbilical cord serum lipids at delivery. Offspring insulin resistance and β-cell function were assessed at nine years of age using serum insulin, C-peptide, and glucose concentrations measured during an oral glucose tolerance test. A total of 122 mother-child dyads were included in the analyses.

**Results:**

After adjusting for multiple comparisons, higher cord serum docosahexaenoic acid, linoleic acid, and the ratio of linoleic acid to total fatty acids were significantly related to lower indices of β-cell function in childhood. In interaction models, cord serum linoleic acid was inversely related to offspring HOMA2-IR and measures of β-cell function only in the participants treated with insulin in pregnancy. Associations between maternal lipids and outcomes were not significant after Bonferroni adjustment.

**Conclusion:**

Cord serum lipids, and potentially maternal lipids, are related to childhood insulin function. These findings highlight the importance of maternal lipid metabolism in pregnancies affected by gestational diabetes. Given the observed differences between metformin and insulin treatment groups, the feto-placental effects of prenatal metformin exposure should be further investigated.

**Trial registration number:**

NCT02417090 at ClinicalTrials.gov, registered April 14th 2015.

**Trial registration:**

This is secondary analysis of a previous study registered at ClinicalTrialg.gov (NCT02417090) on April 14th 2015.

**Supplementary Information:**

The online version contains supplementary material available at 10.1007/s11306-025-02281-9.

## Introduction

According to the developmental origins of health and diseases hypothesis, early-life exposures can have long-term effects on individual’s health (Gillman, 2005). Gestational diabetes (GDM) and maternal obesity are important early stressors that place a burden on the fetus and influence the long-term health of the offspring (Catalano et al., 2009; Lowe et al., 2019).

Maternal hyperglycemia, as seen in GDM, leads to fetal hyperglycemia and hyperinsulinemia (Pedersen et al., 1954). However, beyond hyperglycemia, GDM is associated with broader metabolic dysregulation (White et al., 2017), which also affects fetal metabolism (Freinkel, 1980). Both maternal GDM and obesity have been linked to increased insulin resistance and insulin secretion in offspring (Sauder et al., 2017). While the exact mediators of these effects remain unclear, maternal metabolites other than glucose are likely involved.

To date, only one prior study has evaluated the long-term effects of maternal and cord serum metabolomes on offspring health. In the HAPO follow-up study, maternal metabolite profiles during pregnancy were associated with offspring body composition at 10–14 years of age (Bianco et al., 2023). However, insulin resistance and β-cell function were not assessed.

The aim of the present study was to further investigate whether maternal and umbilical cord serum lipid metabolomes (i.e. lipidomes) are related to insulin secretion and insulin resistance in offspring, using a prospective cohort of mother-child dyads consisting of mothers with GDM and their 9-year-old children.

Metformin and insulin, two commonly used antihyperglycemic agents in GDM, have partially divergent effects on maternal lipid profiles in pregnancy (Huhtala et al., 2020). Although controversy exists regarding long-term effects of intrauterine metformin exposure (Fu et al. 2025), we tested for potential interaction effects by metformin vs. insulin treatment, as well as offspring sex.

## Methods

### Study population

This is a secondary analysis of a previous prospective follow-up of a randomized trial comparing metformin and insulin treatments of GDM at Turku University Hospital (Finland). The study protocol and primary outcomes have been reported previously (Paavilainen et al., 2021; Tertti et al., 2013). Briefly, women with newly diagnosed GDM between June 2006 and December 2010 and requiring pharmacological therapy were randomized to receive either insulin or metformin. GDM was diagnosed based on at least two abnormal values in a 2-hour oral glucose tolerance test (OGTT). During the recruitment period, the Finnish national guidelines for GDM diagnosis were revised. Before December 2008, the plasma glucose cut-off values were ≥ 4.8 (fasting), ≥ 10.0 (1 h), and ≥ 8.7 mmol/L (2 h). After the revision, the thresholds were ≥ 5.3 (fasting), ≥ 10.0 (1 h), and ≥ 8.6 mmol/L (2 h). The primary outcome of the original trial, birth weight, did not differ significantly between the treatment groups.

The offspring were evaluated between August 2015 and November 2019 at approximately nine years of age. Of the 217 pregnancies that completed the original trial, 127 children (58.5%) were successfully recruited for the follow-up study (Paavilainen et al., 2021). One child was diagnosed with type 1 diabetes and was excluded from the study. A comparison of anthropometric measures and glucose metabolism between the offspring has been previously published (Paavilainen et al., 2021).

Gestational weight gain (GWG) was assessed as early (from the first antenatal visit to the initiation of pharmacological treatment for GDM) and total GWG. Preterm birth was defined as delivery before 37 completed gestational weeks. Birth weight was measured in grams and adjusted for neonatal sex and gestational age according to population-based standards (Z-score) (Sankilampi et al., 2013). Large for gestational age (LGA) and small for gestational age (SGA) were defined as adjusted birth weight above the 90th or below the 10th percentile, respectively. Offspring weight and height were measured, and the age- and sex-adjusted body mass index (ISOBMI) was calculated using the method described by Saari et al. (2011). ISOBMI reflects the projected adult BMI if the child’s weight status remains consistent relative to peers. It was used instead of plain BMI to allow comparison across the entire cohort regardless of sex.

The study participants provided an informed consent. Both the original randomized trial and the follow-up study were approved by the Ethics Committee of the Hospital District of Southwest Finland. The studies were registered at ClinicalTrials.gov (NCT01240785, NCT02417090).

### Predictors

Maternal fasting serum samples were collected during pregnancy at the initiation of pharmacological treatment for GDM (baseline; mean ± SD: 30.4 ± 1.8 gestational weeks) and again at 36 gestational weeks (h36). Umbilical cord mixed blood serum samples were collected at birth. All samples were stored at temperatures below − 70 °C. A targeted serum lipidomic analysis was subsequently performed using a high-throughput ^1^H nuclear magnetic resonance (NMR) spectroscopy protocol (Soininen et al., 2009). The analysis included detailed lipoprotein profiles, apolipoproteins, fatty acids (FA), and phospholipids, totaling 122 metabolites.

In cord serum samples, lipid concentrations in medium-sized to extremely large (M–XXL) very low-density lipoprotein (VLDL) subclasses were undetectably low in more than 20% of the samples and were therefore excluded from the analyses.

### Outcome measures

Insulin and glucose metabolism in the 9-year-old offspring were evaluated by 2-h OGTT with measurement of glucose, insulin, and C-peptide at 0, 30, and 120 min (Paavilainen et al., 2021). For those children weighing less than 43 kg the glucose load was 1.75 g/kg, and otherwise 75 g. Insulin resistance was estimated using homeostasis model assessment of insulin resistance 2 (HOMA2-IR) (Levy et al., 1998). β-cell function was estimated using ratio of the total area-under-the-insulin-curve to the total area-under-the-glucose-curve (AUC_Ins/Gluc_) calculated from the OGTT curves using the trapezoidal rule (Retnakaran et al., 2008). C-peptide based measures of insulin resistance and insulin secretion have both better repeatability and discriminatory potential (Bacha et al., 2008; Hudak et al., 2021; Utzschneider et al., 2007) compared to insulin based measures and were therefore also deployed in this study (HOMA2-IR_CP_ and AUC_CP/Gluc_). Finally, β-cell function in relation to insulin resistance was estimated by oral disposition index (oDI_Ins_ and oDI_CP_) defined as 1/Insulin_0_ × ΔInsulin_0–30_/ΔGlucose_0–30_ and 1/C-peptide_0_ × ΔC-peptide_0–30_/ΔGlucose_0–30_, respectively (Sjaarda et al., 2012; Utzschneider et al., 2009).

### Statistical analysis

The descriptive data were summarized using proportions, means ± SD, or medians with interquartile ranges, as appropriate. Clinical characteristics were compared between those included vs. not included in the current analyses, by using t-test, Chi-square test or Fisher’s exacttest. Univariate regression models were used to study the associations between clinically relevant perinatal variables and offspring insulin and glucose metabolism, and to describe the associations between offspring characteristics at follow-up and outcome variables. Associations between serum metabolites and insulin metabolism outcome measures were estimated using linear regression models. Regression models were additionally adjusted for confounding factors. The following variables were considered as confounders: maternal age, primiparity, pre-pregnancy BMI (pBMI), GWG, and smoking during pregnancy. Confounders were included in the adjusted models based on their associations with outcome variables in univariate analyses. Additionally, an exploratory analysis of univariate associations between serum metabolites and offspring ISOBMI was conducted. Interaction effects by original treatment allocation (metformin vs. insulin) and offspring sex (male vs. female) were tested using linear regression models.

Prior to conducting regression analyses, all continuous variables were centered and scaled. Outcome variables and cord serum lipoprotein lipid concentrations exhibited skewed distributions and were therefore log-transformed. Before applying log-transformation, zero values were substituted with 0.5 times the minimum non-zero value to enable transformation. When included as a covariate, maternal pBMI was categorized into three groups: normal weight (18.5–24.9 kg/m^2^), overweight (25–29.9 kg/m^2^), and obese (≥ 30 kg/m^2^).

To select confounders and to compare participants with non-participants, we used a p-value threshold of 0.05. In the main analyses, due to multiple testing and the presence of intercorrelated predictors, we applied a lower threshold of *p* < 0.01. Additionally, principal component analysis of the pooled metabolomic data revealed that 13 components explained 95% of the total variance. Based on this, we applied a Bonferroni-adjusted significance threshold of *p* < 0.0038 (0.05 / 13) to further control for type I error.

Missing predictor data were handled by case-wise exclusion. All analyses were performed using R statistical software (version 4.3.2). Figures were created using the *ggplot2* package (Wickham, 2016) for R, and the flowchart was drawn using Apple Pages (version 12.2.1).

## Results

**Fig. 1:**
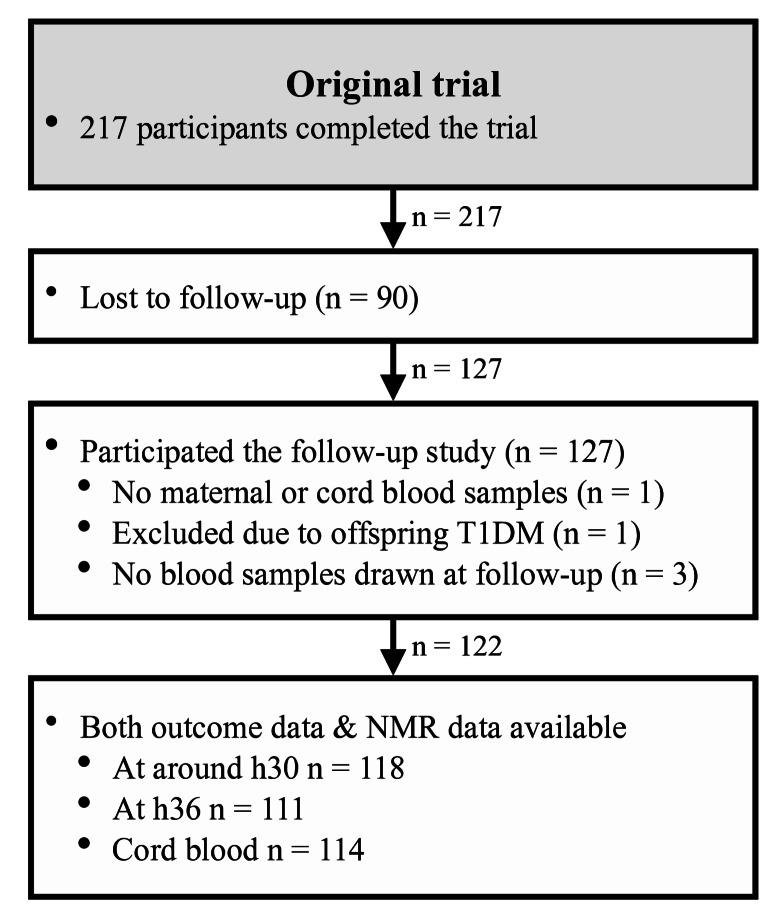
Study flowchart. T1DM: type 1 diabetes, NMR: nuclear magnetic resonance (spectroscopy)

Of the 127 children included in the follow-up study at Turku University Hospital (Paavilainen et al., 2021), 122 had OGTT data and at least one available maternal or umbilical cord serum sample (Fig. 1). Among these, metabolomic data were available for 118 participants at baseline (approximately h30), 111 at h36, and 114 at delivery (cord blood). Clinical characteristics of the study population (*n* = 122) are reported in Table 1. Compared to those not included in this study, the 122 participants were older during pregnancy (mean age 32.6 vs. 31.2 years, *p* = 0.043). No other significant differences were observed (Supplementary Table 1).

**Table 1:** Population characteristics

	Total		Metformin		Insulin	
	n	*Mean ± SD*,* median (IQR)*,* or n (%)*	n	*Mean ± SD*,* median (IQR)*,* or n (%)*	n	*Mean ± SD*,* median (IQR)*,* or n (%)*
*Pregnancy characteristics*						
Age (years)	122	32.6 ± 5.0	59	32.9 ± 4.8	63	32.4 ± 5.3
BMI (kg/m^2^)	122	29.0 ± 5.4	59	29.5 ± 6.0	63	28.6 ± 4.8
BMI-class	122		59		63	
Normal weight (BMI < 25 kg/m^2^)		22 (18%)		11 (19%)		11 (17%)
Overweight (BMI 25–29.9 kg/m^2^)		52 (43%)		22 (37%)		30 (48%)
Obese (BMI ≥ 30 kg/m^2^)		48 (39%)		26 (44%)		22 (35%)
Smoking (n)	120	14 (12%)	58	4 (6.9%)	62	10 (16%)
Primiparous (n)	122	47 (39%)	59	18 (31%)	63	29 (46%)
Early weight gain in pregnancy (kg)	122	5.8 ± 3.8	59	6.0 ± 4.0	63	5.6 ± 3.7
Weight gain in pregnancy (kg)	122	8.0 ± 4.8	59	8.0 ± 4.9	63	8.0 ± 4.8
Gestational age at OGTT (weeks)	122	26.8 ± 2.4	59	26.5 ± 3.0	63	27.0 ± 1.7
OGTT fasting glucose (mmol/L)	122	5.5 ± 0.5	59	5.5 ± 0.6	63	5.5 ± 0.4
OGTT 1 h glucose (mmol/L)	122	11.2 ± 1.3	59	11.3 ± 1.4	63	11.1 ± 1.1
OGTT 2 h glucose (mmol/L)	120	8.1 ± 1.8	58	8.3 ± 1.9	62	8.0 ± 1.7
HbA1c at baseline (%)	122	5.46 ± 0.34	59	5.43 ± 0.36	63	5.49 ± 0.32
HbA1c at baseline (mmol/mol)	122	36.1 ± 3.7	59	35.8 ± 4.0	63	36.4 ± 3.5
Gestational age at delivery (weeks)	122	39.3 ± 1.6	59	39.2 ± 1.5	63	39.4 ± 1.7
Preterm birth (n)	122	7 (5.7%)	59	5 (8.5%)	63	2 (3.2%)
Cesarean delivery (n)	122	23 (19%)	59	9 (15%)	63	14 (22%)
Birth weight (g)	122	3,590 ± 490	59	3,630 ± 500	63	3,550 ± 480
Birth weight (Z-score)	122	0.07 ± 1.11	59	0.19 ± 1.11	63	-0.04 ± 1.10
SGA (n)	122	14 (11%)	59	5 (8.5%)	63	9 (14%)
LGA (n)	122	18 (15%)	59	9 (15%)	63	9 (14%)
*Offspring at follow-up*						
Sex	122		59		63	
Male		59 (48%)		30 (51%)		29 (46%)
Female		63 (52%)		29 (49%)		34 (54%)
Age (years)	122	9.1 ± 0.1	59	9.1 ± 0.1	63	9.0 ± 0.1
Height (cm)	122	137 ± 6	59	137 ± 6	63	137 ± 6
Weight (kg)	122	34 ± 7	59	34 ± 6	63	35 ± 7
ISOBMI (kg/m^2^)	122	23.5 ± 3.6	59	23.2 ± 3.5	63	23.8 ± 3.7
OGTT fasting glucose (mmol/L)	122	5.0 ± 0.4	59	5.0 ± 0.4	63	5.1 ± 0.4
OGTT 30 min glucose (mmol/L)	121	8.4 ± 1.6	59	8.4 ± 1.4	62	8.4 ± 1.7
OGTT 120 min glucose (mmol/L)	121	5.5 ± 1.0	59	5.3 ± 1.0	62	5.6 ± 1.0
Fasting insulin (mU/L)	122	8.1 (5.3, 12.0)	59	7.7 (5.1, 11.8)	63	8.5 (5.7, 12.5)
Fasting C-peptide (nmol/L)	122	0.40 (0.32, 0.53)	59	0.39 (0.33, 0.49)	63	0.41 (0.32, 0.54)

GDM: gestational diabetes, ISOBMI: BMI adjusted for age and sex, IQR: interquartile range, LGA: large for gestational age, OGTT: oral glucose tolerance test, SD: standard deviation, SGA: small for gestational age

The outcome measured of the children are presented in Table 2. In two participants, a decreasing or stable glucose during the first 30 min of the OGTT resulted in a negative or undefined oDI; these values were discarded. As expected, hyperbolic relationships were found between insulin sensitivity and measures of β-cell function (Supplementary Fig. 1).

**Table 2:** Outcome measures in the children at the age of nine years

	*n*	Median (IQR)
*Insulin secretion / insulin resistance*		
oDI_Ins_ (mM^− 1^)	119	2.05 (1.39, 3.00)
oDI_CP_ (mM^− 1^)	119	1.00 (0.72, 1.41)
*Insulin resistance*		
HOMA2-IR_Ins_	122	1.05 (0.69, 1.55)
HOMA2-IR_CP_	122	0.89 (0.69, 1.16)
*Insulin secretion*		
AUC_Ins/Gluc_ (mU/mol)	120	6.36 (4.61, 8.11)
AUC_CP/Gluc_ (nmol/mol)	120	0.22 (0.17, 0.27)

AUC: area under curve, HOMA2-IR: homeostasis model assessment of insulin resistance 2, oDI: oral disposition index, IQR: interquartile range

High pBMI (≥ 30 kg/m^2^) was significantly associated with HOMA2-IR_Ins_, AUC_Ins/Gluc_, and AUC_CP/Gluc_ (Supplementary Table 2). It was the confounder with most and strongest associations with outcome variables and was therefore selected as a covariate in the adjusted models.

### Associations between maternal and umbilical cord serum metabolites and outcome variables

**Fig. 2:**
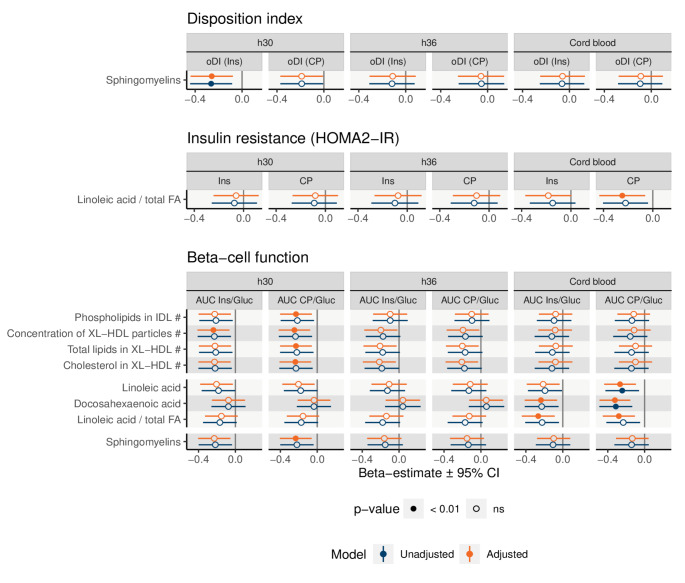
Associations between metabolites and outcome variables. Only those metabolites are included which have at least one significant (*p* < 0.01) association. Adjusted model is adjusted for pBMI. In the cord serum lipoprotein lipids were log-converted (#). ns: non-significant (*p*-value ≥ 0.01). HDL: high-density lipoprotein, IDL: intermediate-density lipoprotein, FA: fatty acids

Associations in unadjusted and pBMI adjusted associations are listed in Supplementary Tables 3–5. Significant associations (*p* < 0.01) between metabolites and outcome variables are summarized in Fig. 2. Maternal sphingomyelins at h30 were inversely related to oDI_Ins_ independent of adjustments. Additionally, a significant inverse association between sphingomyelins at h30 and AUC_CP/Gluc_ was found in the adjusted model.

Proportion of linoleic acid (LA) in umbilical cord was inversely related to HOMA2-IR_CP_ and AUC_CP/Gluc_ with statistically significant associations in the adjusted model. Also in cord serum, docosahexaenoic acid (DHA) and LA were inversely associated with β-cell function.

The association between intermediate-density lipoprotein (IDL) phospholipids, XL high-density lipoprotein (HDL) total lipids and XL-HDL cholesterol, and AUC_CP/Gluc_ were statistically significant only at h30 in the adjusted model. XL-HDL particle concentration at h30 was inversely related to both measures of β-cell function in the adjusted models.

When Bonferroni-corrected threshold was applied, only the associations between cord serum DHA, LA, LA-to-total-FA-ratio and AUC_CP/Gluc_ remained significant in the adjusted model (Supplementary Table 5). The association between cord serum LA and AUC_CP/Gluc_ was significant also in the unadjusted model.

None of the metabolites were significantly related to ISOBMI (Supplementary Table 6).

### Effect of GDM treatment

**Fig. 3:**
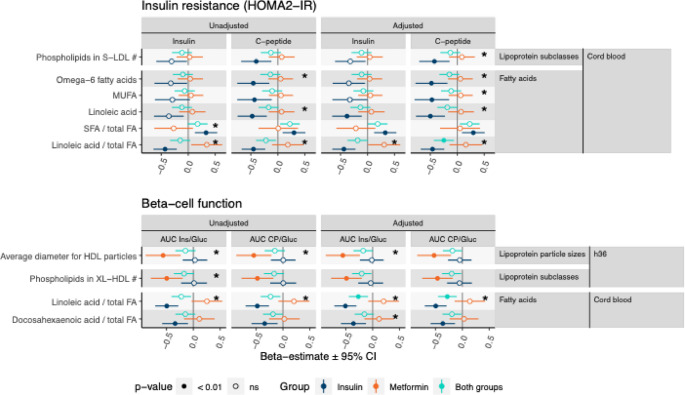
The effects of GDM treatment in pregnancy on associations between metabolites and outcome variables. Only those metabolites are included which have at least one significant (*p* < 0.01) association in any subgroup and significant interaction (interaction term p*p* 0.01) in at least one of the associations. Adjusted model. Significant interaction terms are noted with an asterisk (*). Cord serum lipoprotein lipids were log-transformed (#). ns: non-significant (*p*-value ≥ 0.01). HDL: high-density lipoprotein, LDL: low-density lipoprotein, FA: fatty acids, MUFA: monounsaturated FA, SFA: saturated FA

The effect of GDM treatment on associations was tested by adjusting for treatment interaction (predictor × treatment). Only associations with significant interaction (*p* < 0.01) are considered (Fig. 3). In cord serum the associations between FA classes and offspring insulin resistance were significant mostly in the insulin group. Cord serum LA-to-total-FA-ratio was inversely related to both HOMA2-IR and AUC_Ins/Gluc_ and AUC_CP/Gluc_ in the insulin group. Absolute cord serum LA was also inversely related to both HOMA2-IR measures in the insulin group, with significant interaction in the adjusted models. Monounsaturated FA (MUFA) and omega-6 FA were inversely related to HOMA2-IR_CP_ in the insulin treated pregnancies. Additionally, in the insulin group, the saturated-FA-to-total-FA-ratio in cord serum was positively related to HOMA2-IR_Ins_, but the interaction was significant only in the unadjusted model. Cord serum DHA-to-total-FA-ratio was inversely related to AUC_Ins/Gluc_ in the insulin group with significant interaction in the adjusted model.

At h36, HDL particle size was inversely related to AUC_Ins/Gluc_ and AUC_CP/Gluc_ in the metformin group while the association was not significant in the insulin group. Accordingly, maternal phospholipids in XL-HDL at h36 were inversely related to β-cell function in the metformin group. Interaction term regarding XL-HDL phospholipids and AUC_Ins/Gluc_ was only in the unadjusted model.

Small low-density lipoprotein (S-LDL) phospholipids in cord serum were inversely related to HOMA2-IR_CP_ in the insulin group. The interaction was significant only after adjusting for pBMI.

Using the stricter threshold of *p* < 0.0038, associations between the cord serum LA-to-total-FA-ratio, HOMA2-IR and markers of β-cell function were statistically significant in the insulin treatment group (Supplementary Table 7). Additionally, the association between cord serum LA and HOMA2-IR_CP_ remained statistically under the same threshold. These associations showed significant interaction effects with the treatment group (*p* < 0.0038).

### Effect of offspring sex

The effect of offspring sex on associations was evaluated using interaction term (predictor × offspring sex) in regression models. None of the associations met the criteria for significant interaction (*p* < 0.01) combined with a significant association in either group (*p* < 0.01).

## Discussion

In this prospective study, we found several maternal lipids to be associated with insulin–glucose metabolism in the offspring. This finding aligns with the hypothesis proposed by Dr. Freinkel over four decades ago (Freinkel 1980). Cord serum lipid concentrations were associated with insulin resistance and β-cell function in the offspring, and these associations were modulated by maternal treatment during pregnancy (i.e. metformin vs. insulin). Metformin passes across the placenta (Tertti et al., 2010; Vanky et al., 2005) and affects fetal metabolism (Estrella et al., 2021; Huhtala et al., 2021), although the exact mechanisms remain unclear. The observation that cord serum metabolite associations differed by treatment group supports a potential role for *in utero* metformin exposure in shaping offspring metabolic outcomes.

Overall, most of the observed associations were related to β-cell function rather than insulin resistance or oDI. The offspring, at nine years of age, were generally healthy, and none of those included in the analyses had diabetes. Therefore, it is assumed that the participants were in a compensated metabolic state, in which β-cell function was sufficient to maintain normal glucose levels within varying levels of insulin resistance. This likely explains the relatively small variation observed in oDI-values, and hence lower statistical power to detect associations.

In our data, more adverse metabolite profile– characterized by lower levels of LA, DHA, and XL-HDL– was associated with increased markers of β-cell function. The seemingly elevated β-cell function in this non-diabetic population likely reflects an early disturbance in insulin metabolism. This interpretation is supported by findings from a large prospective study, which demonstrated that increased β-cell function, as estimated by the HOMA2 model, precedes the onset of type 2 diabetes by more than a decade, followed by a steep decline in function shortly before the development of hyperglycemia (Tabák et al., 2009). Similarly, exposure to maternal diabetes has been shown to associate with increased β-cell function and insulin resistance in offspring, yet no associations with oDI were observed (Sauder et al., 2017).

We deployed both insulin and C-peptide based estimates of insulin resistance, β-cell function, and oDI in our study. As expected, the associations were largely similar between equivalent insulin and C-peptide estimates, although minor differences were also noted. It has previously been demonstrated that C-peptide compared to insulin based estimates are more reproducible (Utzschneider et al., 2007), and due to shorter half-life of insulin, C-peptide provides more reliable estimate for the insulin secretion from pancreatic β-cells. On the contrary, insulin clearance affects insulin concentration more than C-peptide clearance affects its concentration, leading to less variation in C-peptide but losing some details about insulin’s biological effects.

Although higher offspring ISOBMI was related to higher HOMA2-IR, AUC_Ins/Gluc_, and AUC_CP/Gluc_ we did not find significant associations between the metabolites and ISOBMI. Possibly, there are other, lifestyle and diet related confounders, that prevent us observing these associations. The associations between prenatal lipid exposure and childhood adiposity remain plausible however (Bianco et al., 2023), and could even be mediated by impaired insulin resistance.

In cord serum, mostly FA were related to insulin resistance and markers of β-cell function in the offspring. In the whole population polyunsaturated FA (PUFA) LA and DHA were related to lower AUC_CP/Gluc_ independent of maternal pBMI. Interestingly, the proportions of LA and DHA in cord serum were related to β-cell function estimates only in the insulin group, when evaluated separately. Similar pattern was observed regarding the inverse associations between cord serum omega-6 FA, total MUFA, LA and HOMA2-IR_CP_ that were significant only in the insulin group.

Inverse associations between cord serum DHA and LA with AUC_CP/Gluc_, and between cord serum LA and HOMA2-IR in the insulin group, were the only associations that remained statistically significant after applying the Bonferroni-adjusted threshold (*p* < 0.0038). While this result provides an additional level of confidence in these findings, we believe that the associations should be interpreted within a broader context. The limited sample size decreased our statistical power, and the long follow-up interval (nine years) between exposure and outcome likely introduced additional heterogeneity. Nevertheless, the observed relationship between cord serum FA profile and childhood β-cell capacity seems likely. Other findings, although biologically plausible, should be interpreted more cautiously.

Few previous studies have characterized the associations between cord serum PUFA and offspring adiposity at 6–10 years of age. Despite methodological differences cord omega-6 PUFA have generally been related to higher, and omega-3 PUFA to lower offspring BMI and fat-mass (Maslova et al., 2018; Standl et al., 2014; Voortman et al., 2018). In a Dutch birth-cohort, omega-6 PUFAs γ-linoleic-acid and dihomo-γ-linoleic-acid in cord plasma phospholipids were inversely associated with HOMA-IR at the age of seven (Rump et al., 2002). However, other studies did not assess insulin resistance (Maslova et al., 2018; Standl et al., 2014; Voortman et al., 2018). Cord serum DHA and total omega-3 long-chain PUFA have been positively associated with adiponectin– an adipokine closely linked to insulin sensitivity– at age of 10 years (Standl et al., 2015). In that study, a higher omega-6-to-omega-3-PUFA-ratio was related to lower adiponectin, but no other associations between omega-6 PUFA and adiponectin were observed.

Previous studies have reported adverse effects associated with cord blood omega-6 FA, and especially the omega-6-to-omega-3-ratio (Maslova et al., 2018; Standl et al., 2014; Voortman et al., 2018), but we did not observe any such associations in our cohort. Contrariwise, we found cord serum omega-6 FA to be beneficial in the insulin group, while no significant associations were found for omega-6 ratios. These discrepancies might be explained by differences in study populations. In our cohort all mothers had GDM requiring pharmacological treatment, a condition known to alter placental FA transfer (Ortega-Senovilla et al., 2020). The beneficial associations of cord blood DHA are evident in previous literature (Maslova et al., 2018; Standl et al., 2015) but in our data they were observed only in the insulin group. We therefore believe, that the associations seen in the insulin group may reflect a more physiological state, whereas under metformin exposure, the fetoplacental lipid metabolism might be altered, potentially modifying these associations.

Sphingomyelins at h30 were inversely related to oDI_Ins_ and AUC_CP/Gluc_. Sphingomyelins are not affected by GDM (White et al., 2017), and are unlikely to freely pass the placenta without degradation by phospholipases. Sphingomyelins, as measured by NMR, are not associated with insulin resistance during pregnancy (Huhtala et al., 2023) or in general (Ahola-Olli et al., 2019), but they could have beneficial effects on placental metabolism and function (Fakhr et al., 2021) yielding also long-term benefits.

Overall, lipids in very large HDL at h30 were inversely related to markers of offspring β-cell function. HDL particles participate in reverse cholesterol transport with larger particles having higher capacity. Although HDL receptors are expressed in the placenta (Cummings et al., 1982), the role of HDL in placental lipid transport remains largely elusive. In the metformin treated patients average HDL diameter and phospholipids in very large HDL were inversely related to β-cell function with significant interaction between the treatment groups. Although insulin treatment of GDM did not affect HDL size, the clear differences in serum lipid concentrations between metformin and insulin treated GDM patients (Huhtala et al., 2020) could be the confounder explaining discrepant associations to offspring β-cell function.

Due to known fetoplacental sexual dimorphism, we tested for interaction effects by offspring sex. None of the previously discussed associations demonstrated statistically significant interactions.

The effects of maternal metabolomics on offspring obesity have been demonstrated previously (Bianco et al., 2023). However, to the best of our knowledge, the effects maternal metabolomics on offspring insulin resistance and secretion have not been previously reported. Our findings suggest that, at least among women with insulin- or metformin-treated GDM, it is not only maternal glucose levels but also circulating lipids that may influence metabolic outcomes in the next generation. While our study cannot prove causal effects, it highlights the potential importance of optimizing maternal lipid profiles during pregnancy– an area that warrants greater focus in both research and clinical practice.

Previous studies on maternal omega-3 supplementation during pregnancy have not consistently demonstrated benefits for offspring health outcomes (Stratakis et al., 2014). The impact of maternal metabolism on fetal programming is likely multifactorial, and the relevance of specific metabolic pathways may vary across maternal metabolic subtypes (Francis et al., 2023). Hence, the effects of maternal metabolism on offspring health should be investigated also in other populations to account for potential confounders and biological variability. Additionally, our findings suggest that maternal treatment with insulin or metformin may influence placental nutrient transfer and/or fetal metabolism and should be further investigated.

### Strengths and limitations

This was the first study to examine the associations between maternal and cord serum lipidome and childhood insulin secretion and insulin resistance. Strengths of the study include its prospective design, the use of standardized NMR protocol with detailed lipoprotein lipid profiling, and the characterization of offspring insulin secretion and resistance using OGTT-based measures.

However, several limitations should be acknowledged. First, this was a secondary analysis of a previously conducted trial, and therefore no power calculations were performed. Additionally, loss to follow-up reduced the sample size, further limiting statistical power. Second, all children in the study were exposed to hyperglycemia in the form of GDM. This may limit the generalizability of the findings to the broader pregnant population. On the other hand, GDM is known to increase the risk of metabolic disturbances in offspring (Lowe et al., 2019), potentially resulting in a wider range of HOMA2-IR and AUC_Ins/Gluc_ & AUC_CP/Gluc_ values compared to unselected nine-year-old children, thereby increasing the likelihood of detecting associations with perinatal exposures. Third, participants in a randomized trial may not be fully representative of the background population, introducing potential selection bias. Fourth, although we were able account for perinatal confounding factors, residual confounding from unmeasured variables cannot be excluded. Fifth, due to practical constraints, outcome variables were estimated using OGTT rather than clamp studies, which are considered the gold standard. Furthermore, OGTT measurements were limited to 0, 30, and 120 min, reducing temporal resolution and potentially affecting the precision of outcome estimates. However, this limitation is unlikely to have caused systematic bias. The use C-peptide-based measures, which benefit from a longer half-life compared to insulin, could have partially mitigated this issue.

## Conclusions

To summarize, this study provided evidence for early metabolic programming *in utero* presenting as variation in insulin function in childhood. To break the vicious cycle of intergenerational diabetes, the approach to maternal metabolism and nutrition should be broadened, with greater emphasis on PUFA. Furthermore, some effects of maternal lipids on offspring health appear to be modulated by prenatal metformin exposure.

## Electronic supplementary material

Below is the link to the electronic supplementary material.

Supplementary table 1– Comparison of clinical characteristics between the study participants and subjects in the original trial not included in this analysis

Supplementary table 2– Univariate associations between clinical confounders and outcome variables

Supplementary Tables 3–5– Associations between serummetabolites and insulin resistance and β-cell function in offspring at nine years age

Supplementary Table 6– Associations between serum metabolites and offspring ISOBMI at nine years age

Supplementary Table 7– Associations between serum metabolites and outcome variables with significant treatment group interactions

Supplementary Figure 1– Associations between outcome variables in the children at the age of nine years

## Data Availability

The data sets are not publicly available because they contain information that could compromise the privacy and consent of the participants.

## Abbreviations

AUC_CP/Gluc_ Ratio of the area: Ratio of the area-under-the-C-peptide-curve to the area-under-the-glucose-curve
AUC_Ins/Gluc_ Ratio of the area: Ratio of the area-under-the-insulin-curve to the area-under-the-glucose-curve
BMI: Body mass index
DHA: Docosahexaenoic acid
pBMI: Pre-pregnancy BMI
FA: Fatty acid
GDM: Gestational diabetes
GWG: Gestational weight gain
HDL: High-density lipoprotein
HOMA2-IR: Homeostasis model assessment of insulin resistance 2
HOMA2-IR_CP_: HOMA2-IR based on C-peptide
HOMA2-IR_Ins_: HOMA2-IR based on insulin
IDL: Intermediate-density lipoprotein
ISOBMI: BMI adjusted for child’s age and sex
LDL: Low-density lipoprotein
LA: Linoleic acid
LGA: Large for gestational age
MUFA: Monounsaturated fatty acids
NMR: Nuclear magnetic resonance
SD: Standard deviation
oDI: Oral disposition index
oDI_CP_: oDI based on C-peptide
oDI_Ins_: oDI based on Insulin
OGTT: Oral glucose tolerance test
PUFA: Polyunsaturated fatty acids
SFA: Saturated fatty acids
SGA: Small for gestational age
VLDL: Very low-density lipoprotein

## Declarations

### Software availability

All analyses were carried out using readily available open-source software.
