# Supplementary material for: Maternal and umbilical cord serum lipids in gestational diabetes predict offspring insulin secretion and resistance at the age of nine years
Source: Metabolomics. 2025 Jun 22;21(4):87. doi: 10.1007/s11306-025-02281-9 (PMC12183131; doi:10.1007/s11306-025-02281-9)
Supplement: Supplementary file 1 — Supplementary table 1– Comparison of clinical characteristics between the study participants and subjects in the original trial not included in this analysis [file 11306_2025_2281_MOESM1_ESM.pdf]

Supplementary table 1 – Comparison of baseline characteristics between patients included vs. not included in the study

	Lost to follow-up		Follow-up study participants		
	n	*Mean ± SD or n (%)*	n	*Mean ± SD or n (%)*	*p-value*
Age (years)	95	31.2 ± 5.3	122	32.6 ± 5.0	0.043
BMI (kg/m^2)	95	29.4 ± 5.2	122	29.0 ± 5.4	0.7
BMI-class	95		122		0.9
Normal weight (BMI < 25 kg/m^2)		15 (16%)		22 (18%)	
Overweight (BMI 25–29.9 kg/m^2)		40 (42%)		52 (43%)	
Obese (BMI ≥ 30 kg/m^2)		40 (42%)		48 (39%)	
Smoking (n)	92	12 (13%)	120	14 (12%)	0.8
Primiparous (n)	95	44 (46%)	122	47 (39%)	0.2
Early weight gain in pregnancy (kg)	94	6.2 ± 4.8	122	5.8 ± 3.8	0.5
Weight gain in pregnancy (kg)	94	7.8 ± 5.8	122	8.0 ± 4.8	0.8
Gestational age at OGTT (weeks)	95	26.9 ± 2.6	122	26.8 ± 2.4	0.8
OGTT fasting glucose (mmol/L)	95	5.6 ± 0.5	122	5.5 ± 0.5	0.10
OGTT 1 h glucose (mmol/L)	95	11.2 ± 1.5	122	11.2 ± 1.3	>0.9
OGTT 2 h glucose (mmol/L)	95	8.1 ± 1.8	120	8.1 ± 1.8	0.8
HbA1c at baseline (%)	95	5.54 ± 0.33	122	5.46 ± 0.34	0.075
HbA1c at baseline (mmol/mol)	95	37.0 ± 3.6	122	36.1 ± 3.7	0.075
Gestational age at delivery (weeks)	95	39.3 ± 1.4	122	39.3 ± 1.6	0.8
Preterm birth (n)	95	3 (3.2%)	122	7 (5.7%)	0.5
Cesarean delivery (n)	95	10 (11%)	122	23 (19%)	0.090
Birth weight (g)	95	3,600 ± 430	122	3,590 ± 490	0.8
Birth weight (Z-score)	95	0.06 ± 1.06	122	0.07 ± 1.11	>0.9
SGA (n)	95	11 (12%)	122	14 (11%)	>0.9
LGA (n)	95	15 (16%)	122	18 (15%)	0.8
Treatment	95		122		0.5
Insulin		44 (46%)		63 (52%)	
Metformin		51 (54%)		59 (48%)	

P-values are given for t-test, Chi-square test or Fisher's exact test as appropriate. SD: standard deviation, BMI: body mass index, OGTT: oral glucose tolerance test, SGA: adjusted birth weight < 10th centile, LGA: adjusted birth weight > 90th centile.
