# Supplementary material for: Maternal and umbilical cord serum lipids in gestational diabetes predict offspring insulin secretion and resistance at the age of nine years
Source: Metabolomics. 2025 Jun 22;21(4):87. doi: 10.1007/s11306-025-02281-9 (PMC12183131; doi:10.1007/s11306-025-02281-9)
Supplement: Supplementary file 2 — Supplementary table 2– Univariate associations between clinical confounders and outcome variables [file 11306_2025_2281_MOESM2_ESM.pdf]

Supplementary table 2 – Univariate associations between clinical variables and outcomes

	**oDI (Insulin)**			**oDI (C-peptide)**			**HOMA2-IR (Insulin)**			**HOMA2-IR (C-peptide)**			**AUC (Insulin/Glucose)**			**AUC (Insulin/C-peptide)**		
	Beta (95% CI)	*p-value*	*n*	*Beta (95% CI)*	*p-value*	*n*	*Beta (95% CI)*	*p-value*	*n*	*Beta (95% CI)*	*p-value*	*n*	*Beta (95% CI)*	*p-value*	*n*	*Beta (95% CI)*	*p-value*	*n*
**Maternal**																		
Age	0.03 (-0.16, 0.21)	0.8	119	0.03 (-0.16, 0.22)	0.6	119	-0.08 (-0.26, 0.10)	0.4	122	-0.10 (-0.28, 0.08)	0.3	122	-0.09 (-0.27, 0.09)	0.3	120	-0.08 (-0.26, 0.10)	0.4	120
Smoking																		
No	–	–	103	–	–	103	–	–	106	–	–	106	–	–	104	–	–	104
Yes	0.05 (-0.14, 0.23)	0.6	14	0.05 (-0.14, 0.24)	0.5	14	0.1 (-0.08, 0.28)	0.3	14	0.16 (-0.02, 0.34)	0.09	14	0.16 (-0.02, 0.34)	0.082	14	**0.21 (0.03, 0.39)**	**0.023**	14
Parity																		
Parous	–	–	72	–	–	72	–	–	74	–	–	74	–	–	75	–	–	75
Nulliparous	**-0.42 (-0.79, -0.06)**	**0.023**	47	-0.42 (-0.79, -0.07)	0.2	47	0.47 (0.10, 0.83)	**0.012**	46	0.28 (-0.08, 0.65)	0.13	46	0.35 (-0.02, 0.72)	0.062	47	0.19 (-0.18, 0.56)	0.3	47
pBMI	0.04 (-0.14, 0.22)	0.7	119	0.09 (-0.09, 0.27)	0.3	119	0.16 (-0.02, 0.34)	0.074	122	0.13 (-0.05, 0.31)	0.14	122	0.29 (0.11, 0.46)	**0.002**	120	**0.29 (0.12, 0.47)**	**0.001**	120
pBMI																		
<25 kg/m²	–	–	21	–	–	21	–	–	22	–	–	22	–	–	22	–	–	22
25–29.9 kg/m²	-0.09 (-0.61, 0.42)	0.7	51	0.14 (-0.37, 0.65)	0.6	51	0.39 (-0.11, 0.89)	0.13	52	0.26 (-0.25, 0.76)	0.3	52	0.47 (-0.02, 0.96)	0.059	50	**0.55 (0.07, 1.0)**	**0.025**	50
≥ 30 kg/m²	0.14 (-0.38, 0.66)	0.6	47	0.41 (-0.11, 0.93)	0.12	47	**0.51 (0.01, 1.0)**	**0.047**	48	0.4 (-0.11, 0.90)	0.13	48	**0.8 (0.31, 1.3)**	**0.002**	48	**0.92 (0.43, 1.4)**	**<0.001**	48
Early GWG	0.0 (-0.19, 0.18)	>0.9	119	0.01 (-0.18, 0.19)	>0.9	119	-0.09 (-0.27, 0.09)	0.3	122	-0.13 (-0.31, 0.05)	0.2	122	-0.06 (-0.24, 0.13)	0.5	120	-0.08 (-0.26, 0.10)	0.4	120
Total GWG	0.06 (-0.13, 0.24)	0.5	119	0.07 (-0.11, 0.26)	0.4	119	-0.12 (-0.30, 0.06)	0.2	122	-0.15 (-0.32, 0.03)	0.11	122	-0.04 (-0.22, 0.14)	0.7	120	-0.04 (-0.22, 0.14)	0.6	120
Treatment																		
Insulin	–	–	61	–	–	61	–	–	63	–	–	63	–	–	61	–	–	61
Metformin	0.11 (-0.25, 0.48)	0.5	58	0.08 (-0.29, 0.44)	0.7	58	-0.11 (-0.47, 0.25)	0.5	59	-0.07 (-0.43, 0.29)	0.7	59	0.03 (-0.34, 0.39)	0.9	59	0.05 (-0.31, 0.42)	0.8	59
**Offspring**																		
Offspring sex																		
Male	–	–	57	–	–	57	–	–	59	–	–	59	–	–	58	–	–	58
Female	-0.19 (-0.56, 0.17)	0.3	62	-0.09 (-0.45, 0.28)	0.6	62	0.3 (-0.06, 0.66)	0.1	63	0.16 (-0.19, 0.52)	0.4	63	0.35 (-0.01, 0.71)	0.056	62	0.26 (-0.10, 0.62)	0.2	62
ISOBMI	0.0 (-0.19, 0.18)	>0.9	119	0.02 (-0.16, 0.21)	0.8	119	**0.49 (0.33, 0.65)**	**<0.001**	122	**0.55 (0.40, 0.70)**	**<0.001**	122	**0.57 (0.42, 0.72)**	**<0.001**	120	**0.57 (0.42, 0.72)**	**<0.001**	120
ISOBMI																		
<25 kg/m²	–	–	83	–	–	83	–	–	85	–	–	85	–	–	83	–	–	83
25–29.9 kg/m²	-0.26 (-0.68, 0.17)	0.2	29	-0.23 (-0.65, 0.20)	0.3	29	**0.87 (0.49, 1.2)**	**<0.001**	30	**0.9 (0.53, 1.3)**	**<0.001**	30	**0.78 (0.40, 1.2)**	**<0.001**	30	**0.69 (0.31, 1.1)**	**<0.001**	30
≥ 30 kg/m²	0.08 (-0.70, 0.87)	0.8	7	0.45 (-0.33, 1.2)	0.3	7	**1.3 (0.60, 2.0)**	**<0.001**	7	**1.4 (0.68, 2.1)**	**<0.001**	7	**1.4 (0.69, 2.1)**	**<0.001**	7	**1.6 (0.85, 2.3)**	**<0.001**	7

Standardised univariate regression beta-estimates (95% confidence intervals [CI]) for each association. pBMI (pre-pregnancy body mass index) and ISOBMI (offspring BMI adjusted for age and sex) are included as both continuous and categorical predictors. Associations with p-value < 0.05 are bolded. GWG: gestational weight gain, oDI: oral disposition index, HOMA2-IR: homeostasis model assessment of insulin resistance 2, AUC: area under curve during oral glucose tolerance test calculated as $AUC_{Insulin}/AUC_{Glucose}$ or $AUC_{C\text{-}peptide}/AUC_{Glucose}$.
