# Supplementary material for: Maternal and umbilical cord serum lipids in gestational diabetes predict offspring insulin secretion and resistance at the age of nine years
Source: Metabolomics. 2025 Jun 22;21(4):87. doi: 10.1007/s11306-025-02281-9 (PMC12183131; doi:10.1007/s11306-025-02281-9)
Supplement: Supplementary file 3 — Supplementary Tables 3–5– Associations between serummetabolites and insulin resistance and β-cell function in offspring at nine years age [file 11306_2025_2281_MOESM3_ESM.pdf]

Supplementary table 3 – Associations between maternal serum metabolites at approximately 30 gestational weeks, and insulin secretion and insulin resistance in offspring at nine years age

Associations are given for regression coefficients (beta) with 95% confidence intervals (CI). Adjusted model 1 is adjusted for maternal pre-pregnancy BMI. P-values below 0.01 and 0.0038 are denoted with * and **, respectively.

		HOMA2-IR (C-peptide)			HOMA2-IR (Insulin)			AUC (C-peptide/Glucose)			AUC (Insulin/Glucose)			oDI (C-peptide)			oDI (Insulin)		
**Metabolite**	**Model**	**n**	**Beta ± 95% CI**	**p-value**	**n**	**Beta ± 95% CI**	**p-value**	**n**	**Beta ± 95% CI**	**p-value**	**n**	**Beta ± 95% CI**	**p-value**	**n**	**Beta ± 95% CI**	**p-value**	**n**	**Beta ± 95% CI**	**p-value**
**Lipids**																			
Total cholesterol	unadj.	118	-0.06 [-0.24; 0.12]	0.52	118	0.01 [-0.18; 0.19]	0.94	116	-0.19 [-0.37; -0.00]	0.046	116	-0.18 [-0.36; 0.00]	0.051	116	-0.16 [-0.34; 0.03]	0.090	116	-0.22 [-0.40; -0.04]	0.018
	mod1	118	-0.07 [-0.25; 0.12]	0.46	118	-0.01 [-0.19; 0.18]	0.94	116	-0.20 [-0.38; -0.03]	0.022	116	-0.20 [-0.37; -0.02]	0.029	116	-0.16 [-0.34; 0.02]	0.088	116	-0.21 [-0.40; -0.03]	0.023
Total cholesterol minus HDL cholesterol	unadj.	118	-0.05 [-0.23; 0.13]	0.59	118	0.02 [-0.17; 0.20]	0.84	116	-0.17 [-0.35; 0.02]	0.074	116	-0.16 [-0.34; 0.02]	0.089	116	-0.13 [-0.32; 0.05]	0.16	116	-0.20 [-0.38; -0.01]	0.035
	mod1	118	-0.06 [-0.24; 0.13]	0.54	118	0.00 [-0.18; 0.19]	0.96	116	-0.18 [-0.36; -0.00]	0.045	116	-0.17 [-0.35; 0.01]	0.060	116	-0.13 [-0.32; 0.06]	0.17	116	-0.19 [-0.37; -0.00]	0.048
Remnant cholesterol	unadj.	118	-0.05 [-0.24; 0.13]	0.57	118	0.01 [-0.18; 0.19]	0.94	116	-0.17 [-0.35; 0.02]	0.072	116	-0.16 [-0.34; 0.02]	0.086	116	-0.13 [-0.31; 0.06]	0.17	116	-0.18 [-0.36; 0.00]	0.052
	mod1	118	-0.06 [-0.25; 0.12]	0.51	118	-0.01 [-0.20; 0.18]	0.92	116	-0.19 [-0.36; -0.01]	0.039	116	-0.18 [-0.36; 0.00]	0.053	116	-0.13 [-0.31; 0.06]	0.18	116	-0.17 [-0.36; 0.01]	0.071
VLDL cholesterol	unadj.	118	-0.03 [-0.22; 0.15]	0.71	118	0.01 [-0.17; 0.20]	0.88	116	-0.12 [-0.30; 0.07]	0.21	116	-0.11 [-0.29; 0.07]	0.24	116	-0.10 [-0.28; 0.09]	0.31	116	-0.14 [-0.32; 0.05]	0.15
	mod1	118	-0.05 [-0.23; 0.14]	0.63	118	-0.01 [-0.19; 0.18]	0.95	116	-0.14 [-0.32; 0.04]	0.13	116	-0.13 [-0.31; 0.06]	0.17	116	-0.09 [-0.28; 0.10]	0.33	116	-0.12 [-0.31; 0.07]	0.20
Clinical LDL cholesterol	unadj.	118	-0.05 [-0.23; 0.14]	0.62	118	0.03 [-0.15; 0.21]	0.75	116	-0.17 [-0.35; 0.01]	0.067	116	-0.16 [-0.35; 0.02]	0.080	116	-0.14 [-0.32; 0.04]	0.13	116	-0.22 [-0.40; -0.03]	0.020
	mod1	118	-0.05 [-0.24; 0.13]	0.59	118	0.02 [-0.16; 0.20]	0.82	116	-0.18 [-0.35; -0.00]	0.047	116	-0.17 [-0.35; 0.01]	0.061	116	-0.14 [-0.32; 0.05]	0.14	116	-0.21 [-0.39; -0.02]	0.026
LDL cholesterol	unadj.	118	-0.05 [-0.23; 0.14]	0.62	118	0.03 [-0.15; 0.21]	0.75	116	-0.16 [-0.35; 0.02]	0.081	116	-0.16 [-0.34; 0.03]	0.096	116	-0.14 [-0.32; 0.05]	0.15	116	-0.21 [-0.39; -0.03]	0.025
	mod1	118	-0.05 [-0.24; 0.13]	0.58	118	0.02 [-0.17; 0.20]	0.84	116	-0.17 [-0.35; 0.00]	0.056	116	-0.16 [-0.34; 0.02]	0.072	116	-0.13 [-0.32; 0.05]	0.16	116	-0.20 [-0.38; -0.01]	0.034
HDL cholesterol	unadj.	118	-0.04 [-0.22; 0.15]	0.69	118	-0.05 [-0.23; 0.13]	0.60	116	-0.07 [-0.25; 0.12]	0.49	116	-0.08 [-0.26; 0.11]	0.41	116	-0.09 [-0.28; 0.09]	0.32	116	-0.08 [-0.26; 0.11]	0.42
	mod1	118	-0.04 [-0.23; 0.14]	0.64	118	-0.05 [-0.24; 0.13]	0.58	116	-0.09 [-0.27; 0.09]	0.33	116	-0.10 [-0.28; 0.08]	0.28	116	-0.12 [-0.30; 0.07]	0.21	116	-0.10 [-0.29; 0.09]	0.30
Total triglycerides	unadj.	118	0.02 [-0.17; 0.20]	0.87	118	0.01 [-0.17; 0.20]	0.90	116	0.03 [-0.16; 0.21]	0.77	116	0.02 [-0.16; 0.21]	0.80	116	-0.02 [-0.20; 0.17]	0.87	116	-0.00 [-0.19; 0.18]	0.96
	mod1	118	0.00 [-0.19; 0.19]	0.98	118	-0.02 [-0.20; 0.17]	0.86	116	0.00 [-0.18; 0.19]	0.97	116	0.00 [-0.18; 0.19]	0.98	116	-0.01 [-0.21; 0.18]	0.88	116	0.01 [-0.18; 0.21]	0.88
Triglycerides in VLDL	unadj.	118	0.03 [-0.16; 0.21]	0.76	118	0.02 [-0.17; 0.20]	0.86	116	0.05 [-0.13; 0.24]	0.58	116	0.05 [-0.14; 0.23]	0.60	116	-0.00 [-0.19; 0.18]	0.98	116	0.02 [-0.17; 0.20]	0.86
	mod1	118	0.02 [-0.17; 0.21]	0.86	118	-0.01 [-0.20; 0.18]	0.92	116	0.03 [-0.15; 0.21]	0.72	116	0.03 [-0.15; 0.22]	0.74	116	0.00 [-0.19; 0.19]	>0.99	116	0.04 [-0.15; 0.23]	0.71
Triglycerides in LDL	unadj.	118	-0.05 [-0.23; 0.14]	0.63	118	-0.01 [-0.19; 0.18]	0.95	116	-0.10 [-0.29; 0.08]	0.27	116	-0.10 [-0.29; 0.08]	0.27	116	-0.07 [-0.25; 0.12]	0.46	116	-0.10 [-0.29; 0.08]	0.27
	mod1	118	-0.06 [-0.25; 0.13]	0.52	118	-0.03 [-0.22; 0.16]	0.74	116	-0.13 [-0.31; 0.04]	0.14	116	-0.13 [-0.31; 0.05]	0.16	116	-0.07 [-0.26; 0.11]	0.44	116	-0.09 [-0.28; 0.10]	0.33
Triglycerides in HDL	unadj.	118	-0.03 [-0.21; 0.16]	0.77	118	-0.02 [-0.21; 0.16]	0.79	116	-0.05 [-0.23; 0.14]	0.63	116	-0.05 [-0.24; 0.13]	0.56	116	-0.06 [-0.25; 0.12]	0.49	116	-0.06 [-0.24; 0.13]	0.55
	mod1	118	-0.05 [-0.24; 0.14]	0.62	118	-0.06 [-0.25; 0.13]	0.54	116	-0.08 [-0.27; 0.10]	0.36	116	-0.09 [-0.27; 0.10]	0.34	116	-0.07 [-0.26; 0.12]	0.45	116	-0.04 [-0.24; 0.15]	0.65
Total phospholipids in lipoprotein particles	unadj.	118	-0.05 [-0.23; 0.13]	0.58	118	-0.02 [-0.20; 0.17]	0.86	116	-0.14 [-0.32; 0.04]	0.13	116	-0.15 [-0.33; 0.04]	0.12	116	-0.15 [-0.34; 0.03]	0.10	116	-0.18 [-0.36; 0.00]	0.055
	mod1	118	-0.07 [-0.25; 0.12]	0.47	118	-0.04 [-0.23; 0.14]	0.65	116	-0.18 [-0.35; -0.00]	0.047	116	-0.18 [-0.36; -0.00]	0.046	116	-0.17 [-0.35; 0.02]	0.075	116	-0.18 [-0.36; 0.01]	0.059
Phospholipids in VLDL	unadj.	118	-0.02 [-0.20; 0.17]	0.86	118	0.02 [-0.17; 0.20]	0.86	116	-0.06 [-0.24; 0.13]	0.53	116	-0.06 [-0.24; 0.13]	0.55	116	-0.06 [-0.25; 0.12]	0.52	116	-0.09 [-0.27; 0.10]	0.35
	mod1	118	-0.03 [-0.22; 0.16]	0.76	118	-0.01 [-0.20; 0.18]	0.94	116	-0.08 [-0.26; 0.10]	0.38	116	-0.08 [-0.26; 0.11]	0.42	116	-0.06 [-0.25; 0.13]	0.55	116	-0.07 [-0.26; 0.12]	0.47
Phospholipids in LDL	unadj.	118	-0.05 [-0.23; 0.14]	0.62	118	0.03 [-0.16; 0.21]	0.76	116	-0.16 [-0.35; 0.02]	0.077	116	-0.16 [-0.34; 0.03]	0.090	116	-0.14 [-0.32; 0.05]	0.14	116	-0.21 [-0.39; -0.03]	0.023
	mod1	118	-0.05 [-0.24; 0.13]	0.59	118	0.02 [-0.17; 0.20]	0.85	116	-0.17 [-0.35; 0.00]	0.054	116	-0.17 [-0.34; 0.01]	0.070	116	-0.13 [-0.32; 0.05]	0.15	116	-0.20 [-0.38; -0.02]	0.032
Phospholipids in HDL	unadj.	118	-0.02 [-0.20; 0.16]	0.82	118	-0.06 [-0.24; 0.13]	0.54	116	-0.02 [-0.21; 0.17]	0.83	116	-0.04 [-0.23; 0.15]	0.67	116	-0.08 [-0.26; 0.11]	0.42	116	-0.03 [-0.22; 0.15]	0.73
	mod1	118	-0.03 [-0.22; 0.15]	0.72	118	-0.07 [-0.26; 0.11]	0.45	116	-0.06 [-0.24; 0.12]	0.52	116	-0.07 [-0.26; 0.11]	0.42	116	-0.10 [-0.29; 0.08]	0.27	116	-0.06 [-0.24; 0.13]	0.56
Total lipids in lipoprotein particles	unadj.	118	-0.04 [-0.22; 0.14]	0.66	118	0.00 [-0.18; 0.19]	0.96	116	-0.13 [-0.31; 0.06]	0.17	116	-0.13 [-0.31; 0.06]	0.17	116	-0.13 [-0.31; 0.05]	0.16	116	-0.17 [-0.35; 0.02]	0.073
	mod1	118	-0.06 [-0.24; 0.13]	0.56	118	-0.02 [-0.21; 0.17]	0.84	116	-0.16 [-0.34; 0.02]	0.079	116	-0.16 [-0.34; 0.02]	0.089	116	-0.14 [-0.32; 0.05]	0.15	116	-0.16 [-0.35; 0.03]	0.093
Total lipids in VLDL	unadj.	118	-0.00 [-0.18; 0.18]	>0.99	118	0.02 [-0.17; 0.20]	0.86	116	-0.02 [-0.21; 0.16]	0.79	116	-0.02 [-0.21; 0.16]	0.80	116	-0.05 [-0.23; 0.14]	0.62	116	-0.06 [-0.24; 0.13]	0.55
	mod1	118	-0.01 [-0.20; 0.18]	0.89	118	-0.01 [-0.20; 0.18]	0.93	116	-0.05 [-0.23; 0.14]	0.61	116	-0.04 [-0.23; 0.14]	0.64	116	-0.04 [-0.23; 0.15]	0.65	116	-0.04 [-0.23; 0.15]	0.70
Total lipids in LDL	unadj.	118	-0.05 [-0.23; 0.14]	0.61	118	0.03 [-0.16; 0.21]	0.77	116	-0.16 [-0.35; 0.02]	0.082	116	-0.15 [-0.34; 0.03]	0.097	116	-0.13 [-0.32; 0.05]	0.15	116	-0.21 [-0.39; -0.02]	0.027
	mod1	118	-0.05 [-0.24; 0.13]	0.57	118	0.02 [-0.17; 0.20]	0.87	116	-0.17 [-0.35; 0.00]	0.054	116	-0.16 [-0.34; 0.01]	0.070	116	-0.13 [-0.32; 0.05]	0.17	116	-0.20 [-0.38; -0.01]	0.037
Total lipids in HDL	unadj.	118	-0.03 [-0.22; 0.15]	0.73	118	-0.06 [-0.24; 0.13]	0.55	116	-0.05 [-0.23; 0.14]	0.62	116	-0.06 [-0.25; 0.12]	0.50	116	-0.09 [-0.28; 0.09]	0.33	116	-0.06 [-0.24; 0.13]	0.53
	mod1	118	-0.04 [-0.23; 0.14]	0.64	118	-0.07 [-0.25; 0.12]	0.48	116	-0.08 [-0.26; 0.10]	0.37	116	-0.09 [-0.28; 0.09]	0.30	116	-0.12 [-0.30; 0.07]	0.21	116	-0.08 [-0.27; 0.11]	0.39
Total concentration of lipoprotein particles	unadj.	118	-0.00 [-0.19; 0.18]	0.98	118	0.01 [-0.17; 0.19]	0.92	116	-0.03 [-0.22; 0.15]	0.71	116	-0.06 [-0.24; 0.13]	0.55	116	-0.13 [-0.32; 0.05]	0.16	116	-0.15 [-0.33; 0.03]	0.11
	mod1	118	-0.02 [-0.20; 0.17]	0.87	118	-0.01 [-0.19; 0.18]	0.95	116	-0.07 [-0.25; 0.11]	0.45	116	-0.09 [-0.27; 0.09]	0.34	116	-0.15 [-0.33; 0.03]	0.11	116	-0.16 [-0.35; 0.02]	0.087
Concentration of VLDL particles	unadj.	118	-0.03 [-0.21; 0.15]	0.74	118	0.02 [-0.17; 0.20]	0.86	116	-0.10 [-0.28; 0.09]	0.31	116	-0.09 [-0.28; 0.09]	0.33	116	-0.08 [-0.27; 0.10]	0.37	116	-0.12 [-0.31; 0.06]	0.18
	mod1	118	-0.04 [-0.23; 0.15]	0.65	118	-0.00 [-0.19; 0.18]	0.96	116	-0.12 [-0.30; 0.06]	0.20	116	-0.11 [-0.30; 0.07]	0.24	116	-0.08 [-0.27; 0.11]	0.40	116	-0.11 [-0.30; 0.08]	0.25
Concentration of LDL particles	unadj.	118	-0.05 [-0.23; 0.13]	0.59	118	0.02 [-0.16; 0.21]	0.81	116	-0.16 [-0.35; 0.02]	0.078	116	-0.16 [-0.34; 0.03]	0.095	116	-0.13 [-0.31; 0.05]	0.17	116	-0.20 [-0.38; -0.01]	0.035
	mod1	118	-0.06 [-0.24; 0.13]	0.55	118	0.01 [-0.18; 0.19]	0.92	116	-0.18 [-0.35; 0.00]	0.050	116	-0.17 [-0.35; 0.01]	0.068	116	-0.13 [-0.31; 0.06]	0.18	116	-0.19 [-0.37; -0.00]	0.050
Concentration of HDL particles	unadj.	118	0.01 [-0.17; 0.19]	0.91	118	0.01 [-0.18; 0.19]	0.95	116	0.01 [-0.18; 0.19]	0.96	116	-0.02 [-0.20; 0.17]	0.84	116	-0.10 [-0.29; 0.08]	0.27	116	-0.11 [-0.29; 0.08]	0.26
	mod1	118	-0.00 [-0.19; 0.18]	0.99	118	-0.01 [-0.19; 0.18]	0.94	116	-0.03 [-0.21; 0.15]	0.76	116	-0.05 [-0.23; 0.13]	0.60	116	-0.13 [-0.31; 0.06]	0.18	116	-0.12 [-0.31; 0.06]	0.20
Average diameter for VLDL particles	unadj.	118	0.04 [-0.14; 0.22]	0.67	118	-0.00 [-0.19; 0.18]	0.98	116	0.14 [-0.04; 0.32]	0.14	116	0.12 [-0.06; 0.31]	0.18	116	0.06 [-0.13; 0.24]	0.54	116	0.10 [-0.08; 0.29]	0.27
	mod1	118	0.03 [-0.16; 0.22]	0.73	118	-0.02 [-0.21; 0.16]	0.81	116	0.13 [-0.05; 0.31]	0.16	116	0.12 [-0.07; 0.30]	0.21	116	0.06 [-0.12; 0.25]	0.50	116	0.12 [-0.06; 0.31]	0.19
Average diameter for LDL particles	unadj.	118	-0.10 [-0.29; 0.08]	0.26	118	0.01 [-0.18; 0.19]	0.96	116	-0.19 [-0.38; -0.01]	0.037	116	-0.20 [-0.38; -0.01]	0.036	116	-0.10 [-0.28; 0.09]	0.30	116	-0.20 [-0.38; -0.01]	0.035
	mod1	118	-0.10 [-0.29; 0.08]	0.26	118	0.01 [-0.18; 0.19]	0.95	116	-0.19 [-0.37; -0.02]	0.029	116	-0.19 [-0.37; -0.02]	0.030	116	-0.10 [-0.28; 0.09]	0.30	116	-0.20 [-0.38; -0.01]	0.034
Average diameter for HDL particles	unadj.	118	-0.14 [-0.32; 0.05]	0.14	118	-0.16 [-0.34; 0.02]	0.079	116	-0.19 [-0.37; -0.00]	0.044	116	-0.19 [-0.37; -0.00]	0.046	116	-0.07 [-0.25; 0.12]	0.48	116	-0.00 [-0.19; 0.18]	0.98
	mod1	118	-0.14 [-0.32; 0.04]	0.14	118	-0.16 [-0.34; 0.02]	0.080	116	-0.20 [-0.37; -0.02]	0.027	116	-0.19 [-0.37; -0.02]	0.031	116	-0.08 [-0.26; 0.11]	0.40	116	-0.02 [-0.20; 0.17]	0.87

		HOMA2-IR (C-peptide)			HOMA2-IR (Insulin)			AUC (C-peptide/Glucose)			AUC (Insulin/Glucose)			oDI (C-peptide)			oDI (Insulin)		
**Metabolite**	**Model**	**n**	**Beta ± 95% CI**	**p-value**	**n**	**Beta ± 95% CI**	**p-value**	**n**	**Beta ± 95% CI**	**p-value**	**n**	**Beta ± 95% CI**	**p-value**	**n**	**Beta ± 95% CI**	**p-value**	**n**	**Beta ± 95% CI**	**p-value**
**Detailed lipoprotein contents**																			
Concentration of XXL-VLDL particles	unadj.	118	0.04 [-0.14; 0.23]	0.64	118	0.01 [-0.18; 0.19]	0.95	116	0.10 [-0.09; 0.28]	0.30	116	0.10 [-0.09; 0.28]	0.29	116	0.03 [-0.15; 0.22]	0.72	116	0.08 [-0.10; 0.27]	0.37
	mod1	118	0.03 [-0.16; 0.22]	0.76	118	-0.02 [-0.21; 0.16]	0.81	116	0.07 [-0.11; 0.25]	0.45	116	0.07 [-0.11; 0.26]	0.43	116	0.03 [-0.16; 0.22]	0.75	116	0.10 [-0.09; 0.29]	0.30
Concentration of XL-VLDL particles	unadj.	118	0.02 [-0.16; 0.21]	0.80	118	0.02 [-0.17; 0.20]	0.85	116	0.05 [-0.14; 0.23]	0.61	116	0.04 [-0.14; 0.23]	0.63	116	0.00 [-0.18; 0.19]	0.98	116	0.02 [-0.17; 0.20]	0.87
	mod1	118	0.01 [-0.18; 0.20]	0.90	118	-0.01 [-0.20; 0.18]	0.93	116	0.03 [-0.15; 0.21]	0.76	116	0.03 [-0.16; 0.21]	0.76	116	0.01 [-0.18; 0.20]	0.95	116	0.04 [-0.16; 0.23]	0.71
Concentration of L-VLDL particles	unadj.	118	0.02 [-0.16; 0.21]	0.80	118	0.03 [-0.16; 0.21]	0.77	116	0.03 [-0.16; 0.22]	0.75	116	0.03 [-0.16; 0.21]	0.76	116	-0.02 [-0.20; 0.17]	0.85	116	-0.02 [-0.20; 0.17]	0.86
	mod1	118	0.01 [-0.18; 0.20]	0.89	118	0.00 [-0.19; 0.19]	0.97	116	0.02 [-0.17; 0.20]	0.87	116	0.02 [-0.17; 0.20]	0.87	116	-0.01 [-0.20; 0.18]	0.90	116	0.01 [-0.19; 0.20]	0.96
Concentration of M-VLDL particles	unadj.	118	-0.02 [-0.20; 0.17]	0.86	118	0.03 [-0.15; 0.22]	0.71	116	-0.09 [-0.28; 0.09]	0.33	116	-0.09 [-0.27; 0.10]	0.36	116	-0.09 [-0.28; 0.09]	0.31	116	-0.14 [-0.32; 0.04]	0.13
	mod1	118	-0.03 [-0.22; 0.16]	0.79	118	0.02 [-0.17; 0.20]	0.86	116	-0.11 [-0.29; 0.07]	0.24	116	-0.10 [-0.28; 0.08]	0.28	116	-0.09 [-0.28; 0.10]	0.34	116	-0.13 [-0.32; 0.06]	0.19
Concentration of S-VLDL particles	unadj.	118	-0.03 [-0.21; 0.16]	0.76	118	0.02 [-0.16; 0.21]	0.80	116	-0.08 [-0.27; 0.10]	0.37	116	-0.08 [-0.26; 0.11]	0.40	116	-0.08 [-0.26; 0.11]	0.41	116	-0.12 [-0.31; 0.06]	0.19
	mod1	118	-0.04 [-0.23; 0.15]	0.70	118	0.00 [-0.19; 0.19]	0.96	116	-0.10 [-0.28; 0.09]	0.29	116	-0.09 [-0.28; 0.09]	0.33	116	-0.07 [-0.26; 0.12]	0.47	116	-0.11 [-0.30; 0.09]	0.28
Concentration of XS-VLDL particles	unadj.	118	-0.07 [-0.25; 0.12]	0.48	118	-0.01 [-0.19; 0.18]	0.94	116	-0.16 [-0.34; 0.02]	0.085	116	-0.16 [-0.34; 0.03]	0.095	116	-0.10 [-0.29; 0.08]	0.26	116	-0.16 [-0.34; 0.03]	0.095
	mod1	118	-0.08 [-0.27; 0.11]	0.40	118	-0.03 [-0.21; 0.16]	0.76	116	-0.19 [-0.36; -0.01]	0.040	116	-0.18 [-0.36; 0.00]	0.052	116	-0.11 [-0.29; 0.08]	0.26	116	-0.15 [-0.33; 0.04]	0.12
Concentration of IDL particles	unadj.	118	-0.06 [-0.25; 0.12]	0.49	118	0.00 [-0.18; 0.19]	0.99	116	-0.20 [-0.38; -0.02]	0.032	116	-0.19 [-0.38; -0.01]	0.038	116	-0.14 [-0.32; 0.05]	0.14	116	-0.20 [-0.38; -0.02]	0.029
	mod1	118	-0.07 [-0.26; 0.11]	0.45	118	-0.01 [-0.19; 0.17]	0.91	116	-0.21 [-0.39; -0.04]	0.016	116	-0.21 [-0.38; -0.03]	0.023	116	-0.14 [-0.32; 0.05]	0.14	116	-0.20 [-0.38; -0.01]	0.036
Concentration of L-LDL particles	unadj.	118	-0.06 [-0.24; 0.13]	0.54	118	0.02 [-0.16; 0.20]	0.82	116	-0.18 [-0.36; 0.01]	0.058	116	-0.17 [-0.35; 0.02]	0.072	116	-0.14 [-0.32; 0.05]	0.15	116	-0.21 [-0.39; -0.03]	0.026
	mod1	118	-0.06 [-0.25; 0.12]	0.50	118	0.01 [-0.18; 0.19]	0.93	116	-0.19 [-0.36; -0.01]	0.036	116	-0.18 [-0.36; 0.00]	0.051	116	-0.13 [-0.32; 0.05]	0.16	116	-0.20 [-0.38; -0.01]	0.037
Concentration of M-LDL particles	unadj.	118	-0.03 [-0.22; 0.15]	0.71	118	0.03 [-0.15; 0.21]	0.74	116	-0.14 [-0.32; 0.04]	0.14	116	-0.13 [-0.32; 0.05]	0.16	116	-0.12 [-0.30; 0.07]	0.21	116	-0.18 [-0.36; 0.00]	0.052
	mod1	118	-0.04 [-0.23; 0.15]	0.66	118	0.02 [-0.17; 0.20]	0.86	116	-0.15 [-0.33; 0.03]	0.092	116	-0.14 [-0.33; 0.04]	0.11	116	-0.12 [-0.30; 0.07]	0.22	116	-0.17 [-0.36; 0.02]	0.072
Concentration of S-LDL particles	unadj.	118	-0.05 [-0.23; 0.14]	0.62	118	0.01 [-0.17; 0.20]	0.87	116	-0.15 [-0.34; 0.03]	0.098	116	-0.14 [-0.33; 0.04]	0.12	116	-0.12 [-0.30; 0.07]	0.21	116	-0.17 [-0.35; 0.01]	0.065
	mod1	118	-0.05 [-0.24; 0.13]	0.57	118	0.00 [-0.19; 0.19]	>0.99	116	-0.17 [-0.34; 0.01]	0.065	116	-0.16 [-0.34; 0.02]	0.089	116	-0.11 [-0.30; 0.07]	0.23	116	-0.16 [-0.35; 0.03]	0.091
Concentration of XL-HDL particles	unadj.	118	-0.12 [-0.30; 0.06]	0.20	118	-0.12 [-0.30; 0.06]	0.20	116	-0.24 [-0.42; -0.06]	0.011	116	-0.23 [-0.41; -0.05]	0.014	116	-0.14 [-0.32; 0.05]	0.14	116	-0.10 [-0.29; 0.08]	0.28
	mod1	118	-0.12 [-0.31; 0.06]	0.18	118	-0.12 [-0.30; 0.06]	0.18	116	-0.25 [-0.42; -0.08]	0.0049 *	116	-0.24 [-0.41; -0.06]	0.0076 *	116	-0.15 [-0.33; 0.04]	0.12	116	-0.11 [-0.29; 0.08]	0.25
Concentration of L-HDL particles	unadj.	118	-0.09 [-0.27; 0.09]	0.34	118	-0.10 [-0.29; 0.08]	0.26	116	-0.17 [-0.35; 0.02]	0.075	116	-0.17 [-0.35; 0.02]	0.072	116	-0.12 [-0.31; 0.06]	0.19	116	-0.08 [-0.27; 0.10]	0.38
	mod1	118	-0.10 [-0.28; 0.09]	0.31	118	-0.11 [-0.29; 0.07]	0.24	116	-0.19 [-0.36; -0.01]	0.038	116	-0.19 [-0.36; -0.01]	0.041	116	-0.14 [-0.32; 0.04]	0.13	116	-0.10 [-0.28; 0.09]	0.30
Concentration of M-HDL particles	unadj.	118	0.01 [-0.18; 0.19]	0.93	118	-0.02 [-0.20; 0.17]	0.85	116	0.04 [-0.15; 0.22]	0.70	116	0.01 [-0.17; 0.20]	0.90	116	-0.06 [-0.25; 0.12]	0.50	116	-0.04 [-0.23; 0.14]	0.65
	mod1	118	-0.00 [-0.19; 0.18]	0.96	118	-0.03 [-0.22; 0.15]	0.74	116	-0.00 [-0.18; 0.18]	0.98	116	-0.02 [-0.20; 0.16]	0.81	116	-0.09 [-0.28; 0.09]	0.33	116	-0.06 [-0.25; 0.12]	0.50
Concentration of S-HDL particles	unadj.	118	0.09 [-0.09; 0.27]	0.34	118	0.11 [-0.07; 0.29]	0.24	116	0.13 [-0.06; 0.31]	0.17	116	0.10 [-0.08; 0.29]	0.28	116	-0.04 [-0.23; 0.15]	0.67	116	-0.09 [-0.28; 0.09]	0.33
	mod1	118	0.08 [-0.10; 0.27]	0.38	118	0.10 [-0.08; 0.28]	0.28	116	0.11 [-0.06; 0.29]	0.21	116	0.09 [-0.09; 0.27]	0.34	116	-0.05 [-0.23; 0.14]	0.63	116	-0.09 [-0.28; 0.09]	0.33
Total lipids in XXL-VLDL	unadj.	118	0.04 [-0.14; 0.23]	0.63	118	0.00 [-0.18; 0.19]	0.98	116	0.10 [-0.08; 0.29]	0.26	116	0.11 [-0.08; 0.29]	0.26	116	0.04 [-0.14; 0.23]	0.64	116	0.10 [-0.09; 0.28]	0.29
	mod1	118	0.03 [-0.16; 0.22]	0.75	118	-0.03 [-0.21; 0.16]	0.78	116	0.07 [-0.11; 0.25]	0.42	116	0.08 [-0.10; 0.26]	0.40	116	0.04 [-0.15; 0.23]	0.68	116	0.11 [-0.08; 0.30]	0.24
Total lipids in XL-VLDL	unadj.	118	0.03 [-0.15; 0.21]	0.75	118	0.02 [-0.17; 0.20]	0.85	116	0.05 [-0.13; 0.24]	0.57	116	0.05 [-0.13; 0.24]	0.58	116	0.00 [-0.18; 0.19]	>0.99	116	0.02 [-0.17; 0.21]	0.83
	mod1	118	0.02 [-0.17; 0.21]	0.85	118	-0.01 [-0.20; 0.18]	0.93	116	0.03 [-0.15; 0.22]	0.71	116	0.03 [-0.15; 0.22]	0.71	116	0.00 [-0.19; 0.19]	0.97	116	0.04 [-0.15; 0.23]	0.68
Total lipids in L-VLDL	unadj.	118	0.03 [-0.16; 0.21]	0.78	118	0.03 [-0.16; 0.21]	0.79	116	0.03 [-0.15; 0.22]	0.74	116	0.03 [-0.16; 0.21]	0.76	116	-0.02 [-0.21; 0.17]	0.83	116	-0.01 [-0.20; 0.17]	0.88
	mod1	118	0.02 [-0.17; 0.21]	0.86	118	0.00 [-0.19; 0.19]	0.98	116	0.02 [-0.17; 0.20]	0.86	116	0.02 [-0.17; 0.20]	0.86	116	-0.01 [-0.21; 0.18]	0.88	116	0.01 [-0.18; 0.20]	0.93
Total lipids in M-VLDL	unadj.	118	-0.01 [-0.19; 0.17]	0.91	118	0.03 [-0.15; 0.21]	0.75	116	-0.07 [-0.26; 0.11]	0.45	116	-0.07 [-0.25; 0.12]	0.47	116	-0.09 [-0.27; 0.10]	0.36	116	-0.12 [-0.30; 0.06]	0.20
	mod1	118	-0.02 [-0.21; 0.17]	0.84	118	0.01 [-0.18; 0.20]	0.92	116	-0.09 [-0.27; 0.09]	0.34	116	-0.08 [-0.27; 0.10]	0.37	116	-0.08 [-0.27; 0.11]	0.40	116	-0.10 [-0.29; 0.09]	0.28
Total lipids in S-VLDL	unadj.	118	-0.03 [-0.22; 0.15]	0.72	118	0.02 [-0.17; 0.20]	0.84	116	-0.10 [-0.28; 0.09]	0.29	116	-0.09 [-0.28; 0.09]	0.32	116	-0.08 [-0.27; 0.10]	0.37	116	-0.13 [-0.31; 0.05]	0.16
	mod1	118	-0.04 [-0.23; 0.15]	0.66	118	-0.00 [-0.19; 0.19]	>0.99	116	-0.11 [-0.30; 0.07]	0.22	116	-0.11 [-0.29; 0.08]	0.25	116	-0.08 [-0.27; 0.11]	0.41	116	-0.11 [-0.30; 0.08]	0.24
Total lipids in XS-VLDL	unadj.	118	-0.07 [-0.25; 0.11]	0.45	118	-0.01 [-0.20; 0.17]	0.88	116	-0.16 [-0.35; 0.02]	0.080	116	-0.16 [-0.34; 0.02]	0.088	116	-0.10 [-0.29; 0.08]	0.27	116	-0.15 [-0.33; 0.03]	0.11
	mod1	118	-0.08 [-0.27; 0.10]	0.37	118	-0.04 [-0.22; 0.15]	0.70	116	-0.19 [-0.37; -0.01]	0.036	116	-0.18 [-0.36; -0.00]	0.046	116	-0.11 [-0.29; 0.08]	0.26	116	-0.14 [-0.33; 0.05]	0.14
Total lipids in IDL	unadj.	118	-0.07 [-0.26; 0.11]	0.43	118	-0.01 [-0.19; 0.18]	0.94	116	-0.21 [-0.39; -0.03]	0.023	116	-0.21 [-0.39; -0.02]	0.026	116	-0.15 [-0.34; 0.03]	0.10	116	-0.21 [-0.40; -0.03]	0.021
	mod1	118	-0.08 [-0.26; 0.10]	0.39	118	-0.02 [-0.20; 0.16]	0.84	116	-0.23 [-0.40; -0.05]	0.011	116	-0.22 [-0.39; -0.04]	0.015	116	-0.15 [-0.34; 0.03]	0.098	116	-0.21 [-0.39; -0.03]	0.025
Total lipids in L-LDL	unadj.	118	-0.05 [-0.24; 0.13]	0.57	118	0.02 [-0.16; 0.21]	0.79	116	-0.17 [-0.36; 0.01]	0.061	116	-0.17 [-0.35; 0.02]	0.072	116	-0.14 [-0.33; 0.04]	0.13	116	-0.22 [-0.40; -0.04]	0.020
	mod1	118	-0.06 [-0.25; 0.13]	0.52	118	0.01 [-0.17; 0.20]	0.89	116	-0.19 [-0.36; -0.01]	0.039	116	-0.18 [-0.36; 0.00]	0.051	116	-0.14 [-0.32; 0.05]	0.14	116	-0.21 [-0.39; -0.02]	0.027
Total lipids in M-LDL	unadj.	118	-0.03 [-0.22; 0.15]	0.74	118	0.04 [-0.15; 0.22]	0.69	116	-0.13 [-0.32; 0.05]	0.15	116	-0.13 [-0.31; 0.06]	0.17	116	-0.12 [-0.30; 0.07]	0.21	116	-0.19 [-0.37; -0.00]	0.045
	mod1	118	-0.04 [-0.22; 0.15]	0.69	118	0.03 [-0.16; 0.21]	0.79	116	-0.14 [-0.32; 0.03]	0.11	116	-0.14 [-0.32; 0.04]	0.13	116	-0.11 [-0.30; 0.07]	0.23	116	-0.18 [-0.36; 0.01]	0.063
Total lipids in S-LDL	unadj.	118	-0.05 [-0.23; 0.14]	0.61	118	0.02 [-0.16; 0.20]	0.84	116	-0.16 [-0.34; 0.03]	0.091	116	-0.15 [-0.33; 0.04]	0.11	116	-0.12 [-0.31; 0.06]	0.19	116	-0.18 [-0.37; -0.00]	0.049
	mod1	118	-0.05 [-0.24; 0.13]	0.56	118	0.01 [-0.18; 0.19]	0.94	116	-0.17 [-0.34; 0.01]	0.063	116	-0.16 [-0.34; 0.02]	0.086	116	-0.12 [-0.30; 0.07]	0.22	116	-0.17 [-0.36; 0.01]	0.070
Total lipids in XL-HDL	unadj.	118	-0.12 [-0.30; 0.07]	0.21	118	-0.12 [-0.31; 0.06]	0.18	116	-0.22 [-0.40; -0.04]	0.017	116	-0.21 [-0.39; -0.03]	0.022	116	-0.12 [-0.30; 0.06]	0.20	116	-0.08 [-0.26; 0.11]	0.42
	mod1	118	-0.12 [-0.30; 0.07]	0.20	118	-0.12 [-0.31; 0.06]	0.18	116	-0.23 [-0.40; -0.06]	0.0089 *	116	-0.22 [-0.40; -0.05]	0.014	116	-0.13 [-0.31; 0.05]	0.16	116	-0.09 [-0.27; 0.10]	0.36
Total lipids in L-HDL	unadj.	118	-0.08 [-0.26; 0.10]	0.39	118	-0.10 [-0.28; 0.08]	0.28	116	-0.14 [-0.32; 0.04]	0.13	116	-0.14 [-0.33; 0.04]	0.12	116	-0.10 [-0.29; 0.08]	0.26	116	-0.06 [-0.25; 0.12]	0.50
	mod1	118	-0.09 [-0.27; 0.10]	0.35	118	-0.10 [-0.29; 0.08]	0.27	116	-0.16 [-0.34; 0.01]	0.073	116	-0.16 [-0.34; 0.02]	0.073	116	-0.12 [-0.31; 0.06]	0.19	116	-0.08 [-0.27; 0.11]	0.39
Total lipids in M-HDL	unadj.	118	0.03 [-0.15; 0.21]	0.76	118	-0.00 [-0.19; 0.18]	0.96	116	0.08 [-0.10; 0.27]	0.38	116	0.06 [-0.13; 0.24]	0.55	116	-0.03 [-0.22; 0.15]	0.74	116	-0.01 [-0.20; 0.18]	0.92
	mod1	118	0.02 [-0.17; 0.20]	0.86	118	-0.02 [-0.20; 0.17]	0.86	116	0.05 [-0.13; 0.23]	0.61	116	0.02 [-0.16; 0.21]	0.80	116	-0.06 [-0.25; 0.13]	0.52	116	-0.03 [-0.22; 0.16]	0.73
Total lipids in S-HDL	unadj.	118	0.10 [-0.08; 0.28]	0.29	118	0.10 [-0.09; 0.28]	0.30	116	0.18 [-0.01; 0.36]	0.057	116	0.14 [-0.04; 0.33]	0.12	116	-0.00 [-0.19; 0.18]	0.98	116	-0.03 [-0.22; 0.15]	0.73
	mod1	118	0.09 [-0.10; 0.27]	0.35	118	0.08 [-0.10; 0.26]	0.39	116	0.15 [-0.03; 0.32]	0.098	116	0.12 [-0.06; 0.30]	0.19	116	-0.02 [-0.20; 0.17]	0.87	116	-0.04 [-0.22; 0.15]	0.70
Phospholipids in XXL-VLDL	unadj.	118	0.04 [-0.15; 0.22]	0.69	118	0.00 [-0.18; 0.18]	>0.99	116	0.09 [-0.09; 0.28]	0.31	116	0.10 [-0.09; 0.28]	0.31	116	0.04 [-0.15; 0.23]	0.67	116	0.09 [-0.09; 0.28]	0.33
	mod1	118	0.02 [-0.17; 0.21]	0.81	118	-0.03 [-0.21; 0.16]	0.77	116	0.06 [-0.12; 0.25]	0.48	116	0.07 [-0.11; 0.25]	0.45	116	0.04 [-0.15; 0.22]	0.70	116	0.11 [-0.08; 0.29]	0.27

		HOMA2-IR (C-peptide)			HOMA2-IR (Insulin)			AUC (C-peptide/Glucose)			AUC (Insulin/Glucose)			oDI (C-peptide)			oDI (Insulin)		
**Metabolite**	**Model**	**n**	**Beta ± 95% CI**	**p-value**	**n**	**Beta ± 95% CI**	**p-value**	**n**	**Beta ± 95% CI**	**p-value**	**n**	**Beta ± 95% CI**	**p-value**	**n**	**Beta ± 95% CI**	**p-value**	**n**	**Beta ± 95% CI**	**p-value**
Cholesterol in XXL-VLDL	unadj.	118	0.04 [-0.15; 0.22]	0.69	118	0.01 [-0.17; 0.20]	0.88	116	0.07 [-0.12; 0.25]	0.47	116	0.07 [-0.11; 0.26]	0.44	116	-0.00 [-0.19; 0.18]	0.98	116	0.04 [-0.15; 0.22]	0.70
	mod1	118	0.02 [-0.16; 0.21]	0.80	118	-0.01 [-0.20; 0.18]	0.89	116	0.04 [-0.14; 0.23]	0.63	116	0.05 [-0.13; 0.24]	0.57	116	-0.00 [-0.19; 0.19]	0.98	116	0.06 [-0.14; 0.25]	0.57
Triglycerides in XXL-VLDL	unadj.	118	0.05 [-0.14; 0.23]	0.60	118	-0.00 [-0.19; 0.18]	0.99	116	0.12 [-0.07; 0.30]	0.21	116	0.12 [-0.07; 0.30]	0.21	116	0.06 [-0.12; 0.25]	0.51	116	0.12 [-0.06; 0.31]	0.19
	mod1	118	0.03 [-0.15; 0.22]	0.73	118	-0.03 [-0.21; 0.16]	0.75	116	0.08 [-0.09; 0.26]	0.35	116	0.09 [-0.09; 0.27]	0.34	116	0.05 [-0.13; 0.24]	0.57	116	0.13 [-0.06; 0.32]	0.17
Phospholipids in XL-VLDL	unadj.	118	0.02 [-0.17; 0.20]	0.85	118	0.01 [-0.17; 0.19]	0.91	116	0.04 [-0.15; 0.22]	0.70	116	0.03 [-0.15; 0.22]	0.71	116	0.00 [-0.18; 0.19]	>0.99	116	0.01 [-0.17; 0.20]	0.88
	mod1	118	0.01 [-0.18; 0.20]	0.96	118	-0.02 [-0.20; 0.17]	0.87	116	0.02 [-0.17; 0.20]	0.86	116	0.02 [-0.17; 0.20]	0.86	116	0.00 [-0.19; 0.19]	0.96	116	0.04 [-0.16; 0.23]	0.71
Cholesterol in XL-VLDL	unadj.	118	0.01 [-0.18; 0.19]	0.95	118	0.02 [-0.16; 0.20]	0.83	116	-0.02 [-0.21; 0.17]	0.83	116	-0.02 [-0.20; 0.17]	0.86	116	-0.04 [-0.23; 0.15]	0.67	116	-0.05 [-0.23; 0.14]	0.62
	mod1	118	-0.00 [-0.20; 0.19]	0.96	118	-0.00 [-0.19; 0.19]	0.98	116	-0.04 [-0.22; 0.15]	0.69	116	-0.03 [-0.22; 0.15]	0.74	116	-0.04 [-0.23; 0.16]	0.72	116	-0.03 [-0.22; 0.17]	0.78
Triglycerides in XL-VLDL	unadj.	118	0.04 [-0.14; 0.23]	0.64	118	0.02 [-0.17; 0.20]	0.85	116	0.09 [-0.09; 0.28]	0.33	116	0.09 [-0.10; 0.27]	0.36	116	0.02 [-0.17; 0.20]	0.84	116	0.05 [-0.13; 0.24]	0.59
	mod1	118	0.03 [-0.16; 0.22]	0.73	118	-0.01 [-0.20; 0.18]	0.94	116	0.07 [-0.11; 0.25]	0.45	116	0.07 [-0.12; 0.25]	0.47	116	0.02 [-0.17; 0.21]	0.83	116	0.07 [-0.12; 0.26]	0.47
Phospholipids in L-VLDL	unadj.	118	0.02 [-0.16; 0.20]	0.83	118	0.03 [-0.16; 0.21]	0.77	116	0.02 [-0.16; 0.21]	0.79	116	0.02 [-0.16; 0.21]	0.80	116	-0.02 [-0.20; 0.17]	0.86	116	-0.02 [-0.21; 0.17]	0.83
	mod1	118	0.01 [-0.18; 0.20]	0.92	118	0.00 [-0.18; 0.19]	0.96	116	0.01 [-0.17; 0.19]	0.91	116	0.01 [-0.18; 0.20]	0.91	116	-0.01 [-0.20; 0.18]	0.91	116	0.00 [-0.19; 0.20]	0.98
Cholesterol in L-VLDL	unadj.	118	0.01 [-0.18; 0.19]	0.95	118	0.03 [-0.15; 0.21]	0.75	116	-0.03 [-0.21; 0.16]	0.77	116	-0.02 [-0.21; 0.16]	0.80	116	-0.05 [-0.24; 0.13]	0.58	116	-0.07 [-0.26; 0.12]	0.46
	mod1	118	-0.00 [-0.19; 0.19]	0.98	118	0.01 [-0.18; 0.20]	0.91	116	-0.04 [-0.22; 0.14]	0.67	116	-0.04 [-0.22; 0.15]	0.71	116	-0.04 [-0.24; 0.15]	0.64	116	-0.05 [-0.24; 0.14]	0.61
Triglycerides in L-VLDL	unadj.	118	0.04 [-0.15; 0.22]	0.68	118	0.02 [-0.16; 0.20]	0.83	116	0.07 [-0.12; 0.25]	0.48	116	0.06 [-0.12; 0.25]	0.51	116	-0.00 [-0.19; 0.18]	0.98	116	0.02 [-0.16; 0.21]	0.81
	mod1	118	0.03 [-0.16; 0.22]	0.76	118	-0.00 [-0.19; 0.19]	0.98	116	0.05 [-0.13; 0.23]	0.58	116	0.05 [-0.14; 0.23]	0.61	116	0.00 [-0.19; 0.19]	0.98	116	0.04 [-0.15; 0.23]	0.65
Phospholipids in M-VLDL	unadj.	118	-0.02 [-0.21; 0.16]	0.81	118	0.03 [-0.15; 0.21]	0.74	116	-0.10 [-0.29; 0.08]	0.27	116	-0.10 [-0.28; 0.09]	0.29	116	-0.10 [-0.28; 0.09]	0.29	116	-0.15 [-0.33; 0.04]	0.11
	mod1	118	-0.03 [-0.22; 0.16]	0.74	118	0.01 [-0.17; 0.20]	0.89	116	-0.12 [-0.30; 0.06]	0.19	116	-0.11 [-0.30; 0.07]	0.22	116	-0.10 [-0.28; 0.09]	0.32	116	-0.13 [-0.32; 0.05]	0.16
Cholesterol in M-VLDL	unadj.	118	-0.05 [-0.23; 0.14]	0.61	118	0.02 [-0.16; 0.20]	0.82	116	-0.17 [-0.36; 0.01]	0.062	116	-0.16 [-0.35; 0.02]	0.082	116	-0.14 [-0.32; 0.05]	0.15	116	-0.20 [-0.38; -0.01]	0.034
	mod1	118	-0.05 [-0.24; 0.13]	0.57	118	0.01 [-0.18; 0.19]	0.92	116	-0.18 [-0.36; -0.01]	0.041	116	-0.17 [-0.35; 0.01]	0.061	116	-0.13 [-0.32; 0.05]	0.17	116	-0.19 [-0.37; -0.00]	0.049
Triglycerides in M-VLDL	unadj.	118	0.02 [-0.16; 0.20]	0.84	118	0.03 [-0.15; 0.21]	0.75	116	0.02 [-0.17; 0.20]	0.85	116	0.01 [-0.17; 0.20]	0.89	116	-0.03 [-0.22; 0.15]	0.72	116	-0.04 [-0.23; 0.14]	0.66
	mod1	118	0.01 [-0.18; 0.20]	0.92	118	0.01 [-0.18; 0.20]	0.94	116	0.00 [-0.18; 0.19]	0.96	116	0.00 [-0.19; 0.19]	0.99	116	-0.03 [-0.22; 0.16]	0.77	116	-0.02 [-0.21; 0.17]	0.84
Phospholipids in S-VLDL	unadj.	118	-0.04 [-0.22; 0.15]	0.68	118	0.02 [-0.16; 0.21]	0.79	116	-0.12 [-0.30; 0.07]	0.20	116	-0.11 [-0.30; 0.07]	0.23	116	-0.10 [-0.28; 0.09]	0.30	116	-0.15 [-0.34; 0.03]	0.098
	mod1	118	-0.05 [-0.24; 0.14]	0.62	118	0.01 [-0.18; 0.20]	0.94	116	-0.13 [-0.31; 0.05]	0.15	116	-0.13 [-0.31; 0.06]	0.18	116	-0.09 [-0.28; 0.10]	0.34	116	-0.14 [-0.33; 0.05]	0.15
Cholesterol in S-VLDL	unadj.	118	-0.05 [-0.23; 0.14]	0.62	118	0.02 [-0.16; 0.20]	0.84	116	-0.14 [-0.32; 0.05]	0.15	116	-0.13 [-0.31; 0.06]	0.17	116	-0.10 [-0.28; 0.09]	0.29	116	-0.16 [-0.34; 0.03]	0.090
	mod1	118	-0.05 [-0.24; 0.13]	0.57	118	0.00 [-0.18; 0.19]	0.97	116	-0.15 [-0.33; 0.03]	0.10	116	-0.14 [-0.32; 0.04]	0.13	116	-0.09 [-0.28; 0.10]	0.33	116	-0.14 [-0.33; 0.04]	0.13
Triglycerides in S-VLDL	unadj.	118	-0.01 [-0.19; 0.17]	0.92	118	0.01 [-0.17; 0.20]	0.89	116	-0.03 [-0.21; 0.16]	0.77	116	-0.03 [-0.21; 0.16]	0.76	116	-0.05 [-0.24; 0.14]	0.59	116	-0.07 [-0.25; 0.12]	0.49
	mod1	118	-0.02 [-0.21; 0.17]	0.84	118	-0.01 [-0.20; 0.18]	0.92	116	-0.04 [-0.23; 0.14]	0.65	116	-0.04 [-0.23; 0.15]	0.66	116	-0.04 [-0.24; 0.15]	0.65	116	-0.04 [-0.24; 0.15]	0.66
Phospholipids in XS-VLDL	unadj.	118	-0.07 [-0.25; 0.11]	0.46	118	-0.02 [-0.20; 0.17]	0.87	116	-0.15 [-0.33; 0.03]	0.11	116	-0.15 [-0.33; 0.04]	0.12	116	-0.09 [-0.28; 0.09]	0.33	116	-0.14 [-0.32; 0.05]	0.15
	mod1	118	-0.08 [-0.27; 0.10]	0.38	118	-0.04 [-0.22; 0.15]	0.69	116	-0.18 [-0.35; 0.00]	0.052	116	-0.17 [-0.35; 0.01]	0.066	116	-0.09 [-0.28; 0.10]	0.33	116	-0.13 [-0.31; 0.06]	0.19
Cholesterol in XS-VLDL	unadj.	118	-0.08 [-0.26; 0.10]	0.40	118	-0.02 [-0.20; 0.17]	0.85	116	-0.20 [-0.38; -0.01]	0.035	116	-0.19 [-0.37; -0.01]	0.042	116	-0.12 [-0.31; 0.06]	0.19	116	-0.18 [-0.36; 0.01]	0.060
	mod1	118	-0.09 [-0.28; 0.10]	0.34	118	-0.04 [-0.22; 0.15]	0.70	116	-0.22 [-0.39; -0.04]	0.015	116	-0.21 [-0.39; -0.03]	0.021	116	-0.13 [-0.31; 0.06]	0.18	116	-0.17 [-0.35; 0.02]	0.073
Triglycerides in XS-VLDL	unadj.	118	-0.04 [-0.22; 0.14]	0.67	118	-0.00 [-0.19; 0.18]	0.98	116	-0.07 [-0.26; 0.11]	0.43	116	-0.07 [-0.26; 0.11]	0.43	116	-0.05 [-0.24; 0.13]	0.56	116	-0.09 [-0.27; 0.10]	0.36
	mod1	118	-0.05 [-0.24; 0.13]	0.57	118	-0.03 [-0.22; 0.16]	0.77	116	-0.10 [-0.28; 0.08]	0.28	116	-0.10 [-0.28; 0.09]	0.29	116	-0.05 [-0.24; 0.14]	0.57	116	-0.07 [-0.26; 0.12]	0.46
Phospholipids in IDL	unadj.	118	-0.08 [-0.26; 0.10]	0.40	118	-0.01 [-0.19; 0.17]	0.92	116	-0.22 [-0.40; -0.03]	0.020	116	-0.21 [-0.39; -0.03]	0.023	116	-0.15 [-0.34; 0.03]	0.098	116	-0.22 [-0.40; -0.04]	0.020
	mod1	118	-0.09 [-0.27; 0.10]	0.36	118	-0.02 [-0.21; 0.16]	0.81	116	-0.23 [-0.40; -0.06]	0.0093 *	116	-0.22 [-0.40; -0.05]	0.013	116	-0.16 [-0.34; 0.03]	0.094	116	-0.21 [-0.39; -0.03]	0.023
Cholesterol in IDL	unadj.	118	-0.07 [-0.26; 0.11]	0.44	118	-0.01 [-0.19; 0.18]	0.95	116	-0.22 [-0.40; -0.04]	0.019	116	-0.21 [-0.39; -0.03]	0.023	116	-0.16 [-0.34; 0.03]	0.090	116	-0.22 [-0.40; -0.04]	0.018
	mod1	118	-0.08 [-0.26; 0.11]	0.41	118	-0.01 [-0.20; 0.17]	0.88	116	-0.23 [-0.40; -0.06]	0.010	116	-0.22 [-0.40; -0.05]	0.014	116	-0.16 [-0.34; 0.02]	0.088	116	-0.22 [-0.40; -0.03]	0.021
Triglycerides in IDL	unadj.	118	-0.05 [-0.23; 0.13]	0.59	118	-0.01 [-0.19; 0.18]	0.93	116	-0.10 [-0.29; 0.08]	0.27	116	-0.10 [-0.29; 0.08]	0.27	116	-0.07 [-0.25; 0.12]	0.46	116	-0.11 [-0.29; 0.08]	0.26
	mod1	118	-0.07 [-0.25; 0.12]	0.49	118	-0.03 [-0.22; 0.15]	0.72	116	-0.14 [-0.31; 0.04]	0.14	116	-0.13 [-0.31; 0.05]	0.15	116	-0.07 [-0.26; 0.11]	0.44	116	-0.10 [-0.29; 0.09]	0.32
Phospholipids in L-LDL	unadj.	118	-0.05 [-0.23; 0.14]	0.61	118	0.03 [-0.16; 0.21]	0.76	116	-0.17 [-0.36; 0.01]	0.063	116	-0.17 [-0.35; 0.02]	0.073	116	-0.15 [-0.33; 0.04]	0.12	116	-0.22 [-0.40; -0.04]	0.017
	mod1	118	-0.05 [-0.24; 0.13]	0.57	118	0.02 [-0.17; 0.20]	0.84	116	-0.18 [-0.36; -0.01]	0.043	116	-0.17 [-0.35; 0.00]	0.055	116	-0.14 [-0.33; 0.04]	0.13	116	-0.21 [-0.40; -0.03]	0.022
Cholesterol in L-LDL	unadj.	118	-0.05 [-0.24; 0.13]	0.56	118	0.03 [-0.16; 0.21]	0.78	116	-0.18 [-0.36; 0.01]	0.059	116	-0.17 [-0.35; 0.01]	0.071	116	-0.14 [-0.33; 0.04]	0.12	116	-0.22 [-0.40; -0.04]	0.018
	mod1	118	-0.06 [-0.25; 0.13]	0.53	118	0.02 [-0.17; 0.20]	0.87	116	-0.19 [-0.36; -0.01]	0.039	116	-0.18 [-0.36; 0.00]	0.051	116	-0.14 [-0.32; 0.04]	0.14	116	-0.21 [-0.39; -0.03]	0.025
Triglycerides in L-LDL	unadj.	118	-0.05 [-0.23; 0.14]	0.60	118	-0.01 [-0.19; 0.18]	0.95	116	-0.11 [-0.30; 0.07]	0.23	116	-0.11 [-0.30; 0.07]	0.23	116	-0.07 [-0.26; 0.11]	0.44	116	-0.11 [-0.29; 0.07]	0.24
	mod1	118	-0.06 [-0.25; 0.12]	0.50	118	-0.03 [-0.22; 0.16]	0.75	116	-0.14 [-0.32; 0.04]	0.12	116	-0.14 [-0.32; 0.04]	0.13	116	-0.08 [-0.26; 0.11]	0.41	116	-0.10 [-0.29; 0.09]	0.28
Phospholipids in M-LDL	unadj.	118	-0.03 [-0.22; 0.15]	0.74	118	0.04 [-0.14; 0.22]	0.68	116	-0.14 [-0.32; 0.05]	0.14	116	-0.13 [-0.32; 0.05]	0.16	116	-0.12 [-0.31; 0.06]	0.19	116	-0.19 [-0.38; -0.01]	0.037
	mod1	118	-0.04 [-0.22; 0.15]	0.70	118	0.03 [-0.16; 0.21]	0.76	116	-0.14 [-0.32; 0.03]	0.11	116	-0.14 [-0.32; 0.04]	0.13	116	-0.12 [-0.30; 0.07]	0.21	116	-0.18 [-0.37; 0.00]	0.051
Cholesterol in M-LDL	unadj.	118	-0.03 [-0.21; 0.15]	0.75	118	0.04 [-0.14; 0.22]	0.67	116	-0.13 [-0.32; 0.05]	0.15	116	-0.13 [-0.31; 0.06]	0.17	116	-0.12 [-0.30; 0.06]	0.20	116	-0.19 [-0.37; -0.01]	0.043
	mod1	118	-0.04 [-0.22; 0.15]	0.71	118	0.03 [-0.16; 0.21]	0.77	116	-0.14 [-0.32; 0.03]	0.11	116	-0.14 [-0.32; 0.04]	0.14	116	-0.11 [-0.30; 0.07]	0.23	116	-0.18 [-0.36; 0.01]	0.061
Triglycerides in M-LDL	unadj.	118	-0.04 [-0.23; 0.14]	0.64	118	-0.00 [-0.19; 0.18]	0.96	116	-0.09 [-0.28; 0.09]	0.31	116	-0.09 [-0.28; 0.09]	0.32	116	-0.06 [-0.25; 0.12]	0.50	116	-0.10 [-0.28; 0.09]	0.31
	mod1	118	-0.06 [-0.25; 0.13]	0.53	118	-0.03 [-0.22; 0.16]	0.75	116	-0.13 [-0.31; 0.05]	0.17	116	-0.12 [-0.30; 0.06]	0.19	116	-0.07 [-0.26; 0.12]	0.48	116	-0.09 [-0.27; 0.10]	0.37
Phospholipids in S-LDL	unadj.	118	-0.06 [-0.25; 0.12]	0.49	118	0.01 [-0.18; 0.19]	0.95	116	-0.18 [-0.36; 0.00]	0.054	116	-0.17 [-0.35; 0.02]	0.072	116	-0.13 [-0.31; 0.06]	0.18	116	-0.19 [-0.37; -0.00]	0.045
	mod1	118	-0.07 [-0.26; 0.12]	0.46	118	-0.01 [-0.19; 0.18]	0.95	116	-0.19 [-0.36; -0.01]	0.037	116	-0.17 [-0.35; 0.00]	0.056	116	-0.12 [-0.31; 0.07]	0.20	116	-0.17 [-0.36; 0.01]	0.065
Cholesterol in S-LDL	unadj.	118	-0.04 [-0.23; 0.14]	0.64	118	0.03 [-0.16; 0.21]	0.78	116	-0.16 [-0.34; 0.03]	0.096	116	-0.15 [-0.33; 0.04]	0.12	116	-0.12 [-0.31; 0.06]	0.19	116	-0.19 [-0.37; -0.01]	0.042
	mod1	118	-0.05 [-0.23; 0.14]	0.61	118	0.02 [-0.17; 0.20]	0.87	116	-0.16 [-0.34; 0.01]	0.070	116	-0.15 [-0.33; 0.03]	0.094	116	-0.12 [-0.30; 0.07]	0.21	116	-0.18 [-0.36; 0.01]	0.060
Triglycerides in S-LDL	unadj.	118	-0.03 [-0.21; 0.16]	0.79	118	-0.01 [-0.19; 0.18]	0.95	116	-0.05 [-0.24; 0.13]	0.57	116	-0.05 [-0.24; 0.13]	0.58	116	-0.05 [-0.23; 0.14]	0.61	116	-0.06 [-0.24; 0.13]	0.55
	mod1	118	-0.04 [-0.23; 0.15]	0.66	118	-0.04 [-0.22; 0.15]	0.71	116	-0.09 [-0.27; 0.10]	0.36	116	-0.08 [-0.27; 0.10]	0.39	116	-0.05 [-0.24; 0.14]	0.60	116	-0.04 [-0.23; 0.15]	0.67
Phospholipids in XL-HDL	unadj.	118	-0.11 [-0.29; 0.07]	0.23	118	-0.13 [-0.31; 0.06]	0.17	116	-0.21 [-0.39; -0.03]	0.024	116	-0.20 [-0.38; -0.02]	0.029	116	-0.11 [-0.30; 0.07]	0.22	116	-0.07 [-0.25; 0.12]	0.49

		HOMA2-IR (C-peptide)			HOMA2-IR (Insulin)			AUC (C-peptide/Glucose)			AUC (Insulin/Glucose)			oDI (C-peptide)			oDI (Insulin)		
Metabolite	Model	n	Beta ± 95% CI	p-value	n	Beta ± 95% CI	p-value	n	Beta ± 95% CI	p-value	n	Beta ± 95% CI	p-value	n	Beta ± 95% CI	p-value	n	Beta ± 95% CI	p-value
	mod1	118	-0.11 [-0.30; 0.07]	0.22	118	-0.13 [-0.31; 0.06]	0.17	116	-0.22 [-0.39; -0.05]	0.013	116	-0.21 [-0.39; -0.04]	0.018	116	-0.12 [-0.31; 0.06]	0.18	116	-0.08 [-0.26; 0.11]	0.42
Cholesterol in XL-HDL	unadj.	118	-0.12 [-0.30; 0.07]	0.21	118	-0.12 [-0.30; 0.06]	0.20	116	-0.23 [-0.41; -0.05]	0.013	116	-0.22 [-0.40; -0.04]	0.018	116	-0.12 [-0.31; 0.06]	0.19	116	-0.08 [-0.27; 0.10]	0.37
	mod1	118	-0.12 [-0.30; 0.06]	0.20	118	-0.12 [-0.30; 0.06]	0.20	116	-0.24 [-0.41; -0.07]	0.0072 *	116	-0.23 [-0.40; -0.05]	0.012	116	-0.13 [-0.31; 0.05]	0.16	116	-0.09 [-0.28; 0.09]	0.31
Triglycerides in XL-HDL	unadj.	118	-0.08 [-0.27; 0.10]	0.37	118	-0.07 [-0.25; 0.12]	0.48	116	-0.16 [-0.34; 0.02]	0.083	116	-0.16 [-0.34; 0.03]	0.091	116	-0.11 [-0.29; 0.08]	0.25	116	-0.10 [-0.28; 0.09]	0.29
	mod1	118	-0.10 [-0.29; 0.09]	0.29	118	-0.09 [-0.28; 0.09]	0.32	116	-0.19 [-0.37; -0.02]	0.032	116	-0.19 [-0.37; -0.01]	0.042	116	-0.11 [-0.30; 0.07]	0.23	116	-0.09 [-0.28; 0.10]	0.35
Phospholipids in L-HDL	unadj.	118	-0.07 [-0.25; 0.11]	0.45	118	-0.10 [-0.28; 0.08]	0.29	116	-0.12 [-0.30; 0.06]	0.20	116	-0.13 [-0.31; 0.06]	0.18	116	-0.10 [-0.28; 0.09]	0.30	116	-0.05 [-0.23; 0.14]	0.60
	mod1	118	-0.08 [-0.26; 0.11]	0.40	118	-0.10 [-0.29; 0.08]	0.27	116	-0.14 [-0.32; 0.03]	0.11	116	-0.15 [-0.33; 0.03]	0.11	116	-0.12 [-0.30; 0.07]	0.20	116	-0.07 [-0.26; 0.12]	0.47
Cholesterol in L-HDL	unadj.	118	-0.09 [-0.27; 0.10]	0.36	118	-0.10 [-0.28; 0.09]	0.29	116	-0.15 [-0.33; 0.03]	0.11	116	-0.15 [-0.34; 0.03]	0.10	116	-0.11 [-0.29; 0.08]	0.26	116	-0.07 [-0.26; 0.11]	0.45
	mod1	118	-0.09 [-0.28; 0.10]	0.34	118	-0.10 [-0.28; 0.09]	0.29	116	-0.17 [-0.34; 0.01]	0.062	116	-0.17 [-0.35; 0.01]	0.066	116	-0.12 [-0.31; 0.06]	0.19	116	-0.09 [-0.28; 0.10]	0.35
Triglycerides in L-HDL	unadj.	118	-0.09 [-0.27; 0.10]	0.36	118	-0.09 [-0.27; 0.09]	0.34	116	-0.15 [-0.34; 0.03]	0.097	116	-0.16 [-0.35; 0.02]	0.081	116	-0.12 [-0.31; 0.06]	0.19	116	-0.10 [-0.28; 0.09]	0.31
	mod1	118	-0.10 [-0.29; 0.08]	0.28	118	-0.12 [-0.30; 0.07]	0.22	116	-0.19 [-0.37; -0.02]	0.032	116	-0.20 [-0.37; -0.02]	0.030	116	-0.14 [-0.32; 0.05]	0.14	116	-0.09 [-0.28; 0.09]	0.33
Phospholipids in M-HDL	unadj.	118	0.04 [-0.15; 0.22]	0.69	118	-0.01 [-0.19; 0.18]	0.94	116	0.11 [-0.08; 0.29]	0.25	116	0.08 [-0.11; 0.26]	0.40	116	-0.01 [-0.20; 0.17]	0.88	116	0.02 [-0.17; 0.20]	0.84
	mod1	118	0.02 [-0.16; 0.21]	0.81	118	-0.02 [-0.21; 0.16]	0.81	116	0.07 [-0.11; 0.25]	0.47	116	0.04 [-0.14; 0.22]	0.65	116	-0.05 [-0.23; 0.14]	0.63	116	-0.00 [-0.19; 0.19]	0.96
Cholesterol in M-HDL	unadj.	118	0.02 [-0.16; 0.21]	0.81	118	-0.00 [-0.18; 0.18]	>0.99	116	0.06 [-0.13; 0.24]	0.52	116	0.04 [-0.15; 0.22]	0.69	116	-0.04 [-0.22; 0.15]	0.69	116	-0.03 [-0.21; 0.16]	0.78
	mod1	118	0.01 [-0.17; 0.20]	0.88	118	-0.00 [-0.19; 0.18]	0.96	116	0.03 [-0.15; 0.21]	0.71	116	0.01 [-0.17; 0.20]	0.87	116	-0.06 [-0.25; 0.12]	0.51	116	-0.05 [-0.24; 0.14]	0.59
Triglycerides in M-HDL	unadj.	118	-0.00 [-0.19; 0.18]	0.96	118	-0.01 [-0.19; 0.17]	0.92	116	0.01 [-0.18; 0.19]	0.95	116	-0.01 [-0.19; 0.18]	0.94	116	-0.04 [-0.23; 0.14]	0.65	116	-0.03 [-0.22; 0.15]	0.72
	mod1	118	-0.02 [-0.21; 0.16]	0.80	118	-0.04 [-0.23; 0.14]	0.64	116	-0.03 [-0.22; 0.15]	0.71	116	-0.04 [-0.23; 0.14]	0.64	116	-0.05 [-0.24; 0.14]	0.58	116	-0.02 [-0.21; 0.17]	0.82
Phospholipids in S-HDL	unadj.	118	0.09 [-0.09; 0.28]	0.32	118	0.07 [-0.11; 0.25]	0.44	116	0.18 [0.00; 0.37]	0.049	116	0.15 [-0.04; 0.33]	0.11	116	0.00 [-0.18; 0.19]	0.97	116	-0.00 [-0.19; 0.18]	0.97
	mod1	118	0.08 [-0.11; 0.26]	0.40	118	0.05 [-0.13; 0.24]	0.58	116	0.15 [-0.03; 0.32]	0.10	116	0.12 [-0.06; 0.29]	0.21	116	-0.02 [-0.20; 0.17]	0.87	116	-0.01 [-0.20; 0.17]	0.90
Cholesterol in S-HDL	unadj.	118	0.11 [-0.08; 0.29]	0.25	118	0.13 [-0.05; 0.31]	0.17	116	0.17 [-0.01; 0.35]	0.071	116	0.14 [-0.04; 0.32]	0.13	116	-0.00 [-0.19; 0.18]	0.97	116	-0.06 [-0.25; 0.12]	0.49
	mod1	118	0.10 [-0.08; 0.28]	0.28	118	0.12 [-0.06; 0.30]	0.19	116	0.16 [-0.02; 0.33]	0.080	116	0.13 [-0.05; 0.31]	0.15	116	-0.01 [-0.19; 0.18]	0.92	116	-0.07 [-0.25; 0.12]	0.48
Triglycerides in S-HDL	unadj.	118	0.01 [-0.18; 0.19]	0.94	118	0.02 [-0.17; 0.20]	0.85	116	0.01 [-0.17; 0.20]	0.89	116	0.01 [-0.18; 0.19]	0.93	116	-0.02 [-0.21; 0.16]	0.82	116	-0.03 [-0.22; 0.15]	0.74
	mod1	118	-0.01 [-0.20; 0.18]	0.94	118	-0.01 [-0.20; 0.18]	0.92	116	-0.01 [-0.19; 0.17]	0.91	116	-0.01 [-0.20; 0.17]	0.89	116	-0.02 [-0.21; 0.17]	0.83	116	-0.01 [-0.20; 0.18]	0.90
**Apolipoproteins**																			
Apolipoprotein B	unadj.	118	-0.05 [-0.23; 0.13]	0.58	118	0.02 [-0.17; 0.20]	0.85	116	-0.17 [-0.35; 0.02]	0.076	116	-0.16 [-0.34; 0.03]	0.090	116	-0.13 [-0.31; 0.06]	0.17	116	-0.19 [-0.37; -0.01]	0.039
	mod1	118	-0.06 [-0.25; 0.13]	0.53	118	0.00 [-0.18; 0.19]	0.97	116	-0.18 [-0.36; -0.00]	0.046	116	-0.17 [-0.35; 0.01]	0.061	116	-0.12 [-0.31; 0.06]	0.18	116	-0.18 [-0.37; 0.00]	0.055
Apolipoprotein A1	unadj.	118	-0.02 [-0.20; 0.16]	0.84	118	-0.05 [-0.23; 0.13]	0.58	116	-0.02 [-0.21; 0.16]	0.80	116	-0.05 [-0.23; 0.14]	0.63	116	-0.09 [-0.27; 0.10]	0.36	116	-0.05 [-0.23; 0.14]	0.60
	mod1	118	-0.03 [-0.22; 0.16]	0.74	118	-0.06 [-0.25; 0.12]	0.50	116	-0.06 [-0.24; 0.12]	0.51	116	-0.08 [-0.26; 0.10]	0.40	116	-0.11 [-0.30; 0.07]	0.23	116	-0.07 [-0.26; 0.12]	0.47
Ratio of apolipoprotein B to apolipoprotein A1	unadj.	118	-0.01 [-0.19; 0.17]	0.92	118	0.07 [-0.12; 0.25]	0.48	116	-0.12 [-0.31; 0.06]	0.19	116	-0.10 [-0.28; 0.09]	0.29	116	-0.10 [-0.28; 0.09]	0.29	116	-0.17 [-0.36; 0.01]	0.062
	mod1	118	-0.01 [-0.20; 0.18]	0.92	118	0.06 [-0.13; 0.25]	0.52	116	-0.12 [-0.30; 0.06]	0.19	116	-0.10 [-0.28; 0.09]	0.30	116	-0.09 [-0.27; 0.10]	0.37	116	-0.16 [-0.35; 0.03]	0.097
**Fatty acids**																			
Total fatty acids	unadj.	118	-0.02 [-0.21; 0.16]	0.82	118	0.00 [-0.18; 0.19]	0.97	116	-0.08 [-0.26; 0.11]	0.41	116	-0.08 [-0.27; 0.10]	0.38	116	-0.09 [-0.28; 0.09]	0.31	116	-0.11 [-0.29; 0.07]	0.24
	mod1	118	-0.04 [-0.23; 0.15]	0.69	118	-0.03 [-0.21; 0.16]	0.79	116	-0.11 [-0.30; 0.07]	0.21	116	-0.12 [-0.30; 0.07]	0.22	116	-0.10 [-0.29; 0.09]	0.28	116	-0.10 [-0.29; 0.09]	0.29
Degree of unsaturation	unadj.	118	-0.11 [-0.29; 0.07]	0.23	118	-0.03 [-0.21; 0.16]	0.77	116	-0.19 [-0.37; -0.01]	0.041	116	-0.21 [-0.40; -0.03]	0.021	116	-0.10 [-0.28; 0.09]	0.29	116	-0.18 [-0.37; -0.00]	0.049
	mod1	118	-0.11 [-0.29; 0.07]	0.24	118	-0.02 [-0.21; 0.16]	0.80	116	-0.19 [-0.36; -0.01]	0.035	116	-0.21 [-0.39; -0.04]	0.018	116	-0.10 [-0.28; 0.08]	0.29	116	-0.19 [-0.37; -0.00]	0.046
Omega-3 fatty acids	unadj.	118	-0.00 [-0.19; 0.18]	0.98	118	0.06 [-0.13; 0.24]	0.55	116	-0.03 [-0.22; 0.15]	0.73	116	-0.07 [-0.26; 0.12]	0.45	116	-0.05 [-0.24; 0.13]	0.58	116	-0.12 [-0.31; 0.06]	0.19
	mod1	118	-0.00 [-0.19; 0.18]	0.96	118	0.05 [-0.13; 0.23]	0.58	116	-0.04 [-0.21; 0.14]	0.69	116	-0.07 [-0.25; 0.11]	0.42	116	-0.05 [-0.24; 0.13]	0.59	116	-0.12 [-0.30; 0.07]	0.20
Omega-6 fatty acids	unadj.	118	-0.07 [-0.25; 0.11]	0.44	118	-0.03 [-0.22; 0.15]	0.73	116	-0.18 [-0.36; 0.01]	0.057	116	-0.18 [-0.36; 0.01]	0.058	116	-0.16 [-0.34; 0.03]	0.090	116	-0.18 [-0.36; 0.00]	0.052
	mod1	118	-0.08 [-0.27; 0.10]	0.38	118	-0.05 [-0.24; 0.13]	0.58	116	-0.20 [-0.38; -0.03]	0.024	116	-0.20 [-0.38; -0.02]	0.029	116	-0.16 [-0.35; 0.02]	0.081	116	-0.18 [-0.36; 0.01]	0.063
PUFA	unadj.	118	-0.06 [-0.25; 0.12]	0.50	118	-0.01 [-0.20; 0.17]	0.89	116	-0.16 [-0.35; 0.02]	0.081	116	-0.17 [-0.35; 0.01]	0.066	116	-0.15 [-0.33; 0.03]	0.11	116	-0.19 [-0.37; -0.01]	0.042
	mod1	118	-0.07 [-0.26; 0.11]	0.44	118	-0.03 [-0.21; 0.15]	0.75	116	-0.18 [-0.36; -0.01]	0.040	116	-0.19 [-0.37; -0.01]	0.035	116	-0.16 [-0.34; 0.03]	0.098	116	-0.18 [-0.37; 0.00]	0.051
MUFA	unadj.	118	-0.00 [-0.18; 0.18]	>0.99	118	0.02 [-0.17; 0.20]	0.86	116	-0.03 [-0.21; 0.16]	0.77	116	-0.03 [-0.21; 0.16]	0.78	116	-0.05 [-0.24; 0.13]	0.59	116	-0.06 [-0.24; 0.13]	0.53
	mod1	118	-0.02 [-0.21; 0.17]	0.83	118	-0.02 [-0.21; 0.17]	0.85	116	-0.07 [-0.25; 0.12]	0.46	116	-0.06 [-0.25; 0.12]	0.51	116	-0.06 [-0.25; 0.13]	0.54	116	-0.05 [-0.24; 0.15]	0.64
SFA	unadj.	118	-0.00 [-0.19; 0.18]	0.96	118	0.00 [-0.18; 0.19]	0.96	116	-0.05 [-0.23; 0.14]	0.63	116	-0.05 [-0.24; 0.14]	0.59	116	-0.07 [-0.26; 0.11]	0.43	116	-0.08 [-0.26; 0.11]	0.40
	mod1	118	-0.02 [-0.21; 0.17]	0.83	118	-0.02 [-0.21; 0.16]	0.81	116	-0.08 [-0.26; 0.10]	0.39	116	-0.08 [-0.26; 0.10]	0.39	116	-0.08 [-0.27; 0.11]	0.40	116	-0.07 [-0.26; 0.12]	0.48
Linoleic acid	unadj.	118	-0.08 [-0.27; 0.10]	0.37	118	-0.05 [-0.24; 0.13]	0.57	116	-0.18 [-0.36; 0.00]	0.054	116	-0.18 [-0.37; -0.00]	0.049	116	-0.15 [-0.34; 0.03]	0.100	116	-0.17 [-0.35; 0.02]	0.072
	mod1	118	-0.10 [-0.28; 0.09]	0.31	118	-0.07 [-0.26; 0.11]	0.43	116	-0.20 [-0.38; -0.03]	0.023	116	-0.20 [-0.38; -0.03]	0.024	116	-0.16 [-0.34; 0.03]	0.091	116	-0.16 [-0.35; 0.02]	0.088
Docosahexaenoic acid	unadj.	118	0.00 [-0.18; 0.19]	0.98	118	0.07 [-0.11; 0.25]	0.44	116	-0.04 [-0.22; 0.15]	0.70	116	-0.08 [-0.26; 0.11]	0.42	116	-0.08 [-0.27; 0.10]	0.37	116	-0.18 [-0.36; 0.01]	0.059
	mod1	118	0.00 [-0.18; 0.19]	0.97	118	0.07 [-0.11; 0.26]	0.43	116	-0.03 [-0.21; 0.14]	0.70	116	-0.07 [-0.25; 0.10]	0.41	116	-0.09 [-0.27; 0.10]	0.36	116	-0.18 [-0.36; 0.00]	0.054
Omega-3 FA / total FA	unadj.	118	-0.03 [-0.21; 0.16]	0.77	118	0.03 [-0.16; 0.21]	0.76	116	-0.01 [-0.20; 0.17]	0.88	116	-0.07 [-0.25; 0.12]	0.48	116	0.03 [-0.15; 0.22]	0.73	116	-0.05 [-0.24; 0.13]	0.59
	mod1	118	-0.02 [-0.20; 0.17]	0.84	118	0.04 [-0.14; 0.23]	0.65	116	0.00 [-0.18; 0.18]	0.98	116	-0.05 [-0.23; 0.13]	0.57	116	0.04 [-0.15; 0.22]	0.70	116	-0.06 [-0.24; 0.13]	0.54
Omega-6 FA / total FA	unadj.	118	-0.04 [-0.23; 0.14]	0.64	118	-0.03 [-0.21; 0.16]	0.78	116	-0.12 [-0.31; 0.06]	0.19	116	-0.11 [-0.29; 0.08]	0.26	116	-0.08 [-0.26; 0.11]	0.40	116	-0.09 [-0.28; 0.09]	0.33
	mod1	118	-0.03 [-0.22; 0.16]	0.75	118	0.00 [-0.19; 0.19]	>0.99	116	-0.10 [-0.28; 0.08]	0.29	116	-0.08 [-0.27; 0.10]	0.37	116	-0.08 [-0.26; 0.11]	0.42	116	-0.11 [-0.29; 0.08]	0.27
PUFA / total FA	unadj.	118	-0.05 [-0.24; 0.13]	0.58	118	-0.01 [-0.20; 0.17]	0.88	116	-0.12 [-0.30; 0.06]	0.20	116	-0.13 [-0.31; 0.06]	0.18	116	-0.06 [-0.25; 0.12]	0.51	116	-0.10 [-0.29; 0.08]	0.26
	mod1	118	-0.04 [-0.23; 0.15]	0.70	118	0.02 [-0.17; 0.20]	0.86	116	-0.09 [-0.27; 0.09]	0.32	116	-0.10 [-0.28; 0.08]	0.28	116	-0.06 [-0.25; 0.13]	0.54	116	-0.12 [-0.31; 0.07]	0.20
MUFA / total FA	unadj.	118	0.03 [-0.15; 0.22]	0.72	118	0.02 [-0.17; 0.20]	0.87	116	0.10 [-0.09; 0.28]	0.29	116	0.10 [-0.08; 0.29]	0.27	116	0.06 [-0.12; 0.25]	0.51	116	0.09 [-0.10; 0.27]	0.36
	mod1	118	0.01 [-0.18; 0.20]	0.91	118	-0.02 [-0.21; 0.17]	0.80	116	0.06 [-0.13; 0.24]	0.54	116	0.07 [-0.12; 0.25]	0.48	116	0.06 [-0.14; 0.25]	0.56	116	0.11 [-0.08; 0.30]	0.27
SFA / total FA	unadj.	118	0.05 [-0.13; 0.24]	0.56	118	0.00 [-0.18; 0.19]	0.96	116	0.09 [-0.09; 0.28]	0.33	116	0.09 [-0.09; 0.28]	0.31	116	0.03 [-0.15; 0.22]	0.74	116	0.08 [-0.11; 0.26]	0.40
	mod1	118	0.05 [-0.13; 0.24]	0.57	118	0.00 [-0.18; 0.18]	0.99	116	0.09 [-0.08; 0.27]	0.30	116	0.10 [-0.08; 0.27]	0.29	116	0.03 [-0.15; 0.22]	0.72	116	0.08 [-0.10; 0.27]	0.37

		HOMA2-IR (C-peptide)			HOMA2-IR (Insulin)			AUC (C-peptide/Glucose)			AUC (Insulin/Glucose)			oDI (C-peptide)			oDI (Insulin)		
**Metabolite**	**Model**	**n**	**Beta ± 95% CI**	**p-value**	**n**	**Beta ± 95% CI**	**p-value**	**n**	**Beta ± 95% CI**	**p-value**	**n**	**Beta ± 95% CI**	**p-value**	**n**	**Beta ± 95% CI**	**p-value**	**n**	**Beta ± 95% CI**	**p-value**
Linoleic acid / total FA	unadj.	118	-0.09 [-0.27; 0.09]	0.33	118	-0.07 [-0.26; 0.11]	0.42	116	-0.17 [-0.36; 0.01]	0.063	116	-0.17 [-0.35; 0.01]	0.071	116	-0.12 [-0.31; 0.06]	0.19	116	-0.13 [-0.31; 0.06]	0.18
	mod1	118	-0.08 [-0.27; 0.10]	0.38	118	-0.06 [-0.24; 0.12]	0.51	116	-0.15 [-0.33; 0.02]	0.083	116	-0.15 [-0.33; 0.03]	0.094	116	-0.12 [-0.30; 0.07]	0.21	116	-0.13 [-0.32; 0.05]	0.17
Docosahexaenoic acid / total FA	unadj.	118	-0.00 [-0.18; 0.18]	>0.99	118	0.05 [-0.14; 0.23]	0.61	116	0.00 [-0.18; 0.19]	>0.99	116	-0.04 [-0.23; 0.14]	0.66	116	0.00 [-0.18; 0.19]	0.96	116	-0.08 [-0.26; 0.11]	0.40
	mod1	118	0.01 [-0.17; 0.20]	0.89	118	0.07 [-0.11; 0.26]	0.44	116	0.03 [-0.16; 0.21]	0.78	116	-0.02 [-0.21; 0.16]	0.81	116	0.01 [-0.18; 0.19]	0.95	116	-0.10 [-0.29; 0.09]	0.31
PUFA / MUFA	unadj.	118	-0.04 [-0.23; 0.14]	0.64	118	-0.01 [-0.20; 0.17]	0.89	116	-0.12 [-0.30; 0.06]	0.20	116	-0.12 [-0.31; 0.06]	0.19	116	-0.07 [-0.25; 0.12]	0.48	116	-0.10 [-0.29; 0.08]	0.26
	mod1	118	-0.02 [-0.21; 0.17]	0.82	118	0.03 [-0.16; 0.22]	0.77	116	-0.08 [-0.26; 0.11]	0.41	116	-0.09 [-0.27; 0.10]	0.36	116	-0.06 [-0.25; 0.13]	0.54	116	-0.13 [-0.32; 0.07]	0.20
Omega-6 FA / omega-3 FA	unadj.	118	0.07 [-0.11; 0.25]	0.44	118	-0.01 [-0.20; 0.17]	0.88	116	0.06 [-0.12; 0.25]	0.52	116	0.09 [-0.10; 0.27]	0.35	116	-0.07 [-0.26; 0.11]	0.44	116	0.03 [-0.16; 0.21]	0.77
	mod1	118	0.07 [-0.11; 0.26]	0.43	118	-0.01 [-0.19; 0.17]	0.90	116	0.07 [-0.11; 0.24]	0.45	116	0.09 [-0.08; 0.27]	0.30	116	-0.07 [-0.25; 0.12]	0.46	116	0.03 [-0.15; 0.22]	0.74
**Phospholipids**																			
Phosphoglycerides	unadj.	117	-0.05 [-0.24; 0.13]	0.56	117	-0.03 [-0.21; 0.16]	0.75	115	-0.11 [-0.29; 0.08]	0.26	115	-0.12 [-0.31; 0.06]	0.18	115	-0.11 [-0.30; 0.08]	0.24	115	-0.14 [-0.32; 0.05]	0.14
	mod1	117	-0.07 [-0.26; 0.11]	0.44	117	-0.06 [-0.25; 0.13]	0.53	115	-0.15 [-0.33; 0.03]	0.096	115	-0.17 [-0.35; 0.02]	0.073	115	-0.13 [-0.31; 0.06]	0.18	115	-0.14 [-0.33; 0.05]	0.15
Ratio of triglycerides to phosphoglycerides	unadj.	117	0.03 [-0.15; 0.22]	0.71	117	-0.00 [-0.19; 0.18]	0.99	115	0.12 [-0.07; 0.30]	0.21	115	0.11 [-0.08; 0.29]	0.24	115	0.08 [-0.10; 0.27]	0.38	115	0.12 [-0.07; 0.30]	0.21
	mod1	117	0.03 [-0.16; 0.22]	0.78	117	-0.02 [-0.21; 0.17]	0.82	115	0.11 [-0.07; 0.29]	0.24	115	0.10 [-0.08; 0.29]	0.28	115	0.09 [-0.10; 0.28]	0.36	115	0.14 [-0.05; 0.33]	0.14
Total cholines	unadj.	118	-0.05 [-0.24; 0.13]	0.57	118	-0.01 [-0.20; 0.17]	0.88	116	-0.14 [-0.32; 0.04]	0.14	116	-0.14 [-0.33; 0.04]	0.12	116	-0.15 [-0.34; 0.03]	0.10	116	-0.18 [-0.36; 0.00]	0.052
	mod1	118	-0.07 [-0.26; 0.12]	0.45	118	-0.04 [-0.23; 0.14]	0.65	116	-0.18 [-0.36; -0.00]	0.046	116	-0.18 [-0.36; -0.00]	0.048	116	-0.17 [-0.35; 0.02]	0.079	116	-0.18 [-0.36; 0.01]	0.061
Phosphatidylcholines	unadj.	118	-0.05 [-0.24; 0.13]	0.57	118	-0.02 [-0.20; 0.16]	0.83	116	-0.13 [-0.31; 0.06]	0.18	116	-0.13 [-0.31; 0.05]	0.16	116	-0.15 [-0.33; 0.04]	0.12	116	-0.17 [-0.35; 0.02]	0.074
	mod1	118	-0.07 [-0.26; 0.11]	0.44	118	-0.05 [-0.24; 0.14]	0.59	116	-0.17 [-0.35; 0.01]	0.061	116	-0.17 [-0.35; 0.01]	0.064	116	-0.16 [-0.35; 0.03]	0.090	116	-0.16 [-0.35; 0.02]	0.086
Sphingomyelins	unadj.	118	-0.05 [-0.24; 0.13]	0.57	118	0.02 [-0.16; 0.20]	0.83	116	-0.22 [-0.40; -0.04]	0.017	116	-0.22 [-0.40; -0.04]	0.019	116	-0.19 [-0.37; -0.01]	0.042	116	-0.26 [-0.44; -0.08]	0.0042 *
	mod1	118	-0.06 [-0.24; 0.13]	0.52	118	0.01 [-0.17; 0.19]	0.93	116	-0.23 [-0.41; -0.06]	0.0084 *	116	-0.23 [-0.40; -0.05]	0.011	116	-0.19 [-0.37; -0.01]	0.041	116	-0.26 [-0.44; -0.08]	0.0054 *

Supplementary table 4 – Associations between maternal serum metabolites at 36 gestational weeks, and insulin secretion and insulin resistance in offspring at nine years age

Associations are given for regression coefficients (beta) with 95% confidence intervals (CI). Adjusted model 1 is adjusted for maternal pre-pregnancy BMI. P-values below 0.01 and 0.0038 are denoted with * and **, respectively.

		HOMA2-IR (C-peptide)			HOMA2-IR (Insulin)			AUC (C-peptide/Glucose)			AUC (Insulin/Glucose)			oDI (C-peptide)			oDI (Insulin)		
**Metabolite**	**Model**	**n**	**Beta ± 95% CI**	**p-value**	**n**	**Beta ± 95% CI**	**p-value**	**n**	**Beta ± 95% CI**	**p-value**	**n**	**Beta ± 95% CI**	**p-value**	**n**	**Beta ± 95% CI**	**p-value**	**n**	**Beta ± 95% CI**	**p-value**
**Lipids**																			
Total cholesterol	unadj.	111	-0.02 [-0.21; 0.17]	0.80	111	0.00 [-0.19; 0.19]	0.97	110	-0.08 [-0.27; 0.11]	0.41	110	-0.08 [-0.27; 0.11]	0.40	109	-0.07 [-0.26; 0.12]	0.48	109	-0.09 [-0.28; 0.10]	0.33
	mod1	111	-0.03 [-0.22; 0.17]	0.78	111	-0.01 [-0.20; 0.19]	0.95	110	-0.08 [-0.26; 0.10]	0.39	110	-0.08 [-0.27; 0.11]	0.41	109	-0.06 [-0.26; 0.13]	0.52	109	-0.08 [-0.28; 0.11]	0.41
Total cholesterol minus HDL cholesterol	unadj.	111	0.00 [-0.19; 0.19]	0.99	111	0.03 [-0.16; 0.22]	0.73	110	-0.05 [-0.25; 0.14]	0.57	110	-0.05 [-0.24; 0.14]	0.59	109	-0.06 [-0.25; 0.13]	0.55	109	-0.09 [-0.28; 0.10]	0.35
	mod1	111	0.00 [-0.19; 0.20]	0.97	111	0.03 [-0.16; 0.22]	0.76	110	-0.04 [-0.23; 0.14]	0.64	110	-0.04 [-0.23; 0.15]	0.67	109	-0.05 [-0.25; 0.15]	0.62	109	-0.08 [-0.27; 0.12]	0.45
Remnant cholesterol	unadj.	111	-0.00 [-0.19; 0.19]	0.98	111	0.02 [-0.17; 0.21]	0.84	110	-0.05 [-0.24; 0.14]	0.58	110	-0.05 [-0.24; 0.14]	0.61	109	-0.05 [-0.24; 0.14]	0.62	109	-0.07 [-0.26; 0.12]	0.47
	mod1	111	-0.00 [-0.20; 0.19]	0.98	111	0.01 [-0.18; 0.21]	0.89	110	-0.05 [-0.23; 0.14]	0.62	110	-0.04 [-0.23; 0.15]	0.67	109	-0.04 [-0.24; 0.16]	0.69	109	-0.05 [-0.25; 0.14]	0.58
VLDL cholesterol	unadj.	111	0.02 [-0.17; 0.21]	0.85	111	0.03 [-0.16; 0.22]	0.78	110	-0.01 [-0.20; 0.18]	0.89	110	-0.01 [-0.20; 0.18]	0.95	109	-0.03 [-0.22; 0.17]	0.79	109	-0.03 [-0.23; 0.16]	0.72
	mod1	111	0.02 [-0.17; 0.21]	0.84	111	0.02 [-0.17; 0.21]	0.82	110	-0.00 [-0.19; 0.18]	0.98	110	0.01 [-0.18; 0.20]	0.94	109	-0.02 [-0.21; 0.18]	0.87	109	-0.02 [-0.21; 0.18]	0.87
Clinical LDL cholesterol	unadj.	111	0.00 [-0.19; 0.19]	>0.99	111	0.04 [-0.15; 0.23]	0.64	110	-0.07 [-0.26; 0.12]	0.49	110	-0.07 [-0.26; 0.12]	0.48	109	-0.07 [-0.26; 0.12]	0.45	109	-0.12 [-0.31; 0.07]	0.21
	mod1	111	0.00 [-0.19; 0.20]	0.97	111	0.04 [-0.15; 0.24]	0.66	110	-0.06 [-0.24; 0.13]	0.55	110	-0.06 [-0.24; 0.13]	0.55	109	-0.06 [-0.26; 0.13]	0.51	109	-0.11 [-0.30; 0.09]	0.27
LDL cholesterol	unadj.	111	0.01 [-0.18; 0.20]	0.95	111	0.05 [-0.14; 0.24]	0.63	110	-0.05 [-0.25; 0.14]	0.57	110	-0.05 [-0.25; 0.14]	0.57	109	-0.07 [-0.26; 0.12]	0.48	109	-0.11 [-0.30; 0.08]	0.25
	mod1	111	0.01 [-0.18; 0.21]	0.92	111	0.05 [-0.15; 0.24]	0.64	110	-0.04 [-0.23; 0.14]	0.66	110	-0.04 [-0.23; 0.15]	0.68	109	-0.06 [-0.26; 0.14]	0.55	109	-0.10 [-0.29; 0.10]	0.33
HDL cholesterol	unadj.	111	-0.11 [-0.30; 0.08]	0.24	111	-0.13 [-0.32; 0.06]	0.16	110	-0.10 [-0.29; 0.09]	0.29	110	-0.12 [-0.31; 0.07]	0.22	109	-0.03 [-0.22; 0.16]	0.76	109	0.01 [-0.19; 0.20]	0.95
	mod1	111	-0.13 [-0.32; 0.06]	0.17	111	-0.16 [-0.34; 0.03]	0.10	110	-0.15 [-0.33; 0.03]	0.11	110	-0.16 [-0.34; 0.02]	0.083	109	-0.05 [-0.25; 0.14]	0.58	109	-0.01 [-0.21; 0.18]	0.90
Total triglycerides	unadj.	111	0.03 [-0.16; 0.22]	0.75	111	-0.01 [-0.20; 0.18]	0.94	110	0.05 [-0.14; 0.25]	0.57	110	0.05 [-0.14; 0.24]	0.58	109	0.04 [-0.15; 0.23]	0.70	109	0.07 [-0.12; 0.26]	0.47
	mod1	111	0.03 [-0.17; 0.22]	0.79	111	-0.02 [-0.21; 0.17]	0.83	110	0.05 [-0.13; 0.24]	0.57	110	0.05 [-0.13; 0.24]	0.57	109	0.04 [-0.15; 0.24]	0.68	109	0.08 [-0.11; 0.28]	0.39
Triglycerides in VLDL	unadj.	111	0.04 [-0.15; 0.23]	0.66	111	0.00 [-0.19; 0.19]	0.99	110	0.07 [-0.12; 0.26]	0.49	110	0.06 [-0.13; 0.25]	0.52	109	0.04 [-0.15; 0.23]	0.68	109	0.07 [-0.12; 0.26]	0.46
	mod1	111	0.04 [-0.15; 0.23]	0.69	111	-0.01 [-0.20; 0.18]	0.92	110	0.07 [-0.12; 0.25]	0.48	110	0.06 [-0.12; 0.25]	0.49	109	0.05 [-0.15; 0.24]	0.64	109	0.09 [-0.11; 0.28]	0.38
Triglycerides in LDL	unadj.	111	-0.02 [-0.21; 0.17]	0.87	111	-0.02 [-0.21; 0.17]	0.84	110	-0.01 [-0.20; 0.18]	0.94	110	0.00 [-0.19; 0.19]	0.98	109	0.01 [-0.18; 0.20]	0.90	109	0.03 [-0.17; 0.22]	0.79
	mod1	111	-0.02 [-0.22; 0.17]	0.81	111	-0.04 [-0.23; 0.15]	0.71	110	-0.02 [-0.20; 0.17]	0.85	110	-0.00 [-0.19; 0.18]	0.96	109	0.01 [-0.18; 0.21]	0.90	109	0.04 [-0.16; 0.23]	0.71
Triglycerides in HDL	unadj.	111	-0.03 [-0.22; 0.16]	0.76	111	-0.07 [-0.26; 0.12]	0.47	110	0.00 [-0.19; 0.19]	0.97	110	0.01 [-0.18; 0.20]	0.89	109	0.01 [-0.18; 0.20]	0.90	109	0.07 [-0.12; 0.26]	0.46
	mod1	111	-0.04 [-0.23; 0.15]	0.69	111	-0.09 [-0.27; 0.10]	0.37	110	-0.01 [-0.19; 0.17]	0.91	110	0.00 [-0.18; 0.19]	0.97	109	0.01 [-0.18; 0.20]	0.93	109	0.08 [-0.11; 0.27]	0.42
Total phospholipids in lipoprotein particles	unadj.	111	-0.06 [-0.25; 0.13]	0.53	111	-0.07 [-0.26; 0.12]	0.45	110	-0.07 [-0.26; 0.12]	0.46	110	-0.08 [-0.27; 0.11]	0.42	109	-0.04 [-0.23; 0.15]	0.69	109	-0.02 [-0.21; 0.18]	0.87
	mod1	111	-0.08 [-0.27; 0.11]	0.43	111	-0.10 [-0.29; 0.09]	0.30	110	-0.10 [-0.28; 0.08]	0.29	110	-0.10 [-0.28; 0.09]	0.29	109	-0.05 [-0.24; 0.15]	0.63	109	-0.01 [-0.21; 0.19]	0.92
Phospholipids in VLDL	unadj.	111	0.02 [-0.17; 0.21]	0.81	111	0.01 [-0.18; 0.20]	0.88	110	0.02 [-0.17; 0.21]	0.85	110	0.02 [-0.17; 0.21]	0.81	109	0.00 [-0.19; 0.19]	>0.99	109	0.01 [-0.18; 0.20]	0.93
	mod1	111	0.02 [-0.17; 0.22]	0.81	111	0.01 [-0.18; 0.20]	0.94	110	0.03 [-0.16; 0.21]	0.77	110	0.04 [-0.15; 0.22]	0.71	109	0.01 [-0.19; 0.21]	0.92	109	0.03 [-0.17; 0.22]	0.78
Phospholipids in LDL	unadj.	111	-0.00 [-0.19; 0.19]	>0.99	111	0.04 [-0.15; 0.23]	0.70	110	-0.06 [-0.25; 0.13]	0.55	110	-0.06 [-0.25; 0.13]	0.53	109	-0.07 [-0.26; 0.12]	0.49	109	-0.11 [-0.30; 0.08]	0.27
	mod1	111	0.00 [-0.19; 0.20]	0.98	111	0.04 [-0.16; 0.23]	0.71	110	-0.05 [-0.23; 0.14]	0.62	110	-0.05 [-0.24; 0.14]	0.62	109	-0.06 [-0.25; 0.14]	0.57	109	-0.09 [-0.29; 0.10]	0.35
Phospholipids in HDL	unadj.	111	-0.11 [-0.30; 0.08]	0.24	111	-0.16 [-0.34; 0.03]	0.10	110	-0.07 [-0.26; 0.12]	0.44	110	-0.09 [-0.28; 0.10]	0.36	109	-0.01 [-0.20; 0.19]	0.95	109	0.06 [-0.13; 0.25]	0.55
	mod1	111	-0.14 [-0.33; 0.05]	0.15	111	-0.19 [-0.38; -0.00]	0.047	110	-0.13 [-0.32; 0.05]	0.15	110	-0.14 [-0.33; 0.04]	0.12	109	-0.04 [-0.23; 0.16]	0.71	109	0.04 [-0.16; 0.24]	0.70
Total lipids in lipoprotein particles	unadj.	111	-0.02 [-0.21; 0.17]	0.87	111	-0.02 [-0.21; 0.17]	0.86	110	-0.04 [-0.23; 0.15]	0.68	110	-0.04 [-0.23; 0.15]	0.66	109	-0.03 [-0.22; 0.16]	0.73	109	-0.03 [-0.22; 0.16]	0.76
	mod1	111	-0.02 [-0.22; 0.17]	0.82	111	-0.03 [-0.23; 0.16]	0.73	110	-0.05 [-0.23; 0.14]	0.61	110	-0.05 [-0.23; 0.14]	0.63	109	-0.03 [-0.23; 0.16]	0.75	109	-0.02 [-0.21; 0.18]	0.87
Total lipids in VLDL	unadj.	111	0.03 [-0.16; 0.22]	0.74	111	0.01 [-0.18; 0.20]	0.89	110	0.03 [-0.16; 0.22]	0.73	110	0.03 [-0.16; 0.23]	0.72	109	0.01 [-0.18; 0.20]	0.90	109	0.03 [-0.16; 0.22]	0.78
	mod1	111	0.03 [-0.16; 0.23]	0.74	111	0.00 [-0.19; 0.20]	0.97	110	0.04 [-0.15; 0.22]	0.67	110	0.04 [-0.14; 0.23]	0.65	109	0.02 [-0.18; 0.22]	0.83	109	0.05 [-0.15; 0.24]	0.64
Total lipids in LDL	unadj.	111	0.00 [-0.19; 0.19]	0.97	111	0.04 [-0.15; 0.23]	0.68	110	-0.05 [-0.24; 0.14]	0.59	110	-0.05 [-0.24; 0.14]	0.59	109	-0.06 [-0.25; 0.13]	0.51	109	-0.10 [-0.29; 0.09]	0.29
	mod1	111	0.01 [-0.19; 0.20]	0.95	111	0.04 [-0.15; 0.23]	0.70	110	-0.04 [-0.23; 0.14]	0.66	110	-0.04 [-0.23; 0.15]	0.68	109	-0.05 [-0.25; 0.14]	0.59	109	-0.09 [-0.28; 0.11]	0.38
Total lipids in HDL	unadj.	111	-0.12 [-0.31; 0.07]	0.22	111	-0.15 [-0.34; 0.03]	0.11	110	-0.09 [-0.28; 0.10]	0.36	110	-0.10 [-0.29; 0.09]	0.28	109	-0.02 [-0.21; 0.17]	0.86	109	0.04 [-0.15; 0.23]	0.68
	mod1	111	-0.14 [-0.33; 0.05]	0.14	111	-0.18 [-0.37; 0.00]	0.053	110	-0.14 [-0.33; 0.04]	0.12	110	-0.15 [-0.34; 0.03]	0.098	109	-0.05 [-0.24; 0.15]	0.64	109	0.02 [-0.17; 0.22]	0.83
Total concentration of lipoprotein particles	unadj.	111	-0.09 [-0.28; 0.10]	0.34	111	-0.11 [-0.30; 0.07]	0.23	110	-0.06 [-0.25; 0.13]	0.50	110	-0.09 [-0.28; 0.10]	0.38	109	-0.05 [-0.24; 0.15]	0.64	109	-0.01 [-0.20; 0.19]	0.96
	mod1	111	-0.11 [-0.30; 0.08]	0.24	111	-0.14 [-0.33; 0.05]	0.14	110	-0.11 [-0.29; 0.07]	0.25	110	-0.12 [-0.31; 0.06]	0.19	109	-0.07 [-0.26; 0.13]	0.50	109	-0.01 [-0.21; 0.18]	0.89
Concentration of VLDL particles	unadj.	111	0.01 [-0.18; 0.20]	0.92	111	0.01 [-0.18; 0.20]	0.94	110	0.00 [-0.19; 0.19]	>0.99	110	0.01 [-0.18; 0.20]	0.94	109	-0.01 [-0.20; 0.18]	0.90	109	-0.01 [-0.20; 0.19]	0.95
	mod1	111	0.01 [-0.18; 0.21]	0.91	111	0.00 [-0.19; 0.19]	0.98	110	0.01 [-0.17; 0.20]	0.90	110	0.02 [-0.17; 0.21]	0.83	109	-0.00 [-0.20; 0.19]	0.99	109	0.01 [-0.19; 0.21]	0.90
Concentration of LDL particles	unadj.	111	0.01 [-0.18; 0.20]	0.93	111	0.04 [-0.15; 0.23]	0.68	110	-0.05 [-0.24; 0.14]	0.62	110	-0.04 [-0.24; 0.15]	0.64	109	-0.05 [-0.24; 0.14]	0.58	109	-0.09 [-0.28; 0.10]	0.37
	mod1	111	0.01 [-0.18; 0.20]	0.91	111	0.04 [-0.16; 0.23]	0.70	110	-0.04 [-0.22; 0.15]	0.69	110	-0.03 [-0.22; 0.16]	0.73	109	-0.04 [-0.24; 0.15]	0.66	109	-0.07 [-0.27; 0.13]	0.47
Concentration of HDL particles	unadj.	111	-0.09 [-0.28; 0.10]	0.33	111	-0.12 [-0.31; 0.07]	0.20	110	-0.05 [-0.24; 0.14]	0.58	110	-0.07 [-0.26; 0.12]	0.44	109	-0.03 [-0.22; 0.16]	0.73	109	0.02 [-0.18; 0.21]	0.87
	mod1	111	-0.11 [-0.30; 0.07]	0.23	111	-0.15 [-0.34; 0.04]	0.11	110	-0.10 [-0.28; 0.08]	0.29	110	-0.12 [-0.30; 0.07]	0.21	109	-0.06 [-0.25; 0.14]	0.56	109	0.00 [-0.19; 0.20]	0.98
Average diameter for VLDL particles	unadj.	111	0.02 [-0.17; 0.21]	0.83	111	-0.04 [-0.23; 0.15]	0.71	110	0.08 [-0.11; 0.27]	0.40	110	0.05 [-0.14; 0.24]	0.58	109	0.07 [-0.12; 0.26]	0.48	109	0.11 [-0.08; 0.30]	0.27
	mod1	111	0.02 [-0.17; 0.21]	0.86	111	-0.05 [-0.23; 0.14]	0.63	110	0.08 [-0.10; 0.26]	0.38	110	0.05 [-0.13; 0.24]	0.56	109	0.07 [-0.12; 0.26]	0.46	109	0.12 [-0.07; 0.31]	0.23
Average diameter for LDL particles	unadj.	111	-0.04 [-0.23; 0.15]	0.71	111	0.05 [-0.14; 0.24]	0.61	110	-0.06 [-0.25; 0.13]	0.51	110	-0.06 [-0.25; 0.13]	0.56	109	-0.03 [-0.23; 0.16]	0.72	109	-0.11 [-0.30; 0.08]	0.27
	mod1	111	-0.04 [-0.23; 0.15]	0.70	111	0.05 [-0.14; 0.23]	0.63	110	-0.06 [-0.25; 0.12]	0.48	110	-0.06 [-0.24; 0.13]	0.54	109	-0.03 [-0.23; 0.16]	0.73	109	-0.10 [-0.30; 0.09]	0.28
Average diameter for HDL particles	unadj.	111	-0.15 [-0.34; 0.04]	0.12	111	-0.18 [-0.37; 0.00]	0.053	110	-0.15 [-0.34; 0.04]	0.11	110	-0.15 [-0.34; 0.04]	0.13	109	0.03 [-0.16; 0.22]	0.75	109	0.09 [-0.10; 0.28]	0.35
	mod1	111	-0.16 [-0.35; 0.03]	0.090	111	-0.20 [-0.38; -0.01]	0.036	110	-0.18 [-0.36; -0.00]	0.044	110	-0.18 [-0.36; 0.01]	0.057	109	0.01 [-0.18; 0.21]	0.89	109	0.08 [-0.12; 0.27]	0.43

		HOMA2-IR (C-peptide)			HOMA2-IR (Insulin)			AUC (C-peptide/Glucose)			AUC (Insulin/Glucose)			oDI (C-peptide)			oDI (Insulin)		
Metabolite	Model	n	Beta ± 95% CI	p-value	n	Beta ± 95% CI	p-value	n	Beta ± 95% CI	p-value	n	Beta ± 95% CI	p-value	n	Beta ± 95% CI	p-value	n	Beta ± 95% CI	p-value
**Detailed lipoprotein contents**																			
Concentration of XXL-VLDL particles	unadj.	111	0.06 [-0.13; 0.25]	0.54	111	0.02 [-0.17; 0.21]	0.81	110	0.08 [-0.11; 0.27]	0.43	110	0.08 [-0.11; 0.27]	0.38	109	0.04 [-0.15; 0.23]	0.66	109	0.07 [-0.12; 0.26]	0.46
	mod1	111	0.05 [-0.14; 0.24]	0.61	111	0.00 [-0.18; 0.19]	0.96	110	0.06 [-0.12; 0.24]	0.51	110	0.07 [-0.11; 0.26]	0.43	109	0.04 [-0.15; 0.23]	0.68	109	0.08 [-0.11; 0.28]	0.41
Concentration of XL-VLDL particles	unadj.	111	0.04 [-0.15; 0.23]	0.65	111	0.01 [-0.18; 0.20]	0.92	110	0.07 [-0.12; 0.26]	0.48	110	0.07 [-0.13; 0.26]	0.50	109	0.04 [-0.15; 0.23]	0.66	109	0.07 [-0.12; 0.26]	0.48
	mod1	111	0.04 [-0.15; 0.23]	0.68	111	-0.00 [-0.19; 0.19]	0.98	110	0.07 [-0.12; 0.25]	0.46	110	0.07 [-0.12; 0.25]	0.47	109	0.05 [-0.15; 0.24]	0.62	109	0.08 [-0.11; 0.28]	0.39
Concentration of L-VLDL particles	unadj.	111	0.04 [-0.15; 0.23]	0.67	111	0.01 [-0.18; 0.20]	0.94	110	0.06 [-0.13; 0.25]	0.54	110	0.05 [-0.14; 0.25]	0.57	109	0.03 [-0.16; 0.22]	0.77	109	0.05 [-0.14; 0.25]	0.58
	mod1	111	0.04 [-0.15; 0.24]	0.66	111	0.00 [-0.19; 0.19]	>0.99	110	0.07 [-0.11; 0.25]	0.46	110	0.07 [-0.12; 0.25]	0.48	109	0.04 [-0.16; 0.23]	0.71	109	0.07 [-0.12; 0.27]	0.46
Concentration of M-VLDL particles	unadj.	111	0.03 [-0.16; 0.22]	0.76	111	0.03 [-0.16; 0.22]	0.73	110	-0.00 [-0.19; 0.19]	0.99	110	-0.00 [-0.19; 0.19]	>0.99	109	-0.03 [-0.22; 0.16]	0.76	109	-0.04 [-0.23; 0.15]	0.69
	mod1	111	0.04 [-0.16; 0.23]	0.72	111	0.03 [-0.16; 0.23]	0.74	110	0.02 [-0.17; 0.20]	0.86	110	0.02 [-0.17; 0.21]	0.84	109	-0.02 [-0.21; 0.18]	0.86	109	-0.02 [-0.22; 0.18]	0.85
Concentration of S-VLDL particles	unadj.	111	0.01 [-0.18; 0.20]	0.91	111	0.01 [-0.18; 0.20]	0.94	110	0.01 [-0.18; 0.20]	0.92	110	0.02 [-0.17; 0.21]	0.85	109	-0.02 [-0.21; 0.18]	0.88	109	-0.00 [-0.20; 0.19]	0.97
	mod1	111	0.02 [-0.18; 0.21]	0.86	111	0.01 [-0.18; 0.20]	0.93	110	0.03 [-0.15; 0.22]	0.74	110	0.04 [-0.15; 0.23]	0.66	109	-0.00 [-0.20; 0.19]	0.99	109	0.02 [-0.18; 0.21]	0.87
Concentration of XS-VLDL particles	unadj.	111	-0.02 [-0.21; 0.17]	0.80	111	-0.01 [-0.20; 0.18]	0.90	110	-0.04 [-0.23; 0.15]	0.69	110	-0.03 [-0.22; 0.16]	0.78	109	-0.02 [-0.21; 0.17]	0.86	109	-0.02 [-0.21; 0.17]	0.85
	mod1	111	-0.03 [-0.22; 0.16]	0.78	111	-0.02 [-0.21; 0.17]	0.82	110	-0.04 [-0.22; 0.15]	0.69	110	-0.02 [-0.21; 0.16]	0.80	109	-0.01 [-0.21; 0.18]	0.90	109	-0.01 [-0.20; 0.19]	0.96
Concentration of IDL particles	unadj.	111	-0.02 [-0.21; 0.17]	0.82	111	0.01 [-0.17; 0.20]	0.88	110	-0.09 [-0.28; 0.10]	0.37	110	-0.08 [-0.27; 0.11]	0.38	109	-0.07 [-0.26; 0.12]	0.48	109	-0.11 [-0.30; 0.08]	0.27
	mod1	111	-0.02 [-0.21; 0.17]	0.83	111	0.01 [-0.18; 0.20]	0.91	110	-0.08 [-0.26; 0.10]	0.39	110	-0.08 [-0.26; 0.11]	0.42	109	-0.06 [-0.26; 0.13]	0.53	109	-0.09 [-0.29; 0.10]	0.34
Concentration of L-LDL particles	unadj.	111	0.00 [-0.19; 0.19]	>0.99	111	0.04 [-0.15; 0.23]	0.70	110	-0.05 [-0.25; 0.14]	0.57	110	-0.05 [-0.24; 0.14]	0.60	109	-0.06 [-0.25; 0.14]	0.57	109	-0.09 [-0.28; 0.10]	0.35
	mod1	111	0.00 [-0.19; 0.20]	0.98	111	0.03 [-0.16; 0.23]	0.73	110	-0.05 [-0.23; 0.14]	0.63	110	-0.04 [-0.23; 0.15]	0.68	109	-0.05 [-0.24; 0.15]	0.64	109	-0.08 [-0.27; 0.12]	0.44
Concentration of M-LDL particles	unadj.	111	0.02 [-0.17; 0.21]	0.84	111	0.05 [-0.14; 0.24]	0.63	110	-0.03 [-0.22; 0.16]	0.73	110	-0.03 [-0.22; 0.16]	0.72	109	-0.05 [-0.24; 0.14]	0.61	109	-0.08 [-0.27; 0.11]	0.40
	mod1	111	0.02 [-0.17; 0.22]	0.82	111	0.04 [-0.15; 0.24]	0.66	110	-0.02 [-0.21; 0.16]	0.80	110	-0.02 [-0.21; 0.17]	0.82	109	-0.04 [-0.24; 0.16]	0.69	109	-0.07 [-0.26; 0.13]	0.51
Concentration of S-LDL particles	unadj.	111	0.02 [-0.17; 0.21]	0.87	111	0.04 [-0.15; 0.23]	0.68	110	-0.04 [-0.23; 0.15]	0.67	110	-0.04 [-0.23; 0.15]	0.70	109	-0.05 [-0.24; 0.14]	0.60	109	-0.08 [-0.27; 0.11]	0.42
	mod1	111	0.02 [-0.18; 0.21]	0.84	111	0.04 [-0.16; 0.23]	0.70	110	-0.03 [-0.21; 0.16]	0.77	110	-0.02 [-0.21; 0.17]	0.82	109	-0.04 [-0.24; 0.16]	0.69	109	-0.06 [-0.26; 0.14]	0.54
Concentration of XL-HDL particles	unadj.	111	-0.11 [-0.30; 0.07]	0.23	111	-0.15 [-0.34; 0.04]	0.12	110	-0.17 [-0.36; 0.02]	0.079	110	-0.17 [-0.36; 0.01]	0.069	109	-0.01 [-0.20; 0.18]	0.89	109	0.03 [-0.16; 0.22]	0.77
	mod1	111	-0.13 [-0.31; 0.06]	0.19	111	-0.16 [-0.35; 0.02]	0.085	110	-0.19 [-0.37; -0.02]	0.032	110	-0.20 [-0.38; -0.02]	0.031	109	-0.03 [-0.22; 0.17]	0.79	109	0.02 [-0.17; 0.21]	0.83
Concentration of L-HDL particles	unadj.	111	-0.13 [-0.32; 0.05]	0.16	111	-0.17 [-0.35; 0.02]	0.079	110	-0.16 [-0.35; 0.03]	0.091	110	-0.17 [-0.36; 0.02]	0.075	109	-0.03 [-0.22; 0.16]	0.75	109	0.02 [-0.17; 0.21]	0.84
	mod1	111	-0.15 [-0.34; 0.04]	0.11	111	-0.19 [-0.37; -0.00]	0.049	110	-0.20 [-0.38; -0.02]	0.027	110	-0.21 [-0.39; -0.03]	0.025	109	-0.05 [-0.25; 0.14]	0.60	109	0.00 [-0.19; 0.20]	0.97
Concentration of M-HDL particles	unadj.	111	-0.10 [-0.29; 0.09]	0.29	111	-0.13 [-0.32; 0.05]	0.16	110	-0.05 [-0.24; 0.14]	0.60	110	-0.06 [-0.25; 0.13]	0.54	109	-0.02 [-0.21; 0.17]	0.82	109	0.04 [-0.16; 0.23]	0.71
	mod1	111	-0.13 [-0.32; 0.06]	0.19	111	-0.16 [-0.35; 0.02]	0.086	110	-0.11 [-0.29; 0.08]	0.26	110	-0.11 [-0.30; 0.07]	0.24	109	-0.05 [-0.25; 0.14]	0.60	109	0.02 [-0.18; 0.21]	0.87
Concentration of S-HDL particles	unadj.	111	-0.00 [-0.19; 0.19]	0.97	111	-0.01 [-0.20; 0.18]	0.90	110	0.06 [-0.13; 0.25]	0.52	110	0.03 [-0.16; 0.22]	0.74	109	-0.03 [-0.22; 0.17]	0.78	109	-0.01 [-0.21; 0.18]	0.89
	mod1	111	-0.01 [-0.20; 0.18]	0.89	111	-0.03 [-0.22; 0.16]	0.76	110	0.04 [-0.14; 0.22]	0.64	110	0.02 [-0.17; 0.20]	0.87	109	-0.03 [-0.23; 0.16]	0.73	109	-0.01 [-0.21; 0.18]	0.90
Total lipids in XXL-VLDL	unadj.	111	0.06 [-0.13; 0.25]	0.53	111	0.02 [-0.17; 0.21]	0.82	110	0.08 [-0.11; 0.27]	0.42	110	0.08 [-0.11; 0.27]	0.38	109	0.05 [-0.14; 0.24]	0.63	109	0.08 [-0.12; 0.27]	0.44
	mod1	111	0.05 [-0.14; 0.24]	0.61	111	0.00 [-0.19; 0.19]	0.98	110	0.06 [-0.12; 0.24]	0.53	110	0.07 [-0.12; 0.25]	0.46	109	0.04 [-0.15; 0.24]	0.66	109	0.08 [-0.11; 0.28]	0.40
Total lipids in XL-VLDL	unadj.	111	0.05 [-0.14; 0.24]	0.62	111	0.01 [-0.18; 0.20]	0.92	110	0.07 [-0.12; 0.26]	0.46	110	0.07 [-0.12; 0.26]	0.50	109	0.05 [-0.15; 0.24]	0.64	109	0.07 [-0.12; 0.26]	0.46
	mod1	111	0.04 [-0.15; 0.24]	0.65	111	-0.00 [-0.19; 0.19]	0.98	110	0.07 [-0.11; 0.25]	0.46	110	0.07 [-0.12; 0.25]	0.48	109	0.05 [-0.14; 0.24]	0.61	109	0.09 [-0.11; 0.28]	0.38
Total lipids in L-VLDL	unadj.	111	0.05 [-0.14; 0.24]	0.63	111	0.01 [-0.18; 0.20]	0.92	110	0.06 [-0.13; 0.25]	0.53	110	0.05 [-0.14; 0.24]	0.58	109	0.03 [-0.16; 0.22]	0.76	109	0.06 [-0.14; 0.25]	0.57
	mod1	111	0.05 [-0.15; 0.24]	0.63	111	0.00 [-0.19; 0.19]	0.98	110	0.07 [-0.11; 0.25]	0.46	110	0.06 [-0.12; 0.25]	0.50	109	0.04 [-0.16; 0.23]	0.70	109	0.07 [-0.12; 0.27]	0.46
Total lipids in M-VLDL	unadj.	111	0.03 [-0.16; 0.22]	0.75	111	0.02 [-0.17; 0.21]	0.80	110	0.01 [-0.18; 0.20]	0.93	110	0.01 [-0.18; 0.20]	0.95	109	-0.02 [-0.21; 0.17]	0.86	109	-0.02 [-0.21; 0.17]	0.86
	mod1	111	0.04 [-0.16; 0.23]	0.72	111	0.02 [-0.17; 0.21]	0.83	110	0.03 [-0.16; 0.21]	0.79	110	0.02 [-0.16; 0.21]	0.80	109	-0.00 [-0.20; 0.19]	0.96	109	0.00 [-0.20; 0.20]	0.97
Total lipids in S-VLDL	unadj.	111	0.01 [-0.18; 0.20]	0.92	111	0.01 [-0.18; 0.20]	0.92	110	0.00 [-0.19; 0.19]	>0.99	110	0.01 [-0.18; 0.20]	0.93	109	-0.02 [-0.21; 0.18]	0.87	109	-0.01 [-0.20; 0.18]	0.91
	mod1	111	0.02 [-0.18; 0.21]	0.88	111	0.01 [-0.18; 0.20]	0.92	110	0.02 [-0.16; 0.21]	0.83	110	0.03 [-0.16; 0.22]	0.75	109	-0.00 [-0.20; 0.19]	0.98	109	0.01 [-0.19; 0.21]	0.93
Total lipids in XS-VLDL	unadj.	111	-0.03 [-0.22; 0.16]	0.77	111	-0.01 [-0.20; 0.17]	0.88	110	-0.04 [-0.23; 0.15]	0.68	110	-0.03 [-0.22; 0.16]	0.77	109	-0.01 [-0.21; 0.18]	0.89	109	-0.01 [-0.21; 0.18]	0.88
	mod1	111	-0.03 [-0.22; 0.16]	0.74	111	-0.03 [-0.22; 0.16]	0.79	110	-0.04 [-0.23; 0.14]	0.65	110	-0.03 [-0.21; 0.16]	0.77	109	-0.01 [-0.20; 0.18]	0.92	109	-0.00 [-0.20; 0.19]	0.98
Total lipids in IDL	unadj.	111	-0.04 [-0.23; 0.15]	0.70	111	-0.00 [-0.19; 0.19]	0.98	110	-0.10 [-0.29; 0.09]	0.31	110	-0.10 [-0.29; 0.09]	0.31	109	-0.06 [-0.26; 0.13]	0.50	109	-0.10 [-0.29; 0.09]	0.32
	mod1	111	-0.04 [-0.23; 0.15]	0.68	111	-0.01 [-0.20; 0.18]	0.91	110	-0.10 [-0.28; 0.08]	0.28	110	-0.10 [-0.28; 0.09]	0.30	109	-0.06 [-0.26; 0.13]	0.53	109	-0.09 [-0.28; 0.11]	0.38
Total lipids in L-LDL	unadj.	111	-0.01 [-0.20; 0.18]	0.95	111	0.03 [-0.16; 0.22]	0.72	110	-0.06 [-0.25; 0.13]	0.51	110	-0.06 [-0.25; 0.13]	0.52	109	-0.07 [-0.26; 0.12]	0.48	109	-0.11 [-0.30; 0.08]	0.27
	mod1	111	-0.00 [-0.20; 0.19]	0.97	111	0.03 [-0.16; 0.22]	0.75	110	-0.05 [-0.24; 0.13]	0.57	110	-0.05 [-0.24; 0.14]	0.59	109	-0.06 [-0.26; 0.14]	0.55	109	-0.09 [-0.29; 0.10]	0.35
Total lipids in M-LDL	unadj.	111	0.02 [-0.17; 0.21]	0.84	111	0.05 [-0.14; 0.24]	0.60	110	-0.03 [-0.22; 0.16]	0.73	110	-0.03 [-0.22; 0.16]	0.72	109	-0.06 [-0.25; 0.14]	0.56	109	-0.09 [-0.28; 0.10]	0.34
	mod1	111	0.02 [-0.17; 0.22]	0.81	111	0.05 [-0.14; 0.24]	0.61	110	-0.02 [-0.20; 0.17]	0.84	110	-0.02 [-0.21; 0.17]	0.85	109	-0.05 [-0.24; 0.15]	0.65	109	-0.08 [-0.27; 0.12]	0.45
Total lipids in S-LDL	unadj.	111	0.01 [-0.18; 0.20]	0.89	111	0.04 [-0.15; 0.23]	0.65	110	-0.04 [-0.23; 0.15]	0.65	110	-0.04 [-0.23; 0.15]	0.66	109	-0.05 [-0.25; 0.14]	0.57	109	-0.09 [-0.28; 0.10]	0.35
	mod1	111	0.02 [-0.18; 0.21]	0.85	111	0.04 [-0.15; 0.24]	0.65	110	-0.03 [-0.21; 0.16]	0.76	110	-0.03 [-0.21; 0.16]	0.79	109	-0.04 [-0.24; 0.15]	0.67	109	-0.07 [-0.27; 0.13]	0.47
Total lipids in XL-HDL	unadj.	111	-0.12 [-0.31; 0.07]	0.22	111	-0.15 [-0.34; 0.04]	0.11	110	-0.17 [-0.36; 0.02]	0.074	110	-0.18 [-0.37; 0.01]	0.063	109	-0.01 [-0.20; 0.19]	0.96	109	0.03 [-0.16; 0.23]	0.72
	mod1	111	-0.13 [-0.32; 0.06]	0.17	111	-0.17 [-0.35; 0.02]	0.078	110	-0.20 [-0.38; -0.02]	0.026	110	-0.21 [-0.39; -0.03]	0.025	109	-0.02 [-0.21; 0.17]	0.83	109	0.02 [-0.17; 0.22]	0.81
Total lipids in L-HDL	unadj.	111	-0.13 [-0.32; 0.06]	0.18	111	-0.16 [-0.35; 0.03]	0.093	110	-0.15 [-0.34; 0.04]	0.12	110	-0.16 [-0.34; 0.03]	0.10	109	-0.03 [-0.22; 0.17]	0.79	109	0.02 [-0.17; 0.21]	0.82
	mod1	111	-0.15 [-0.34; 0.04]	0.12	111	-0.18 [-0.37; 0.01]	0.057	110	-0.19 [-0.37; -0.01]	0.037	110	-0.20 [-0.38; -0.02]	0.033	109	-0.05 [-0.24; 0.15]	0.62	109	0.00 [-0.19; 0.20]	0.97
Total lipids in M-HDL	unadj.	111	-0.09 [-0.28; 0.10]	0.37	111	-0.12 [-0.31; 0.07]	0.22	110	-0.02 [-0.21; 0.17]	0.81	110	-0.03 [-0.23; 0.16]	0.72	109	-0.01 [-0.20; 0.18]	0.91	109	0.04 [-0.15; 0.23]	0.66
	mod1	111	-0.11 [-0.30; 0.08]	0.24	111	-0.15 [-0.34; 0.04]	0.12	110	-0.08 [-0.26; 0.10]	0.39	110	-0.09 [-0.27; 0.10]	0.36	109	-0.04 [-0.24; 0.15]	0.68	109	0.02 [-0.17; 0.22]	0.83
Total lipids in S-HDL	unadj.	111	-0.01 [-0.20; 0.18]	0.90	111	-0.04 [-0.23; 0.15]	0.71	110	0.09 [-0.10; 0.28]	0.35	110	0.07 [-0.12; 0.26]	0.49	109	0.00 [-0.19; 0.20]	0.97	109	0.04 [-0.15; 0.23]	0.67
	mod1	111	-0.03 [-0.22; 0.16]	0.76	111	-0.06 [-0.25; 0.13]	0.51	110	0.05 [-0.13; 0.24]	0.56	110	0.04 [-0.15; 0.22]	0.70	109	-0.01 [-0.20; 0.18]	0.90	109	0.04 [-0.16; 0.23]	0.71
Phospholipids in XXL-VLDL	unadj.	111	0.05 [-0.13; 0.24]	0.57	111	0.02 [-0.17; 0.21]	0.81	110	0.07 [-0.12; 0.26]	0.46	110	0.09 [-0.11; 0.28]	0.38	109	0.04 [-0.15; 0.23]	0.70	109	0.07 [-0.12; 0.26]	0.49
	mod1	111	0.05 [-0.15; 0.24]	0.64	111	0.00 [-0.18; 0.19]	0.96	110	0.06 [-0.13; 0.24]	0.54	110	0.07 [-0.11; 0.26]	0.43	109	0.03 [-0.16; 0.23]	0.72	109	0.08 [-0.12; 0.27]	0.44

		HOMA2-IR (C-peptide)			HOMA2-IR (Insulin)			AUC (C-peptide/Glucose)			AUC (Insulin/Glucose)			oDI (C-peptide)			oDI (Insulin)		
Metabolite	Model	n	Beta ± 95% CI	p-value	n	Beta ± 95% CI	p-value	n	Beta ± 95% CI	p-value	n	Beta ± 95% CI	p-value	n	Beta ± 95% CI	p-value	n	Beta ± 95% CI	p-value
Cholesterol in XXL-VLDL	unadj.	111	0.05 [-0.14; 0.24]	0.62	111	0.02 [-0.17; 0.21]	0.82	110	0.06 [-0.13; 0.25]	0.54	110	0.07 [-0.12; 0.26]	0.45	109	0.02 [-0.17; 0.21]	0.84	109	0.05 [-0.15; 0.24]	0.63
	mod1	111	0.04 [-0.15; 0.23]	0.66	111	0.01 [-0.18; 0.20]	0.93	110	0.05 [-0.13; 0.24]	0.57	110	0.07 [-0.12; 0.26]	0.46	109	0.02 [-0.17; 0.21]	0.83	109	0.06 [-0.14; 0.25]	0.55
Triglycerides in XXL-VLDL	unadj.	111	0.06 [-0.13; 0.25]	0.51	111	0.02 [-0.17; 0.21]	0.82	110	0.08 [-0.11; 0.27]	0.39	110	0.09 [-0.10; 0.28]	0.37	109	0.06 [-0.13; 0.25]	0.55	109	0.09 [-0.11; 0.28]	0.38
	mod1	111	0.05 [-0.14; 0.24]	0.60	111	0.00 [-0.19; 0.19]	>0.99	110	0.06 [-0.12; 0.24]	0.52	110	0.07 [-0.12; 0.25]	0.48	109	0.05 [-0.14; 0.24]	0.60	109	0.09 [-0.10; 0.29]	0.35
Phospholipids in XL-VLDL	unadj.	111	0.04 [-0.15; 0.23]	0.68	111	0.00 [-0.18; 0.19]	0.96	110	0.06 [-0.13; 0.25]	0.51	110	0.06 [-0.13; 0.25]	0.53	109	0.05 [-0.14; 0.24]	0.63	109	0.07 [-0.12; 0.26]	0.45
	mod1	111	0.04 [-0.16; 0.23]	0.72	111	-0.01 [-0.20; 0.18]	0.94	110	0.06 [-0.12; 0.25]	0.51	110	0.06 [-0.12; 0.25]	0.51	109	0.05 [-0.14; 0.25]	0.60	109	0.09 [-0.11; 0.28]	0.37
Cholesterol in XL-VLDL	unadj.	111	0.05 [-0.14; 0.23]	0.64	111	0.03 [-0.16; 0.22]	0.79	110	0.04 [-0.15; 0.23]	0.69	110	0.04 [-0.15; 0.23]	0.68	109	0.01 [-0.18; 0.20]	0.91	109	0.02 [-0.17; 0.21]	0.82
	mod1	111	0.05 [-0.15; 0.24]	0.63	111	0.02 [-0.17; 0.21]	0.84	110	0.05 [-0.14; 0.23]	0.61	110	0.05 [-0.14; 0.24]	0.59	109	0.02 [-0.18; 0.22]	0.84	109	0.04 [-0.16; 0.24]	0.68
Triglycerides in XL-VLDL	unadj.	111	0.05 [-0.14; 0.24]	0.60	111	0.00 [-0.19; 0.19]	0.96	110	0.08 [-0.11; 0.28]	0.38	110	0.08 [-0.11; 0.27]	0.43	109	0.06 [-0.13; 0.25]	0.55	109	0.09 [-0.10; 0.28]	0.35
	mod1	111	0.04 [-0.16; 0.24]	0.65	111	-0.01 [-0.20; 0.18]	0.92	110	0.08 [-0.10; 0.26]	0.40	110	0.07 [-0.11; 0.26]	0.44	109	0.06 [-0.13; 0.25]	0.54	109	0.10 [-0.09; 0.30]	0.30
Phospholipids in L-VLDL	unadj.	111	0.04 [-0.15; 0.23]	0.66	111	0.01 [-0.18; 0.20]	0.90	110	0.06 [-0.13; 0.25]	0.55	110	0.06 [-0.13; 0.25]	0.56	109	0.02 [-0.17; 0.22]	0.80	109	0.05 [-0.14; 0.24]	0.62
	mod1	111	0.05 [-0.15; 0.24]	0.64	111	0.01 [-0.18; 0.20]	0.94	110	0.07 [-0.11; 0.25]	0.46	110	0.07 [-0.12; 0.26]	0.46	109	0.03 [-0.16; 0.23]	0.72	109	0.07 [-0.13; 0.26]	0.50
Cholesterol in L-VLDL	unadj.	111	0.04 [-0.15; 0.23]	0.66	111	0.02 [-0.17; 0.21]	0.81	110	0.03 [-0.16; 0.23]	0.72	110	0.03 [-0.16; 0.23]	0.72	109	-0.00 [-0.19; 0.19]	>0.99	109	0.01 [-0.18; 0.21]	0.89
	mod1	111	0.05 [-0.15; 0.24]	0.62	111	0.02 [-0.17; 0.21]	0.82	110	0.05 [-0.13; 0.24]	0.56	110	0.06 [-0.13; 0.24]	0.56	109	0.01 [-0.18; 0.21]	0.89	109	0.03 [-0.16; 0.23]	0.73
Triglycerides in L-VLDL	unadj.	111	0.05 [-0.14; 0.24]	0.62	111	0.00 [-0.19; 0.19]	>0.99	110	0.07 [-0.12; 0.26]	0.44	110	0.06 [-0.13; 0.25]	0.52	109	0.05 [-0.14; 0.24]	0.62	109	0.08 [-0.11; 0.27]	0.41
	mod1	111	0.05 [-0.15; 0.24]	0.64	111	-0.01 [-0.20; 0.18]	0.91	110	0.07 [-0.11; 0.26]	0.42	110	0.06 [-0.12; 0.25]	0.49	109	0.05 [-0.14; 0.25]	0.59	109	0.09 [-0.10; 0.29]	0.34
Phospholipids in M-VLDL	unadj.	111	0.03 [-0.16; 0.22]	0.79	111	0.03 [-0.16; 0.22]	0.73	110	-0.01 [-0.20; 0.18]	0.93	110	-0.01 [-0.20; 0.18]	0.94	109	-0.03 [-0.22; 0.16]	0.74	109	-0.04 [-0.24; 0.15]	0.64
	mod1	111	0.03 [-0.16; 0.23]	0.75	111	0.03 [-0.16; 0.23]	0.74	110	0.01 [-0.18; 0.19]	0.93	110	0.01 [-0.18; 0.20]	0.91	109	-0.02 [-0.22; 0.18]	0.84	109	-0.03 [-0.22; 0.17]	0.80
Cholesterol in M-VLDL	unadj.	111	0.02 [-0.17; 0.21]	0.87	111	0.05 [-0.14; 0.24]	0.59	110	-0.06 [-0.25; 0.13]	0.55	110	-0.05 [-0.24; 0.14]	0.58	109	-0.07 [-0.26; 0.12]	0.48	109	-0.11 [-0.30; 0.08]	0.26
	mod1	111	0.02 [-0.17; 0.22]	0.82	111	0.05 [-0.14; 0.25]	0.58	110	-0.04 [-0.23; 0.14]	0.65	110	-0.04 [-0.22; 0.15]	0.71	109	-0.06 [-0.25; 0.14]	0.56	109	-0.09 [-0.29; 0.10]	0.35
Triglycerides in M-VLDL	unadj.	111	0.03 [-0.16; 0.22]	0.72	111	-0.00 [-0.19; 0.19]	>0.99	110	0.05 [-0.14; 0.24]	0.59	110	0.04 [-0.15; 0.23]	0.65	109	0.02 [-0.17; 0.21]	0.83	109	0.05 [-0.14; 0.24]	0.62
	mod1	111	0.04 [-0.16; 0.23]	0.70	111	-0.01 [-0.20; 0.19]	0.96	110	0.07 [-0.12; 0.25]	0.48	110	0.06 [-0.13; 0.25]	0.53	109	0.03 [-0.16; 0.23]	0.75	109	0.07 [-0.13; 0.26]	0.50
Phospholipids in S-VLDL	unadj.	111	0.01 [-0.18; 0.20]	0.92	111	0.02 [-0.17; 0.21]	0.83	110	-0.01 [-0.20; 0.18]	0.91	110	-0.00 [-0.19; 0.19]	0.98	109	-0.03 [-0.22; 0.16]	0.75	109	-0.04 [-0.23; 0.15]	0.70
	mod1	111	0.02 [-0.18; 0.21]	0.87	111	0.02 [-0.17; 0.21]	0.82	110	0.01 [-0.18; 0.20]	0.91	110	0.02 [-0.17; 0.21]	0.83	109	-0.02 [-0.21; 0.18]	0.86	109	-0.02 [-0.21; 0.18]	0.86
Cholesterol in S-VLDL	unadj.	111	0.01 [-0.18; 0.20]	0.95	111	0.03 [-0.16; 0.22]	0.78	110	-0.02 [-0.21; 0.17]	0.84	110	-0.01 [-0.20; 0.18]	0.93	109	-0.03 [-0.22; 0.16]	0.75	109	-0.04 [-0.24; 0.15]	0.64
	mod1	111	0.01 [-0.18; 0.21]	0.91	111	0.03 [-0.16; 0.22]	0.78	110	-0.00 [-0.19; 0.18]	0.98	110	0.01 [-0.18; 0.20]	0.90	109	-0.02 [-0.21; 0.18]	0.85	109	-0.03 [-0.22; 0.17]	0.79
Triglycerides in S-VLDL	unadj.	111	0.01 [-0.18; 0.20]	0.90	111	-0.02 [-0.21; 0.17]	0.85	110	0.03 [-0.16; 0.22]	0.73	110	0.03 [-0.16; 0.23]	0.72	109	0.01 [-0.18; 0.20]	0.90	109	0.05 [-0.14; 0.24]	0.63
	mod1	111	0.02 [-0.18; 0.21]	0.86	111	-0.02 [-0.21; 0.17]	0.84	110	0.05 [-0.13; 0.23]	0.59	110	0.05 [-0.13; 0.24]	0.58	109	0.02 [-0.17; 0.22]	0.81	109	0.07 [-0.13; 0.26]	0.51
Phospholipids in XS-VLDL	unadj.	111	-0.03 [-0.22; 0.16]	0.78	111	-0.02 [-0.21; 0.17]	0.85	110	-0.03 [-0.22; 0.16]	0.76	110	-0.02 [-0.21; 0.17]	0.87	109	-0.01 [-0.20; 0.18]	0.94	109	-0.00 [-0.19; 0.19]	0.99
	mod1	111	-0.03 [-0.22; 0.16]	0.76	111	-0.03 [-0.22; 0.16]	0.77	110	-0.03 [-0.21; 0.15]	0.75	110	-0.01 [-0.20; 0.17]	0.90	109	-0.00 [-0.20; 0.19]	0.98	109	0.01 [-0.18; 0.21]	0.91
Cholesterol in XS-VLDL	unadj.	111	-0.03 [-0.22; 0.16]	0.75	111	-0.01 [-0.20; 0.18]	0.95	110	-0.06 [-0.26; 0.13]	0.50	110	-0.06 [-0.25; 0.13]	0.56	109	-0.03 [-0.22; 0.16]	0.76	109	-0.05 [-0.24; 0.14]	0.63
	mod1	111	-0.04 [-0.23; 0.16]	0.71	111	-0.02 [-0.21; 0.17]	0.85	110	-0.07 [-0.25; 0.11]	0.45	110	-0.06 [-0.24; 0.13]	0.54	109	-0.03 [-0.22; 0.17]	0.78	109	-0.04 [-0.23; 0.16]	0.72
Triglycerides in XS-VLDL	unadj.	111	-0.02 [-0.21; 0.17]	0.85	111	-0.03 [-0.22; 0.16]	0.76	110	0.01 [-0.18; 0.20]	0.93	110	0.02 [-0.17; 0.21]	0.82	109	0.02 [-0.18; 0.21]	0.87	109	0.04 [-0.15; 0.23]	0.66
	mod1	111	-0.02 [-0.21; 0.17]	0.83	111	-0.04 [-0.23; 0.15]	0.69	110	0.01 [-0.17; 0.19]	0.91	110	0.03 [-0.16; 0.21]	0.79	109	0.02 [-0.17; 0.21]	0.83	109	0.06 [-0.14; 0.25]	0.57
Phospholipids in IDL	unadj.	111	-0.04 [-0.23; 0.15]	0.68	111	-0.01 [-0.20; 0.18]	0.95	110	-0.10 [-0.29; 0.09]	0.31	110	-0.10 [-0.29; 0.09]	0.32	109	-0.06 [-0.25; 0.13]	0.51	109	-0.09 [-0.28; 0.10]	0.34
	mod1	111	-0.04 [-0.24; 0.15]	0.65	111	-0.02 [-0.21; 0.17]	0.87	110	-0.10 [-0.28; 0.08]	0.28	110	-0.10 [-0.28; 0.09]	0.30	109	-0.06 [-0.25; 0.13]	0.54	109	-0.08 [-0.28; 0.11]	0.40
Cholesterol in IDL	unadj.	111	-0.04 [-0.23; 0.15]	0.70	111	0.00 [-0.19; 0.19]	0.97	110	-0.11 [-0.30; 0.08]	0.26	110	-0.11 [-0.30; 0.08]	0.25	109	-0.08 [-0.27; 0.12]	0.43	109	-0.12 [-0.31; 0.07]	0.23
	mod1	111	-0.04 [-0.23; 0.15]	0.69	111	-0.00 [-0.19; 0.19]	0.98	110	-0.11 [-0.29; 0.07]	0.24	110	-0.11 [-0.29; 0.08]	0.25	109	-0.07 [-0.27; 0.12]	0.47	109	-0.11 [-0.30; 0.09]	0.28
Triglycerides in IDL	unadj.	111	-0.03 [-0.21; 0.16]	0.79	111	-0.03 [-0.22; 0.16]	0.75	110	-0.01 [-0.20; 0.18]	0.93	110	0.00 [-0.19; 0.19]	0.99	109	0.02 [-0.18; 0.21]	0.87	109	0.03 [-0.16; 0.23]	0.73
	mod1	111	-0.03 [-0.23; 0.16]	0.73	111	-0.05 [-0.24; 0.14]	0.63	110	-0.02 [-0.20; 0.16]	0.84	110	-0.01 [-0.19; 0.18]	0.95	109	0.02 [-0.18; 0.21]	0.87	109	0.04 [-0.15; 0.24]	0.65
Phospholipids in L-LDL	unadj.	111	-0.01 [-0.20; 0.18]	0.93	111	0.03 [-0.16; 0.22]	0.73	110	-0.07 [-0.26; 0.12]	0.48	110	-0.07 [-0.26; 0.12]	0.45	109	-0.07 [-0.26; 0.12]	0.45	109	-0.12 [-0.31; 0.07]	0.23
	mod1	111	-0.01 [-0.20; 0.19]	0.95	111	0.03 [-0.16; 0.22]	0.76	110	-0.06 [-0.24; 0.12]	0.52	110	-0.06 [-0.25; 0.13]	0.51	109	-0.07 [-0.26; 0.13]	0.51	109	-0.10 [-0.30; 0.09]	0.30
Cholesterol in L-LDL	unadj.	111	-0.00 [-0.19; 0.19]	0.97	111	0.04 [-0.15; 0.23]	0.68	110	-0.07 [-0.26; 0.12]	0.50	110	-0.06 [-0.25; 0.13]	0.50	109	-0.07 [-0.27; 0.12]	0.44	109	-0.12 [-0.31; 0.07]	0.22
	mod1	111	-0.00 [-0.19; 0.19]	>0.99	111	0.04 [-0.15; 0.23]	0.69	110	-0.05 [-0.24; 0.13]	0.57	110	-0.05 [-0.24; 0.14]	0.59	109	-0.06 [-0.26; 0.13]	0.51	109	-0.10 [-0.30; 0.09]	0.30
Triglycerides in L-LDL	unadj.	111	-0.02 [-0.21; 0.17]	0.82	111	-0.02 [-0.21; 0.17]	0.80	110	-0.01 [-0.20; 0.18]	0.90	110	-0.00 [-0.19; 0.19]	0.97	109	0.01 [-0.18; 0.21]	0.89	109	0.03 [-0.17; 0.22]	0.79
	mod1	111	-0.03 [-0.22; 0.16]	0.75	111	-0.04 [-0.23; 0.15]	0.67	110	-0.02 [-0.21; 0.16]	0.79	110	-0.01 [-0.20; 0.17]	0.89	109	0.01 [-0.18; 0.21]	0.90	109	0.04 [-0.16; 0.23]	0.71
Phospholipids in M-LDL	unadj.	111	0.01 [-0.18; 0.20]	0.88	111	0.05 [-0.14; 0.24]	0.62	110	-0.04 [-0.23; 0.15]	0.71	110	-0.04 [-0.23; 0.15]	0.68	109	-0.06 [-0.25; 0.13]	0.54	109	-0.10 [-0.29; 0.09]	0.31
	mod1	111	0.02 [-0.18; 0.21]	0.84	111	0.05 [-0.15; 0.24]	0.63	110	-0.02 [-0.21; 0.16]	0.81	110	-0.02 [-0.21; 0.16]	0.80	109	-0.05 [-0.25; 0.15]	0.62	109	-0.08 [-0.28; 0.12]	0.41
Cholesterol in M-LDL	unadj.	111	0.02 [-0.17; 0.21]	0.82	111	0.06 [-0.13; 0.25]	0.56	110	-0.03 [-0.22; 0.16]	0.73	110	-0.04 [-0.23; 0.16]	0.71	109	-0.06 [-0.25; 0.13]	0.54	109	-0.10 [-0.29; 0.09]	0.30
	mod1	111	0.03 [-0.17; 0.22]	0.77	111	0.06 [-0.14; 0.25]	0.56	110	-0.02 [-0.20; 0.17]	0.85	110	-0.02 [-0.21; 0.17]	0.85	109	-0.05 [-0.25; 0.15]	0.63	109	-0.08 [-0.28; 0.11]	0.40
Triglycerides in M-LDL	unadj.	111	-0.01 [-0.20; 0.18]	0.92	111	-0.01 [-0.20; 0.18]	0.88	110	-0.00 [-0.19; 0.19]	0.98	110	0.01 [-0.18; 0.20]	0.93	109	0.01 [-0.18; 0.20]	0.93	109	0.02 [-0.17; 0.22]	0.81
	mod1	111	-0.02 [-0.21; 0.18]	0.87	111	-0.03 [-0.22; 0.16]	0.77	110	-0.01 [-0.19; 0.17]	0.92	110	0.01 [-0.18; 0.19]	0.96	109	0.01 [-0.18; 0.20]	0.91	109	0.04 [-0.16; 0.23]	0.72
Phospholipids in S-LDL	unadj.	111	-0.00 [-0.19; 0.19]	>0.99	111	0.03 [-0.16; 0.22]	0.75	110	-0.05 [-0.24; 0.14]	0.57	110	-0.05 [-0.24; 0.14]	0.59	109	-0.05 [-0.24; 0.14]	0.62	109	-0.08 [-0.27; 0.11]	0.39
	mod1	111	0.01 [-0.19; 0.20]	0.96	111	0.03 [-0.16; 0.22]	0.74	110	-0.04 [-0.22; 0.15]	0.70	110	-0.03 [-0.22; 0.16]	0.73	109	-0.03 [-0.23; 0.16]	0.73	109	-0.07 [-0.26; 0.13]	0.52
Cholesterol in S-LDL	unadj.	111	0.02 [-0.17; 0.21]	0.86	111	0.05 [-0.14; 0.24]	0.59	110	-0.05 [-0.24; 0.14]	0.64	110	-0.05 [-0.24; 0.15]	0.64	109	-0.06 [-0.25; 0.13]	0.52	109	-0.10 [-0.29; 0.09]	0.29
	mod1	111	0.02 [-0.17; 0.22]	0.81	111	0.05 [-0.14; 0.25]	0.59	110	-0.03 [-0.22; 0.16]	0.76	110	-0.03 [-0.22; 0.16]	0.78	109	-0.05 [-0.25; 0.15]	0.62	109	-0.09 [-0.28; 0.11]	0.39
Triglycerides in S-LDL	unadj.	111	0.01 [-0.18; 0.20]	0.88	111	-0.00 [-0.19; 0.19]	0.99	110	0.02 [-0.17; 0.21]	0.86	110	0.03 [-0.16; 0.22]	0.76	109	0.01 [-0.18; 0.20]	0.94	109	0.03 [-0.16; 0.22]	0.76
	mod1	111	0.01 [-0.18; 0.20]	0.92	111	-0.01 [-0.21; 0.18]	0.88	110	0.01 [-0.17; 0.20]	0.88	110	0.03 [-0.16; 0.22]	0.75	109	0.01 [-0.18; 0.21]	0.91	109	0.04 [-0.15; 0.24]	0.65
Phospholipids in XL-HDL	unadj.	111	-0.12 [-0.31; 0.07]	0.21	111	-0.16 [-0.35; 0.03]	0.094	110	-0.17 [-0.35; 0.02]	0.082	110	-0.17 [-0.36; 0.01]	0.070	109	-0.00 [-0.19; 0.19]	0.98	109	0.04 [-0.15; 0.24]	0.64

		HOMA2-IR (C-peptide)			HOMA2-IR (Insulin)			AUC (C-peptide/Glucose)			AUC (Insulin/Glucose)			oDI (C-peptide)			oDI (Insulin)		
Metabolite	Model	n	Beta ± 95% CI	p-value	n	Beta ± 95% CI	p-value	n	Beta ± 95% CI	p-value	n	Beta ± 95% CI	p-value	n	Beta ± 95% CI	p-value	n	Beta ± 95% CI	p-value
	mod1	111	-0.13 [-0.32; 0.05]	0.16	111	-0.18 [-0.36; 0.01]	0.064	110	-0.20 [-0.38; -0.02]	0.029	110	-0.20 [-0.38; -0.02]	0.027	109	-0.02 [-0.21; 0.17]	0.84	109	0.03 [-0.16; 0.23]	0.74
Cholesterol in XL-HDL	unadj.	111	-0.11 [-0.30; 0.08]	0.24	111	-0.14 [-0.33; 0.05]	0.15	110	-0.18 [-0.37; 0.01]	0.062	110	-0.19 [-0.37; 0.00]	0.050	109	-0.01 [-0.20; 0.18]	0.92	109	0.02 [-0.18; 0.21]	0.87
	mod1	111	-0.13 [-0.31; 0.06]	0.19	111	-0.15 [-0.34; 0.03]	0.11	110	-0.21 [-0.38; -0.03]	0.022	110	-0.21 [-0.39; -0.03]	0.020	109	-0.02 [-0.22; 0.17]	0.80	109	0.01 [-0.19; 0.20]	0.96
Triglycerides in XL-HDL	unadj.	111	-0.04 [-0.23; 0.15]	0.69	111	-0.07 [-0.26; 0.12]	0.48	110	-0.05 [-0.24; 0.14]	0.63	110	-0.04 [-0.23; 0.15]	0.71	109	-0.00 [-0.19; 0.19]	>0.99	109	0.04 [-0.15; 0.23]	0.66
	mod1	111	-0.04 [-0.24; 0.15]	0.65	111	-0.08 [-0.27; 0.11]	0.40	110	-0.05 [-0.24; 0.13]	0.56	110	-0.04 [-0.23; 0.14]	0.67	109	-0.00 [-0.19; 0.19]	>0.99	109	0.05 [-0.14; 0.25]	0.60
Phospholipids in L-HDL	unadj.	111	-0.13 [-0.32; 0.06]	0.18	111	-0.16 [-0.35; 0.02]	0.084	110	-0.14 [-0.33; 0.05]	0.16	110	-0.15 [-0.33; 0.04]	0.13	109	-0.02 [-0.21; 0.17]	0.85	109	0.04 [-0.16; 0.23]	0.71
	mod1	111	-0.15 [-0.34; 0.04]	0.12	111	-0.19 [-0.38; -0.00]	0.048	110	-0.18 [-0.36; -0.00]	0.045	110	-0.19 [-0.37; -0.01]	0.040	109	-0.04 [-0.24; 0.15]	0.65	109	0.02 [-0.18; 0.21]	0.87
Cholesterol in L-HDL	unadj.	111	-0.13 [-0.31; 0.06]	0.19	111	-0.15 [-0.33; 0.04]	0.12	110	-0.16 [-0.34; 0.03]	0.11	110	-0.16 [-0.35; 0.02]	0.088	109	-0.03 [-0.22; 0.16]	0.75	109	0.00 [-0.19; 0.20]	0.97
	mod1	111	-0.14 [-0.33; 0.05]	0.14	111	-0.16 [-0.35; 0.02]	0.084	110	-0.19 [-0.37; -0.01]	0.034	110	-0.20 [-0.38; -0.02]	0.031	109	-0.05 [-0.25; 0.14]	0.59	109	-0.01 [-0.21; 0.18]	0.89
Triglycerides in L-HDL	unadj.	111	-0.09 [-0.28; 0.10]	0.36	111	-0.14 [-0.32; 0.05]	0.16	110	-0.08 [-0.27; 0.11]	0.43	110	-0.07 [-0.26; 0.12]	0.46	109	-0.01 [-0.20; 0.18]	0.93	109	0.07 [-0.12; 0.26]	0.49
	mod1	111	-0.10 [-0.29; 0.09]	0.30	111	-0.15 [-0.34; 0.03]	0.10	110	-0.10 [-0.28; 0.08]	0.27	110	-0.09 [-0.27; 0.09]	0.33	109	-0.02 [-0.21; 0.17]	0.85	109	0.06 [-0.13; 0.26]	0.51
Phospholipids in M-HDL	unadj.	111	-0.08 [-0.27; 0.11]	0.40	111	-0.12 [-0.31; 0.07]	0.22	110	-0.01 [-0.20; 0.18]	0.92	110	-0.02 [-0.21; 0.17]	0.84	109	0.00 [-0.19; 0.19]	0.98	109	0.06 [-0.13; 0.25]	0.53
	mod1	111	-0.11 [-0.30; 0.08]	0.26	111	-0.15 [-0.34; 0.04]	0.11	110	-0.07 [-0.26; 0.11]	0.44	110	-0.08 [-0.26; 0.11]	0.42	109	-0.03 [-0.23; 0.17]	0.76	109	0.04 [-0.16; 0.24]	0.69
Cholesterol in M-HDL	unadj.	111	-0.08 [-0.27; 0.11]	0.38	111	-0.10 [-0.29; 0.09]	0.29	110	-0.04 [-0.23; 0.15]	0.69	110	-0.05 [-0.24; 0.14]	0.60	109	-0.02 [-0.21; 0.17]	0.81	109	0.01 [-0.18; 0.20]	0.90
	mod1	111	-0.10 [-0.30; 0.09]	0.28	111	-0.12 [-0.31; 0.06]	0.20	110	-0.08 [-0.27; 0.10]	0.37	110	-0.09 [-0.28; 0.09]	0.32	109	-0.05 [-0.24; 0.15]	0.62	109	-0.01 [-0.20; 0.19]	0.93
Triglycerides in M-HDL	unadj.	111	-0.03 [-0.22; 0.16]	0.78	111	-0.07 [-0.26; 0.12]	0.48	110	0.02 [-0.17; 0.21]	0.81	110	0.03 [-0.16; 0.22]	0.72	109	0.02 [-0.18; 0.21]	0.87	109	0.08 [-0.11; 0.27]	0.40
	mod1	111	-0.04 [-0.23; 0.15]	0.70	111	-0.09 [-0.28; 0.10]	0.36	110	0.00 [-0.18; 0.19]	0.96	110	0.02 [-0.17; 0.20]	0.83	109	0.01 [-0.18; 0.20]	0.92	109	0.09 [-0.11; 0.28]	0.38
Phospholipids in S-HDL	unadj.	111	-0.03 [-0.22; 0.16]	0.77	111	-0.06 [-0.25; 0.13]	0.50	110	0.07 [-0.12; 0.26]	0.44	110	0.05 [-0.14; 0.24]	0.58	109	0.01 [-0.18; 0.20]	0.92	109	0.06 [-0.13; 0.25]	0.53
	mod1	111	-0.05 [-0.24; 0.14]	0.59	111	-0.10 [-0.28; 0.09]	0.32	110	0.03 [-0.16; 0.21]	0.78	110	0.01 [-0.17; 0.20]	0.90	109	-0.01 [-0.21; 0.18]	0.89	109	0.05 [-0.14; 0.24]	0.61
Cholesterol in S-HDL	unadj.	111	0.01 [-0.18; 0.20]	0.92	111	0.01 [-0.18; 0.20]	0.88	110	0.09 [-0.10; 0.28]	0.35	110	0.06 [-0.13; 0.25]	0.53	109	-0.01 [-0.21; 0.18]	0.88	109	-0.02 [-0.21; 0.18]	0.87
	mod1	111	0.00 [-0.19; 0.19]	>0.99	111	0.00 [-0.19; 0.19]	>0.99	110	0.07 [-0.11; 0.25]	0.42	110	0.05 [-0.14; 0.23]	0.61	109	-0.02 [-0.21; 0.17]	0.84	109	-0.02 [-0.21; 0.18]	0.88
Triglycerides in S-HDL	unadj.	111	0.01 [-0.18; 0.20]	0.90	111	-0.02 [-0.21; 0.17]	0.86	110	0.05 [-0.14; 0.24]	0.61	110	0.06 [-0.13; 0.25]	0.54	109	0.02 [-0.17; 0.21]	0.81	109	0.06 [-0.13; 0.25]	0.52
	mod1	111	0.01 [-0.18; 0.20]	0.93	111	-0.03 [-0.22; 0.16]	0.77	110	0.05 [-0.13; 0.23]	0.60	110	0.06 [-0.12; 0.25]	0.52	109	0.03 [-0.17; 0.22]	0.79	109	0.08 [-0.12; 0.27]	0.44
**Apolipoproteins**																			
Apolipoprotein B	unadj.	111	0.00 [-0.19; 0.19]	0.98	111	0.03 [-0.16; 0.22]	0.75	110	-0.05 [-0.24; 0.14]	0.61	110	-0.05 [-0.24; 0.14]	0.63	109	-0.05 [-0.24; 0.14]	0.59	109	-0.08 [-0.27; 0.11]	0.40
	mod1	111	0.00 [-0.19; 0.20]	0.96	111	0.03 [-0.16; 0.22]	0.78	110	-0.04 [-0.22; 0.15]	0.68	110	-0.03 [-0.22; 0.15]	0.72	109	-0.04 [-0.24; 0.15]	0.67	109	-0.07 [-0.26; 0.13]	0.51
Apolipoprotein A1	unadj.	111	-0.11 [-0.30; 0.08]	0.24	111	-0.16 [-0.34; 0.03]	0.10	110	-0.07 [-0.26; 0.12]	0.44	110	-0.09 [-0.28; 0.10]	0.34	109	-0.01 [-0.20; 0.18]	0.92	109	0.05 [-0.14; 0.25]	0.57
	mod1	111	-0.14 [-0.33; 0.05]	0.15	111	-0.19 [-0.37; -0.00]	0.048	110	-0.13 [-0.31; 0.05]	0.16	110	-0.14 [-0.33; 0.04]	0.13	109	-0.04 [-0.23; 0.16]	0.69	109	0.04 [-0.16; 0.23]	0.72
Ratio of apolipoprotein B to	unadj.	111	0.08 [-0.11; 0.27]	0.41	111	0.12 [-0.06; 0.31]	0.19	110	-0.02 [-0.21; 0.17]	0.84	110	-0.00 [-0.19; 0.19]	>0.99	109	-0.09 [-0.28; 0.10]	0.35	109	-0.15 [-0.34; 0.04]	0.11
apolipoprotein A1	mod1	111	0.10 [-0.09; 0.29]	0.31	111	0.15 [-0.04; 0.33]	0.13	110	0.02 [-0.16; 0.21]	0.82	110	0.04 [-0.15; 0.23]	0.68	109	-0.07 [-0.27; 0.12]	0.47	109	-0.14 [-0.33; 0.06]	0.17
**Fatty acids**																			
Total fatty acids	unadj.	109	-0.03 [-0.22; 0.16]	0.77	109	-0.03 [-0.22; 0.16]	0.75	108	-0.02 [-0.21; 0.17]	0.84	108	-0.01 [-0.20; 0.18]	0.91	107	0.02 [-0.17; 0.21]	0.84	107	0.03 [-0.17; 0.22]	0.78
	mod1	109	-0.03 [-0.23; 0.16]	0.74	109	-0.04 [-0.24; 0.15]	0.66	108	-0.02 [-0.21; 0.17]	0.84	108	-0.01 [-0.20; 0.19]	0.96	107	0.03 [-0.17; 0.23]	0.79	107	0.04 [-0.16; 0.24]	0.66
Degree of unsaturation	unadj.	109	-0.15 [-0.34; 0.03]	0.11	109	-0.10 [-0.29; 0.09]	0.29	108	-0.15 [-0.34; 0.04]	0.12	108	-0.18 [-0.37; 0.01]	0.064	107	-0.00 [-0.20; 0.19]	0.98	107	-0.06 [-0.25; 0.14]	0.57
	mod1	109	-0.15 [-0.34; 0.04]	0.13	109	-0.09 [-0.28; 0.10]	0.34	108	-0.13 [-0.31; 0.05]	0.17	108	-0.16 [-0.34; 0.03]	0.096	107	0.01 [-0.18; 0.21]	0.92	107	-0.04 [-0.24; 0.15]	0.66
Omega-3 fatty acids	unadj.	109	-0.04 [-0.23; 0.15]	0.68	109	-0.01 [-0.20; 0.18]	0.93	108	0.04 [-0.15; 0.24]	0.66	108	0.03 [-0.17; 0.22]	0.79	107	0.11 [-0.09; 0.30]	0.27	107	0.07 [-0.12; 0.27]	0.45
	mod1	109	-0.04 [-0.24; 0.15]	0.67	109	-0.01 [-0.20; 0.18]	0.90	108	0.05 [-0.14; 0.23]	0.61	108	0.03 [-0.15; 0.22]	0.73	107	0.11 [-0.08; 0.31]	0.25	107	0.09 [-0.11; 0.28]	0.39
Omega-6 fatty acids	unadj.	109	-0.09 [-0.28; 0.10]	0.34	109	-0.08 [-0.28; 0.11]	0.38	108	-0.12 [-0.32; 0.07]	0.20	108	-0.12 [-0.31; 0.07]	0.22	107	-0.05 [-0.25; 0.14]	0.58	107	-0.05 [-0.24; 0.15]	0.63
	mod1	109	-0.09 [-0.29; 0.10]	0.35	109	-0.09 [-0.28; 0.11]	0.36	108	-0.11 [-0.30; 0.07]	0.24	108	-0.10 [-0.29; 0.09]	0.29	107	-0.04 [-0.24; 0.16]	0.66	107	-0.03 [-0.23; 0.17]	0.76
PUFA	unadj.	109	-0.09 [-0.28; 0.10]	0.36	109	-0.07 [-0.27; 0.12]	0.44	108	-0.09 [-0.29; 0.10]	0.33	108	-0.09 [-0.29; 0.10]	0.33	107	-0.02 [-0.21; 0.18]	0.85	107	-0.02 [-0.22; 0.17]	0.82
	mod1	109	-0.09 [-0.29; 0.11]	0.36	109	-0.08 [-0.27; 0.12]	0.42	108	-0.08 [-0.27; 0.12]	0.38	108	-0.08 [-0.27; 0.11]	0.41	107	-0.01 [-0.21; 0.19]	0.94	107	-0.00 [-0.20; 0.20]	0.97
MUFA	unadj.	109	0.00 [-0.19; 0.20]	0.96	109	-0.00 [-0.20; 0.19]	0.96	108	0.00 [-0.19; 0.19]	0.99	108	0.02 [-0.17; 0.21]	0.85	107	0.02 [-0.18; 0.21]	0.87	107	0.03 [-0.17; 0.22]	0.79
	mod1	109	0.00 [-0.20; 0.20]	>0.99	109	-0.02 [-0.21; 0.18]	0.88	108	0.00 [-0.19; 0.19]	>0.99	108	0.02 [-0.17; 0.21]	0.81	107	0.02 [-0.18; 0.22]	0.84	107	0.04 [-0.16; 0.24]	0.68
SFA	unadj.	109	-0.01 [-0.20; 0.18]	0.91	109	-0.02 [-0.21; 0.17]	0.83	108	0.02 [-0.18; 0.21]	0.86	108	0.02 [-0.17; 0.22]	0.82	107	0.05 [-0.14; 0.24]	0.61	107	0.06 [-0.13; 0.25]	0.54
	mod1	109	-0.02 [-0.21; 0.18]	0.86	109	-0.04 [-0.23; 0.16]	0.71	108	0.01 [-0.18; 0.20]	0.91	108	0.02 [-0.17; 0.21]	0.83	107	0.05 [-0.15; 0.25]	0.60	107	0.08 [-0.12; 0.27]	0.45
Linoleic acid	unadj.	109	-0.11 [-0.30; 0.08]	0.24	109	-0.11 [-0.30; 0.08]	0.26	108	-0.13 [-0.32; 0.06]	0.18	108	-0.13 [-0.32; 0.06]	0.19	107	-0.05 [-0.24; 0.14]	0.61	107	-0.04 [-0.23; 0.16]	0.70
	mod1	109	-0.11 [-0.31; 0.08]	0.26	109	-0.11 [-0.31; 0.08]	0.25	108	-0.12 [-0.31; 0.07]	0.23	108	-0.11 [-0.30; 0.08]	0.27	107	-0.04 [-0.24; 0.16]	0.71	107	-0.02 [-0.22; 0.18]	0.86
Docosahexaenoic acid	unadj.	109	-0.05 [-0.24; 0.14]	0.63	109	-0.01 [-0.20; 0.18]	0.93	108	0.06 [-0.13; 0.25]	0.53	108	0.04 [-0.15; 0.23]	0.67	107	0.07 [-0.12; 0.27]	0.45	107	0.04 [-0.16; 0.23]	0.70
	mod1	109	-0.05 [-0.25; 0.14]	0.59	109	-0.02 [-0.21; 0.17]	0.86	108	0.06 [-0.13; 0.24]	0.53	108	0.04 [-0.15; 0.23]	0.66	107	0.08 [-0.12; 0.27]	0.44	107	0.05 [-0.15; 0.24]	0.64
Omega-3 FA / total FA	unadj.	109	-0.08 [-0.27; 0.11]	0.43	109	-0.03 [-0.22; 0.16]	0.78	108	0.04 [-0.16; 0.23]	0.71	108	0.01 [-0.18; 0.20]	0.93	107	0.14 [-0.05; 0.33]	0.15	107	0.09 [-0.10; 0.28]	0.35
	mod1	109	-0.07 [-0.27; 0.12]	0.44	109	-0.02 [-0.21; 0.16]	0.80	108	0.04 [-0.14; 0.22]	0.66	108	0.01 [-0.17; 0.20]	0.88	107	0.14 [-0.05; 0.33]	0.14	107	0.09 [-0.10; 0.29]	0.35
Omega-6 FA / total FA	unadj.	109	-0.05 [-0.24; 0.14]	0.59	109	-0.03 [-0.22; 0.16]	0.78	108	-0.13 [-0.32; 0.06]	0.17	108	-0.13 [-0.32; 0.06]	0.17	107	-0.13 [-0.32; 0.06]	0.19	107	-0.14 [-0.34; 0.05]	0.14
	mod1	109	-0.03 [-0.23; 0.16]	0.72	109	-0.00 [-0.19; 0.19]	0.99	108	-0.10 [-0.28; 0.08]	0.29	108	-0.11 [-0.29; 0.08]	0.26	107	-0.12 [-0.31; 0.08]	0.23	107	-0.15 [-0.34; 0.05]	0.14
PUFA / total FA	unadj.	109	-0.08 [-0.27; 0.11]	0.43	109	-0.04 [-0.23; 0.16]	0.72	108	-0.12 [-0.31; 0.07]	0.23	108	-0.13 [-0.32; 0.06]	0.19	107	-0.08 [-0.27; 0.12]	0.43	107	-0.11 [-0.30; 0.08]	0.26
	mod1	109	-0.06 [-0.25; 0.13]	0.54	109	-0.01 [-0.20; 0.18]	0.92	108	-0.08 [-0.27; 0.10]	0.38	108	-0.10 [-0.28; 0.09]	0.30	107	-0.06 [-0.26; 0.13]	0.51	107	-0.11 [-0.31; 0.08]	0.26
MUFA / total FA	unadj.	109	0.05 [-0.14; 0.24]	0.62	109	0.02 [-0.17; 0.21]	0.84	108	0.05 [-0.15; 0.24]	0.64	108	0.06 [-0.13; 0.26]	0.52	107	0.02 [-0.17; 0.22]	0.80	107	0.05 [-0.14; 0.24]	0.61
	mod1	109	0.04 [-0.15; 0.23]	0.68	109	0.00 [-0.19; 0.19]	0.97	108	0.03 [-0.15; 0.21]	0.75	108	0.05 [-0.13; 0.24]	0.58	107	0.02 [-0.17; 0.22]	0.84	107	0.06 [-0.14; 0.25]	0.57
SFA / total FA	unadj.	109	0.08 [-0.11; 0.27]	0.41	109	0.04 [-0.15; 0.23]	0.66	108	0.17 [-0.02; 0.36]	0.086	108	0.16 [-0.03; 0.35]	0.10	107	0.12 [-0.07; 0.31]	0.22	107	0.15 [-0.04; 0.34]	0.13
	mod1	109	0.06 [-0.13; 0.25]	0.54	109	0.01 [-0.18; 0.21]	0.88	108	0.12 [-0.06; 0.30]	0.19	108	0.12 [-0.07; 0.30]	0.21	107	0.10 [-0.09; 0.29]	0.31	107	0.14 [-0.06; 0.33]	0.16

		HOMA2-IR (C-peptide)			HOMA2-IR (Insulin)			AUC (C-peptide/Glucose)			AUC (Insulin/Glucose)			oDI (C-peptide)			oDI (Insulin)		
**Metabolite**	**Model**	**n**	**Beta ± 95% CI**	**p-value**	**n**	**Beta ± 95% CI**	**p-value**	**n**	**Beta ± 95% CI**	**p-value**	**n**	**Beta ± 95% CI**	**p-value**	**n**	**Beta ± 95% CI**	**p-value**	**n**	**Beta ± 95% CI**	**p-value**
Linoleic acid / total FA	unadj.	109	-0.12 [-0.31; 0.07]	0.21	109	-0.10 [-0.29; 0.09]	0.30	108	-0.17 [-0.36; 0.02]	0.076	108	-0.18 [-0.37; 0.01]	0.065	107	-0.12 [-0.31; 0.07]	0.21	107	-0.13 [-0.32; 0.06]	0.18
	mod1	109	-0.10 [-0.29; 0.09]	0.30	109	-0.07 [-0.26; 0.12]	0.44	108	-0.13 [-0.31; 0.06]	0.17	108	-0.14 [-0.32; 0.05]	0.14	107	-0.10 [-0.30; 0.09]	0.29	107	-0.12 [-0.32; 0.07]	0.22
Docosahexaenoic acid / total FA	unadj.	109	-0.04 [-0.24; 0.15]	0.65	109	0.01 [-0.18; 0.20]	0.91	108	0.06 [-0.13; 0.26]	0.51	108	0.05 [-0.15; 0.24]	0.64	107	0.07 [-0.12; 0.27]	0.45	107	0.02 [-0.17; 0.22]	0.83
	mod1	109	-0.04 [-0.24; 0.15]	0.64	109	0.01 [-0.18; 0.20]	0.90	108	0.06 [-0.12; 0.25]	0.49	108	0.04 [-0.14; 0.23]	0.64	107	0.07 [-0.12; 0.26]	0.47	107	0.02 [-0.18; 0.21]	0.86
PUFA / MUFA	unadj.	109	-0.07 [-0.26; 0.13]	0.50	109	-0.02 [-0.22; 0.17]	0.80	108	-0.09 [-0.29; 0.10]	0.33	108	-0.11 [-0.30; 0.09]	0.28	107	-0.06 [-0.26; 0.13]	0.52	107	-0.10 [-0.29; 0.09]	0.32
	mod1	109	-0.05 [-0.24; 0.14]	0.62	109	0.00 [-0.19; 0.19]	0.98	108	-0.06 [-0.25; 0.12]	0.51	108	-0.08 [-0.26; 0.11]	0.41	107	-0.05 [-0.25; 0.14]	0.60	107	-0.10 [-0.30; 0.09]	0.31
Omega-6 FA / omega-3 FA	unadj.	111	0.11 [-0.07; 0.30]	0.23	111	0.06 [-0.13; 0.25]	0.52	110	0.00 [-0.19; 0.19]	0.97	110	0.01 [-0.18; 0.20]	0.89	109	-0.18 [-0.37; 0.01]	0.058	109	-0.14 [-0.33; 0.05]	0.15
	mod1	111	0.12 [-0.07; 0.31]	0.21	111	0.07 [-0.12; 0.26]	0.46	110	0.01 [-0.17; 0.19]	0.88	110	0.02 [-0.16; 0.21]	0.81	109	-0.18 [-0.37; 0.01]	0.063	109	-0.14 [-0.33; 0.05]	0.15
**Phospholipids**																			
Phosphoglycerides	unadj.	108	-0.08 [-0.27; 0.12]	0.43	108	-0.07 [-0.26; 0.12]	0.49	107	-0.03 [-0.22; 0.17]	0.79	107	-0.03 [-0.23; 0.16]	0.73	106	0.03 [-0.16; 0.23]	0.74	106	0.02 [-0.17; 0.22]	0.81
	mod1	108	-0.09 [-0.28; 0.11]	0.37	108	-0.09 [-0.28; 0.10]	0.36	107	-0.04 [-0.23; 0.14]	0.65	107	-0.04 [-0.23; 0.14]	0.64	106	0.03 [-0.17; 0.23]	0.78	106	0.03 [-0.17; 0.23]	0.74
Ratio of triglycerides to phosphoglycerides	unadj.	108	0.04 [-0.15; 0.23]	0.68	108	0.00 [-0.19; 0.20]	0.97	107	0.09 [-0.10; 0.29]	0.33	107	0.09 [-0.11; 0.28]	0.38	106	0.09 [-0.10; 0.29]	0.35	106	0.11 [-0.08; 0.30]	0.26
	mod1	108	0.04 [-0.15; 0.24]	0.67	108	-0.00 [-0.19; 0.19]	>0.99	107	0.10 [-0.08; 0.28]	0.28	107	0.09 [-0.09; 0.28]	0.32	106	0.10 [-0.10; 0.29]	0.32	106	0.12 [-0.07; 0.32]	0.22
Total cholines	unadj.	108	-0.08 [-0.27; 0.11]	0.40	108	-0.07 [-0.26; 0.12]	0.47	107	-0.04 [-0.24; 0.15]	0.65	107	-0.06 [-0.25; 0.14]	0.57	106	0.02 [-0.17; 0.22]	0.83	106	0.01 [-0.19; 0.20]	0.93
	mod1	108	-0.09 [-0.29; 0.10]	0.35	108	-0.09 [-0.28; 0.10]	0.35	107	-0.06 [-0.25; 0.13]	0.53	107	-0.06 [-0.25; 0.12]	0.50	106	0.02 [-0.18; 0.22]	0.86	106	0.02 [-0.18; 0.22]	0.85
Phosphatidylcholines	unadj.	108	-0.09 [-0.28; 0.10]	0.36	108	-0.08 [-0.27; 0.11]	0.40	107	-0.04 [-0.23; 0.15]	0.67	107	-0.05 [-0.24; 0.14]	0.60	106	0.02 [-0.17; 0.21]	0.84	106	0.01 [-0.18; 0.21]	0.89
	mod1	108	-0.10 [-0.30; 0.09]	0.30	108	-0.10 [-0.30; 0.09]	0.29	107	-0.06 [-0.25; 0.13]	0.53	107	-0.06 [-0.25; 0.13]	0.51	106	0.01 [-0.18; 0.21]	0.89	106	0.02 [-0.18; 0.22]	0.83
Sphingomyelins	unadj.	108	-0.06 [-0.25; 0.14]	0.56	108	-0.00 [-0.20; 0.19]	0.96	107	-0.14 [-0.33; 0.06]	0.16	107	-0.15 [-0.34; 0.04]	0.12	106	-0.05 [-0.25; 0.14]	0.59	106	-0.12 [-0.31; 0.08]	0.23
	mod1	108	-0.07 [-0.26; 0.13]	0.51	108	-0.02 [-0.21; 0.17]	0.85	107	-0.15 [-0.33; 0.03]	0.11	107	-0.16 [-0.35; 0.03]	0.091	106	-0.06 [-0.25; 0.14]	0.57	106	-0.11 [-0.31; 0.09]	0.26

Supplementary table 5 – Associations between cord serum metabolites and insulin secretion and insulin resistance in the offspring at nine years age

Associations are given for regression coefficients (beta) with 95% confidence intervals (CI). Adjusted model 1 is adjusted for maternal pre-pregnancy BMI. P-values below 0.01 and 0.0038 are denoted with * and **, respectively. Cord blood lipoprotein lipids were log converted and marked with "*log*" in the table.

			HOMA2-IR (C-peptide)			HOMA2-IR (Insulin)			AUC (C-peptide/Glucose)			AUC (Insulin/Glucose)			oDI (C-peptide)			oDI (Insulin)		
**Metabolite**		**Model**	**n**	**Beta ± 95% CI**	**p-value**	**n**	**Beta ± 95% CI**	**p-value**	**n**	**Beta ± 95% CI**	**p-value**	**n**	**Beta ± 95% CI**	**p-value**	**n**	**Beta ± 95% CI**	**p-value**	**n**	**Beta ± 95% CI**	**p-value**
**Lipids**																				
Total cholesterol		unadj.	114	-0.08 [-0.26; 0.11]	0.42	114	-0.05 [-0.24; 0.14]	0.60	113	-0.16 [-0.35; 0.02]	0.085	113	-0.12 [-0.31; 0.07]	0.21	111	-0.09 [-0.28; 0.10]	0.35	111	-0.07 [-0.26; 0.12]	0.45
		mod1	114	-0.08 [-0.26; 0.11]	0.42	114	-0.05 [-0.24; 0.13]	0.58	113	-0.16 [-0.34; 0.01]	0.072	113	-0.12 [-0.30; 0.06]	0.20	111	-0.09 [-0.28; 0.10]	0.36	111	-0.07 [-0.26; 0.12]	0.49
Total cholesterol minus HDL	*log*	unadj.	114	-0.03 [-0.22; 0.15]	0.73	114	0.00 [-0.18; 0.19]	0.98	113	-0.12 [-0.30; 0.07]	0.21	113	-0.07 [-0.26; 0.11]	0.44	111	-0.11 [-0.30; 0.08]	0.26	111	-0.10 [-0.29; 0.09]	0.29
cholesterol	*log*	mod1	114	-0.03 [-0.22; 0.16]	0.75	114	0.01 [-0.18; 0.19]	0.95	113	-0.11 [-0.29; 0.07]	0.22	113	-0.07 [-0.25; 0.11]	0.46	111	-0.11 [-0.29; 0.08]	0.27	111	-0.10 [-0.29; 0.09]	0.29
Remnant cholesterol	*log*	unadj.	114	-0.04 [-0.22; 0.15]	0.69	114	0.01 [-0.18; 0.19]	0.94	113	-0.12 [-0.30; 0.07]	0.22	113	-0.07 [-0.26; 0.12]	0.48	111	-0.13 [-0.32; 0.06]	0.18	111	-0.13 [-0.32; 0.06]	0.18
	log	mod1	114	-0.05 [-0.24; 0.14]	0.61	114	-0.01 [-0.19; 0.18]	0.94	113	-0.14 [-0.32; 0.03]	0.11	113	-0.09 [-0.27; 0.09]	0.33	111	-0.14 [-0.33; 0.05]	0.14	111	-0.13 [-0.32; 0.06]	0.17
VLDL cholesterol	*log*	unadj.	114	-0.02 [-0.21; 0.17]	0.83	114	0.03 [-0.16; 0.21]	0.77	113	-0.09 [-0.28; 0.09]	0.32	113	-0.04 [-0.23; 0.15]	0.65	111	-0.15 [-0.33; 0.04]	0.13	111	-0.15 [-0.34; 0.04]	0.11
	log	mod1	114	-0.04 [-0.22; 0.15]	0.71	114	0.01 [-0.18; 0.20]	0.91	113	-0.13 [-0.31; 0.05]	0.15	113	-0.07 [-0.25; 0.11]	0.43	111	-0.16 [-0.35; 0.02]	0.087	111	-0.16 [-0.35; 0.03]	0.096
Clinical LDL cholesterol	*log*	unadj.	114	-0.05 [-0.24; 0.14]	0.60	114	-0.03 [-0.22; 0.16]	0.75	113	-0.12 [-0.30; 0.07]	0.22	113	-0.08 [-0.27; 0.10]	0.38	111	-0.05 [-0.24; 0.14]	0.63	111	-0.03 [-0.22; 0.16]	0.74
	log	mod1	114	-0.04 [-0.22; 0.15]	0.70	114	-0.01 [-0.20; 0.17]	0.89	113	-0.09 [-0.27; 0.09]	0.33	113	-0.06 [-0.24; 0.12]	0.53	111	-0.03 [-0.22; 0.16]	0.72	111	-0.03 [-0.22; 0.16]	0.75
LDL cholesterol	*log*	unadj.	114	-0.06 [-0.24; 0.13]	0.54	114	-0.04 [-0.23; 0.15]	0.67	113	-0.11 [-0.30; 0.07]	0.23	113	-0.08 [-0.27; 0.11]	0.39	111	-0.03 [-0.22; 0.16]	0.73	111	-0.01 [-0.20; 0.18]	0.89
	log	mod1	114	-0.04 [-0.23; 0.14]	0.64	114	-0.02 [-0.21; 0.17]	0.82	113	-0.08 [-0.26; 0.10]	0.37	113	-0.05 [-0.24; 0.13]	0.57	111	-0.02 [-0.21; 0.17]	0.83	111	-0.01 [-0.20; 0.18]	0.91
HDL cholesterol	*log*	unadj.	114	-0.05 [-0.24; 0.14]	0.60	114	-0.04 [-0.22; 0.15]	0.69	113	-0.16 [-0.34; 0.03]	0.098	113	-0.12 [-0.31; 0.07]	0.21	111	-0.07 [-0.26; 0.12]	0.45	111	-0.05 [-0.24; 0.14]	0.59
	log	mod1	114	-0.03 [-0.22; 0.16]	0.76	114	-0.01 [-0.20; 0.18]	0.90	113	-0.11 [-0.29; 0.07]	0.24	113	-0.08 [-0.26; 0.11]	0.40	111	-0.05 [-0.24; 0.14]	0.60	111	-0.04 [-0.24; 0.15]	0.66
Total triglycerides		unadj.	114	-0.09 [-0.27; 0.10]	0.37	114	-0.05 [-0.23; 0.14]	0.62	113	-0.12 [-0.31; 0.07]	0.21	113	-0.07 [-0.26; 0.11]	0.43	111	-0.08 [-0.27; 0.11]	0.42	111	-0.06 [-0.25; 0.13]	0.53
		mod1	114	-0.10 [-0.29; 0.09]	0.29	114	-0.07 [-0.25; 0.12]	0.48	113	-0.15 [-0.33; 0.02]	0.086	113	-0.10 [-0.28; 0.08]	0.26	111	-0.09 [-0.28; 0.10]	0.34	111	-0.06 [-0.26; 0.13]	0.51
Triglycerides in VLDL	*log*	unadj.	114	-0.01 [-0.20; 0.18]	0.91	114	0.04 [-0.15; 0.23]	0.66	113	-0.07 [-0.26; 0.12]	0.46	113	-0.01 [-0.19; 0.18]	0.95	111	-0.15 [-0.33; 0.04]	0.13	111	-0.15 [-0.34; 0.04]	0.12
	log	mod1	114	-0.03 [-0.22; 0.16]	0.76	114	0.02 [-0.16; 0.21]	0.81	113	-0.11 [-0.29; 0.06]	0.21	113	-0.04 [-0.23; 0.14]	0.64	111	-0.17 [-0.36; 0.02]	0.078	111	-0.17 [-0.36; 0.02]	0.087
Triglycerides in LDL	*log*	unadj.	114	-0.08 [-0.26; 0.11]	0.42	114	-0.03 [-0.22; 0.15]	0.72	113	-0.14 [-0.33; 0.05]	0.14	113	-0.09 [-0.28; 0.10]	0.35	111	-0.11 [-0.29; 0.08]	0.27	111	-0.10 [-0.29; 0.09]	0.32
	log	mod1	114	-0.08 [-0.26; 0.11]	0.41	114	-0.04 [-0.22; 0.15]	0.69	113	-0.15 [-0.32; 0.03]	0.10	113	-0.09 [-0.28; 0.09]	0.30	111	-0.11 [-0.30; 0.08]	0.25	111	-0.10 [-0.29; 0.09]	0.31
Triglycerides in HDL	*log*	unadj.	114	-0.04 [-0.23; 0.15]	0.68	114	0.01 [-0.18; 0.19]	0.94	113	-0.09 [-0.28; 0.10]	0.35	113	-0.02 [-0.21; 0.16]	0.80	111	-0.12 [-0.31; 0.06]	0.19	111	-0.11 [-0.29; 0.08]	0.27
	log	mod1	114	-0.05 [-0.24; 0.14]	0.61	114	-0.00 [-0.19; 0.18]	0.97	113	-0.11 [-0.29; 0.06]	0.21	113	-0.05 [-0.23; 0.14]	0.62	111	-0.14 [-0.32; 0.05]	0.15	111	-0.11 [-0.30; 0.08]	0.24
Total phospholipids in lipoprotein		unadj.	114	-0.08 [-0.26; 0.11]	0.42	114	-0.05 [-0.24; 0.14]	0.60	113	-0.15 [-0.34; 0.03]	0.10	113	-0.11 [-0.30; 0.08]	0.25	111	-0.09 [-0.28; 0.10]	0.34	111	-0.07 [-0.26; 0.12]	0.48
particles		mod1	114	-0.08 [-0.26; 0.11]	0.42	114	-0.05 [-0.24; 0.13]	0.58	113	-0.15 [-0.33; 0.02]	0.085	113	-0.11 [-0.29; 0.07]	0.23	111	-0.09 [-0.28; 0.10]	0.35	111	-0.06 [-0.25; 0.13]	0.51
Phospholipids in VLDL	*log*	unadj.	114	-0.03 [-0.21; 0.16]	0.78	114	0.02 [-0.17; 0.21]	0.82	113	-0.09 [-0.28; 0.10]	0.34	113	-0.04 [-0.23; 0.15]	0.69	111	-0.14 [-0.33; 0.05]	0.13	111	-0.15 [-0.34; 0.04]	0.12
	log	mod1	114	-0.04 [-0.23; 0.15]	0.67	114	0.01 [-0.18; 0.19]	0.94	113	-0.12 [-0.30; 0.05]	0.17	113	-0.07 [-0.25; 0.12]	0.47	111	-0.16 [-0.35; 0.03]	0.094	111	-0.16 [-0.35; 0.03]	0.10
Phospholipids in LDL	*log*	unadj.	114	-0.04 [-0.23; 0.15]	0.68	114	-0.01 [-0.20; 0.17]	0.90	113	-0.14 [-0.32; 0.05]	0.15	113	-0.09 [-0.28; 0.09]	0.32	111	-0.11 [-0.30; 0.08]	0.25	111	-0.10 [-0.29; 0.09]	0.28
	log	mod1	114	-0.03 [-0.22; 0.15]	0.71	114	-0.01 [-0.19; 0.18]	0.94	113	-0.13 [-0.30; 0.05]	0.16	113	-0.09 [-0.27; 0.10]	0.35	111	-0.11 [-0.29; 0.08]	0.26	111	-0.10 [-0.29; 0.09]	0.29
Phospholipids in HDL	*log*	unadj.	114	-0.04 [-0.22; 0.15]	0.71	114	-0.02 [-0.20; 0.17]	0.86	113	-0.13 [-0.32; 0.05]	0.16	113	-0.08 [-0.27; 0.10]	0.37	111	-0.09 [-0.28; 0.10]	0.36	111	-0.06 [-0.25; 0.13]	0.53
	log	mod1	114	-0.02 [-0.21; 0.17]	0.82	114	0.00 [-0.19; 0.19]	>0.99	113	-0.10 [-0.28; 0.08]	0.27	113	-0.06 [-0.24; 0.13]	0.54	111	-0.07 [-0.26; 0.12]	0.45	111	-0.05 [-0.25; 0.14]	0.57
Total lipids in lipoprotein particles		unadj.	114	-0.08 [-0.27; 0.11]	0.40	114	-0.05 [-0.24; 0.14]	0.60	113	-0.15 [-0.34; 0.03]	0.10	113	-0.11 [-0.30; 0.08]	0.25	111	-0.09 [-0.28; 0.10]	0.35	111	-0.07 [-0.26; 0.12]	0.47
		mod1	114	-0.08 [-0.27; 0.11]	0.39	114	-0.06 [-0.24; 0.13]	0.55	113	-0.16 [-0.34; 0.02]	0.073	113	-0.11 [-0.29; 0.07]	0.21	111	-0.09 [-0.28; 0.10]	0.35	111	-0.07 [-0.26; 0.12]	0.49
Total lipids in VLDL	*log*	unadj.	114	-0.02 [-0.21; 0.17]	0.84	114	0.03 [-0.15; 0.22]	0.73	113	-0.09 [-0.27; 0.10]	0.37	113	-0.03 [-0.21; 0.16]	0.78	111	-0.15 [-0.34; 0.04]	0.11	111	-0.16 [-0.34; 0.03]	0.10
	log	mod1	114	-0.04 [-0.22; 0.15]	0.71	114	0.01 [-0.17; 0.20]	0.88	113	-0.13 [-0.30; 0.05]	0.16	113	-0.06 [-0.24; 0.12]	0.51	111	-0.17 [-0.36; 0.02]	0.072	111	-0.17 [-0.36; 0.02]	0.082
Total lipids in LDL	*log*	unadj.	114	-0.04 [-0.22; 0.15]	0.69	114	-0.01 [-0.20; 0.17]	0.89	113	-0.12 [-0.30; 0.07]	0.22	113	-0.08 [-0.27; 0.11]	0.41	111	-0.07 [-0.26; 0.12]	0.44	111	-0.06 [-0.25; 0.13]	0.50
	log	mod1	114	-0.03 [-0.21; 0.16]	0.77	114	0.00 [-0.18; 0.19]	0.99	113	-0.09 [-0.27; 0.09]	0.30	113	-0.06 [-0.24; 0.12]	0.53	111	-0.07 [-0.25; 0.12]	0.50	111	-0.06 [-0.25; 0.13]	0.51
Total lipids in HDL	*log*	unadj.	114	-0.04 [-0.23; 0.14]	0.64	114	-0.03 [-0.21; 0.16]	0.79	113	-0.14 [-0.33; 0.04]	0.13	113	-0.10 [-0.29; 0.09]	0.30	111	-0.09 [-0.27; 0.10]	0.37	111	-0.06 [-0.25; 0.13]	0.52
	log	mod1	114	-0.03 [-0.22; 0.16]	0.76	114	-0.01 [-0.19; 0.18]	0.95	113	-0.11 [-0.28; 0.07]	0.24	113	-0.07 [-0.25; 0.12]	0.47	111	-0.07 [-0.26; 0.12]	0.48	111	-0.05 [-0.25; 0.14]	0.57
Total concentration of lipoprotein		unadj.	114	-0.05 [-0.24; 0.13]	0.56	114	-0.05 [-0.24; 0.14]	0.60	113	-0.15 [-0.34; 0.03]	0.10	113	-0.11 [-0.30; 0.08]	0.24	111	-0.11 [-0.30; 0.08]	0.25	111	-0.07 [-0.26; 0.12]	0.45
particles		mod1	114	-0.05 [-0.24; 0.14]	0.60	114	-0.04 [-0.23; 0.14]	0.64	113	-0.14 [-0.32; 0.04]	0.12	113	-0.10 [-0.28; 0.08]	0.28	111	-0.10 [-0.29; 0.09]	0.28	111	-0.07 [-0.26; 0.12]	0.47
Concentration of VLDL particles	*log*	unadj.	114	-0.04 [-0.22; 0.15]	0.70	114	0.01 [-0.18; 0.20]	0.92	113	-0.10 [-0.29; 0.09]	0.29	113	-0.05 [-0.23; 0.14]	0.62	111	-0.14 [-0.33; 0.05]	0.15	111	-0.13 [-0.32; 0.05]	0.16
	log	mod1	114	-0.05 [-0.23; 0.14]	0.62	114	-0.00 [-0.19; 0.18]	0.99	113	-0.12 [-0.30; 0.05]	0.16	113	-0.07 [-0.25; 0.11]	0.46	111	-0.15 [-0.34; 0.04]	0.11	111	-0.14 [-0.33; 0.05]	0.14
Concentration of LDL particles	*log*	unadj.	114	-0.02 [-0.20; 0.17]	0.86	114	0.01 [-0.18; 0.19]	0.94	113	-0.10 [-0.29; 0.09]	0.29	113	-0.06 [-0.25; 0.13]	0.52	111	-0.10 [-0.28; 0.09]	0.32	111	-0.09 [-0.27; 0.10]	0.38
	log	mod1	114	-0.01 [-0.20; 0.18]	0.91	114	0.02 [-0.17; 0.20]	0.87	113	-0.09 [-0.26; 0.09]	0.33	113	-0.05 [-0.23; 0.13]	0.59	111	-0.09 [-0.28; 0.10]	0.34	111	-0.09 [-0.28; 0.10]	0.37
Concentration of HDL particles	*log*	unadj.	114	-0.02 [-0.21; 0.16]	0.79	114	-0.03 [-0.22; 0.16]	0.75	113	-0.13 [-0.32; 0.06]	0.17	113	-0.08 [-0.27; 0.10]	0.37	111	-0.12 [-0.31; 0.07]	0.22	111	-0.08 [-0.27; 0.11]	0.41
	log	mod1	114	-0.01 [-0.20; 0.17]	0.89	114	-0.01 [-0.20; 0.17]	0.88	113	-0.10 [-0.28; 0.08]	0.26	113	-0.06 [-0.24; 0.12]	0.51	111	-0.11 [-0.30; 0.08]	0.27	111	-0.08 [-0.27; 0.11]	0.42
Average diameter for VLDL particles		unadj.	114	0.03 [-0.16; 0.21]	0.79	114	0.07 [-0.12; 0.25]	0.49	113	-0.04 [-0.22; 0.15]	0.71	113	0.02 [-0.17; 0.21]	0.85	111	-0.17 [-0.36; 0.01]	0.068	111	-0.18 [-0.37; 0.01]	0.057
		mod1	114	0.00 [-0.19; 0.20]	0.96	114	0.05 [-0.14; 0.23]	0.63	113	-0.09 [-0.27; 0.09]	0.33	113	-0.03 [-0.21; 0.16]	0.77	111	-0.21 [-0.40; -0.02]	0.032	111	-0.21 [-0.39; -0.02]	0.034
Average diameter for LDL particles		unadj.	114	0.02 [-0.17; 0.20]	0.85	114	0.02 [-0.16; 0.21]	0.81	113	-0.04 [-0.23; 0.15]	0.66	113	-0.01 [-0.20; 0.17]	0.88	111	-0.04 [-0.23; 0.15]	0.70	111	-0.02 [-0.21; 0.17]	0.85
		mod1	114	0.03 [-0.16; 0.22]	0.75	114	0.04 [-0.15; 0.23]	0.67	113	-0.01 [-0.19; 0.17]	0.89	113	0.01 [-0.17; 0.19]	0.91	111	-0.03 [-0.22; 0.16]	0.78	111	-0.02 [-0.21; 0.17]	0.85
Average diameter for HDL particles		unadj.	114	-0.07 [-0.26; 0.12]	0.46	114	-0.00 [-0.19; 0.18]	0.96	113	-0.11 [-0.30; 0.08]	0.24	113	-0.09 [-0.27; 0.10]	0.37	111	0.02 [-0.17; 0.21]	0.83	111	-0.00 [-0.19; 0.19]	0.97
		mod1	114	-0.05 [-0.24; 0.14]	0.59	114	0.02 [-0.17; 0.21]	0.85	113	-0.06 [-0.24; 0.12]	0.48	113	-0.04 [-0.23; 0.14]	0.63	111	0.05 [-0.15; 0.24]	0.64	111	0.01 [-0.18; 0.20]	0.90
**Detailed lipoprotein contents**																				

			HOMA2-IR (C-peptide)			HOMA2-IR (Insulin)			AUC (C-peptide/Glucose)			AUC (Insulin/Glucose)			oDI (C-peptide)			oDI (Insulin)		
Metabolite		Model	n	Beta ± 95% CI	p-value	n	Beta ± 95% CI	p-value	n	Beta ± 95% CI	p-value	n	Beta ± 95% CI	p-value	n	Beta ± 95% CI	p-value	n	Beta ± 95% CI	p-value
Concentration of XXL-VLDL particles				NA			NA			NA			NA			NA			NA	
Concentration of XL-VLDL particles				NA			NA			NA			NA			NA			NA	
Concentration of L-VLDL particles				NA			NA			NA			NA			NA			NA	
Concentration of M-VLDL particles				NA			NA			NA			NA			NA			NA	
Concentration of S-VLDL particles	*log*	unadj.	114	-0.03 [-0.22; 0.16]	0.76	114	0.00 [-0.18; 0.19]	0.98	113	-0.09 [-0.28; 0.10]	0.34	113	-0.04 [-0.23; 0.15]	0.65	111	-0.15 [-0.34; 0.04]	0.12	111	-0.14 [-0.33; 0.05]	0.15
	log	mod1	114	-0.04 [-0.23; 0.15]	0.66	114	-0.01 [-0.20; 0.18]	0.91	113	-0.12 [-0.30; 0.06]	0.18	113	-0.07 [-0.25; 0.11]	0.45	111	-0.17 [-0.35; 0.02]	0.083	111	-0.15 [-0.34; 0.04]	0.13
Concentration of XS-VLDL particles	*log*	unadj.	114	-0.06 [-0.25; 0.13]	0.53	114	-0.01 [-0.20; 0.17]	0.89	113	-0.12 [-0.31; 0.06]	0.20	113	-0.07 [-0.26; 0.12]	0.45	111	-0.10 [-0.29; 0.08]	0.27	111	-0.10 [-0.29; 0.09]	0.31
	log	mod1	114	-0.06 [-0.25; 0.12]	0.51	114	-0.02 [-0.20; 0.17]	0.85	113	-0.13 [-0.31; 0.05]	0.15	113	-0.08 [-0.26; 0.10]	0.39	111	-0.11 [-0.30; 0.08]	0.26	111	-0.10 [-0.29; 0.09]	0.31
Concentration of IDL particles	*log*	unadj.	114	-0.09 [-0.27; 0.10]	0.36	114	-0.06 [-0.25; 0.12]	0.50	113	-0.15 [-0.33; 0.04]	0.12	113	-0.10 [-0.29; 0.09]	0.28	111	-0.12 [-0.31; 0.07]	0.21	111	-0.06 [-0.25; 0.13]	0.52
	log	mod1	114	-0.08 [-0.26; 0.11]	0.41	114	-0.05 [-0.24; 0.13]	0.59	113	-0.13 [-0.31; 0.05]	0.15	113	-0.08 [-0.27; 0.10]	0.36	111	-0.11 [-0.30; 0.08]	0.24	111	-0.06 [-0.25; 0.13]	0.53
Concentration of L-LDL particles	*log*	unadj.	114	-0.00 [-0.19; 0.18]	0.98	114	0.02 [-0.17; 0.21]	0.83	113	-0.07 [-0.26; 0.12]	0.46	113	-0.04 [-0.23; 0.15]	0.67	111	-0.03 [-0.22; 0.16]	0.78	111	-0.03 [-0.22; 0.16]	0.75
	log	mod1	114	0.01 [-0.18; 0.20]	0.92	114	0.04 [-0.15; 0.22]	0.70	113	-0.04 [-0.22; 0.14]	0.65	113	-0.02 [-0.20; 0.17]	0.87	111	-0.01 [-0.20; 0.18]	0.88	111	-0.03 [-0.22; 0.17]	0.79
Concentration of M-LDL particles	*log*	unadj.	114	-0.01 [-0.20; 0.17]	0.90	114	0.02 [-0.17; 0.21]	0.85	113	-0.08 [-0.27; 0.10]	0.37	113	-0.04 [-0.23; 0.14]	0.65	111	-0.06 [-0.25; 0.13]	0.55	111	-0.06 [-0.25; 0.13]	0.51
	log	mod1	114	-0.01 [-0.20; 0.18]	0.94	114	0.03 [-0.16; 0.21]	0.78	113	-0.07 [-0.25; 0.10]	0.42	113	-0.03 [-0.22; 0.15]	0.71	111	-0.05 [-0.24; 0.13]	0.57	111	-0.07 [-0.26; 0.12]	0.49
Concentration of S-LDL particles	*log*	unadj.	114	-0.09 [-0.28; 0.09]	0.33	114	-0.07 [-0.26; 0.12]	0.46	113	-0.15 [-0.34; 0.04]	0.11	113	-0.12 [-0.31; 0.07]	0.20	111	-0.06 [-0.25; 0.13]	0.51	111	-0.04 [-0.23; 0.15]	0.66
	log	mod1	114	-0.09 [-0.28; 0.09]	0.33	114	-0.07 [-0.25; 0.12]	0.47	113	-0.15 [-0.32; 0.03]	0.10	113	-0.12 [-0.30; 0.06]	0.20	111	-0.06 [-0.25; 0.13]	0.51	111	-0.04 [-0.23; 0.15]	0.65
Concentration of XL-HDL particles	*log*	unadj.	114	-0.07 [-0.25; 0.12]	0.48	114	-0.01 [-0.19; 0.18]	0.95	113	-0.16 [-0.34; 0.03]	0.10	113	-0.12 [-0.31; 0.07]	0.21	111	-0.03 [-0.22; 0.16]	0.78	111	-0.05 [-0.24; 0.14]	0.61
	log	mod1	114	-0.05 [-0.24; 0.14]	0.60	114	0.01 [-0.18; 0.20]	0.89	113	-0.11 [-0.29; 0.07]	0.21	113	-0.08 [-0.27; 0.10]	0.37	111	-0.00 [-0.20; 0.19]	0.97	111	-0.03 [-0.23; 0.16]	0.73
Concentration of L-HDL particles	*log*	unadj.	114	-0.03 [-0.21; 0.16]	0.78	114	0.00 [-0.18; 0.19]	0.97	113	-0.17 [-0.35; 0.02]	0.080	113	-0.14 [-0.33; 0.05]	0.14	111	-0.05 [-0.24; 0.14]	0.62	111	-0.05 [-0.24; 0.13]	0.57
	log	mod1	114	-0.00 [-0.19; 0.19]	0.98	114	0.03 [-0.16; 0.22]	0.75	113	-0.11 [-0.29; 0.07]	0.22	113	-0.09 [-0.28; 0.09]	0.32	111	-0.02 [-0.21; 0.17]	0.84	111	-0.04 [-0.23; 0.16]	0.70
Concentration of M-HDL particles	*log*	unadj.	114	-0.03 [-0.22; 0.16]	0.76	114	-0.04 [-0.22; 0.15]	0.71	113	-0.12 [-0.31; 0.07]	0.20	113	-0.08 [-0.26; 0.11]	0.42	111	-0.10 [-0.29; 0.09]	0.31	111	-0.05 [-0.24; 0.14]	0.58
	log	mod1	114	-0.01 [-0.20; 0.17]	0.89	114	-0.02 [-0.20; 0.17]	0.87	113	-0.09 [-0.26; 0.09]	0.35	113	-0.05 [-0.23; 0.14]	0.62	111	-0.08 [-0.27; 0.11]	0.40	111	-0.05 [-0.24; 0.14]	0.60
Concentration of S-HDL particles	*log*	unadj.	114	0.01 [-0.18; 0.20]	0.90	114	-0.02 [-0.21; 0.16]	0.80	113	-0.09 [-0.27; 0.10]	0.37	113	-0.05 [-0.24; 0.14]	0.59	111	-0.13 [-0.32; 0.05]	0.16	111	-0.08 [-0.27; 0.11]	0.38
	log	mod1	114	0.01 [-0.17; 0.20]	0.88	114	-0.02 [-0.20; 0.17]	0.85	113	-0.08 [-0.26; 0.10]	0.38	113	-0.05 [-0.23; 0.13]	0.61	111	-0.13 [-0.32; 0.05]	0.16	111	-0.09 [-0.28; 0.10]	0.36
Total lipids in XXL-VLDL				NA			NA			NA			NA			NA			NA	
Total lipids in XL-VLDL				NA			NA			NA			NA			NA			NA	
Total lipids in L-VLDL				NA			NA			NA			NA			NA			NA	
Total lipids in M-VLDL				NA			NA			NA			NA			NA			NA	
Total lipids in S-VLDL	*log*	unadj.	114	-0.01 [-0.20; 0.17]	0.89	114	0.02 [-0.17; 0.21]	0.82	113	-0.08 [-0.27; 0.10]	0.38	113	-0.03 [-0.22; 0.15]	0.73	111	-0.15 [-0.34; 0.04]	0.12	111	-0.14 [-0.33; 0.05]	0.14
	log	mod1	114	-0.02 [-0.21; 0.16]	0.79	114	0.01 [-0.18; 0.20]	0.92	113	-0.11 [-0.29; 0.07]	0.22	113	-0.06 [-0.24; 0.13]	0.54	111	-0.16 [-0.35; 0.02]	0.087	111	-0.15 [-0.34; 0.04]	0.12
Total lipids in XS-VLDL	*log*	unadj.	114	-0.06 [-0.24; 0.13]	0.55	114	-0.00 [-0.19; 0.19]	0.99	113	-0.11 [-0.29; 0.08]	0.27	113	-0.06 [-0.24; 0.13]	0.55	111	-0.10 [-0.29; 0.09]	0.29	111	-0.10 [-0.29; 0.09]	0.29
	log	mod1	114	-0.06 [-0.25; 0.12]	0.50	114	-0.01 [-0.20; 0.17]	0.89	113	-0.12 [-0.30; 0.05]	0.17	113	-0.07 [-0.25; 0.11]	0.43	111	-0.11 [-0.30; 0.08]	0.26	111	-0.10 [-0.29; 0.09]	0.30
Total lipids in IDL	*log*	unadj.	114	-0.05 [-0.23; 0.14]	0.62	114	-0.01 [-0.20; 0.18]	0.93	113	-0.14 [-0.32; 0.05]	0.15	113	-0.09 [-0.28; 0.09]	0.33	111	-0.11 [-0.29; 0.08]	0.27	111	-0.10 [-0.29; 0.09]	0.29
	log	mod1	114	-0.05 [-0.24; 0.14]	0.61	114	-0.01 [-0.20; 0.18]	0.89	113	-0.14 [-0.32; 0.04]	0.12	113	-0.09 [-0.28; 0.09]	0.30	111	-0.11 [-0.29; 0.08]	0.27	111	-0.10 [-0.29; 0.09]	0.31
Total lipids in L-LDL	*log*	unadj.	114	-0.01 [-0.19; 0.18]	0.94	114	0.01 [-0.17; 0.20]	0.88	113	-0.09 [-0.28; 0.10]	0.34	113	-0.06 [-0.25; 0.13]	0.55	111	-0.04 [-0.23; 0.15]	0.70	111	-0.04 [-0.23; 0.15]	0.69
	log	mod1	114	0.01 [-0.18; 0.20]	0.93	114	0.03 [-0.15; 0.22]	0.72	113	-0.06 [-0.24; 0.12]	0.53	113	-0.03 [-0.21; 0.15]	0.77	111	-0.02 [-0.21; 0.17]	0.82	111	-0.03 [-0.22; 0.16]	0.74
Total lipids in M-LDL	*log*	unadj.	114	-0.02 [-0.20; 0.17]	0.85	114	0.01 [-0.18; 0.20]	0.90	113	-0.09 [-0.28; 0.10]	0.33	113	-0.05 [-0.24; 0.14]	0.59	111	-0.06 [-0.25; 0.13]	0.55	111	-0.06 [-0.25; 0.13]	0.52
	log	mod1	114	-0.01 [-0.20; 0.18]	0.90	114	0.02 [-0.16; 0.21]	0.82	113	-0.08 [-0.26; 0.10]	0.39	113	-0.04 [-0.22; 0.14]	0.68	111	-0.05 [-0.24; 0.14]	0.58	111	-0.06 [-0.25; 0.13]	0.51
Total lipids in S-LDL	*log*	unadj.	114	-0.10 [-0.28; 0.09]	0.31	114	-0.08 [-0.26; 0.11]	0.42	113	-0.15 [-0.34; 0.04]	0.11	113	-0.12 [-0.31; 0.07]	0.20	111	-0.06 [-0.25; 0.13]	0.52	111	-0.04 [-0.23; 0.15]	0.71
	log	mod1	114	-0.09 [-0.28; 0.09]	0.32	114	-0.07 [-0.26; 0.11]	0.44	113	-0.15 [-0.32; 0.03]	0.10	113	-0.12 [-0.30; 0.06]	0.21	111	-0.06 [-0.25; 0.13]	0.52	111	-0.04 [-0.23; 0.15]	0.69
Total lipids in XL-HDL	*log*	unadj.	114	-0.07 [-0.25; 0.12]	0.48	114	-0.01 [-0.19; 0.18]	0.94	113	-0.14 [-0.33; 0.04]	0.14	113	-0.11 [-0.30; 0.07]	0.23	111	-0.01 [-0.20; 0.18]	0.95	111	-0.03 [-0.22; 0.16]	0.74
	log	mod1	114	-0.05 [-0.24; 0.14]	0.61	114	0.01 [-0.18; 0.20]	0.89	113	-0.10 [-0.28; 0.08]	0.28	113	-0.08 [-0.26; 0.11]	0.42	111	0.02 [-0.17; 0.21]	0.85	111	-0.01 [-0.21; 0.18]	0.88
Total lipids in L-HDL	*log*	unadj.	114	-0.04 [-0.23; 0.15]	0.68	114	-0.01 [-0.19; 0.18]	0.95	113	-0.16 [-0.34; 0.03]	0.10	113	-0.13 [-0.31; 0.06]	0.18	111	-0.04 [-0.23; 0.15]	0.68	111	-0.04 [-0.23; 0.15]	0.65
	log	mod1	114	-0.02 [-0.21; 0.17]	0.87	114	0.02 [-0.17; 0.21]	0.82	113	-0.10 [-0.28; 0.08]	0.27	113	-0.08 [-0.26; 0.10]	0.39	111	-0.01 [-0.20; 0.18]	0.90	111	-0.03 [-0.22; 0.17]	0.78
Total lipids in M-HDL	*log*	unadj.	114	-0.02 [-0.21; 0.16]	0.80	114	-0.04 [-0.22; 0.15]	0.70	113	-0.11 [-0.30; 0.08]	0.25	113	-0.07 [-0.26; 0.12]	0.47	111	-0.10 [-0.29; 0.09]	0.31	111	-0.05 [-0.24; 0.14]	0.61
	log	mod1	114	-0.01 [-0.20; 0.18]	0.91	114	-0.02 [-0.21; 0.17]	0.83	113	-0.08 [-0.26; 0.10]	0.38	113	-0.04 [-0.23; 0.14]	0.64	111	-0.08 [-0.27; 0.11]	0.38	111	-0.05 [-0.24; 0.14]	0.62
Total lipids in S-HDL	*log*	unadj.	114	0.01 [-0.18; 0.19]	0.95	114	-0.03 [-0.21; 0.16]	0.79	113	-0.07 [-0.26; 0.11]	0.44	113	-0.04 [-0.22; 0.15]	0.70	111	-0.13 [-0.32; 0.06]	0.18	111	-0.07 [-0.26; 0.12]	0.45
	log	mod1	114	0.00 [-0.18; 0.19]	0.97	114	-0.03 [-0.21; 0.16]	0.78	113	-0.08 [-0.26; 0.10]	0.38	113	-0.04 [-0.22; 0.14]	0.64	111	-0.13 [-0.32; 0.06]	0.17	111	-0.08 [-0.27; 0.11]	0.41
Phospholipids in XXL-VLDL				NA			NA			NA			NA			NA			NA	
Cholesterol in XXL-VLDL				NA			NA			NA			NA			NA			NA	
Triglycerides in XXL-VLDL				NA			NA			NA			NA			NA			NA	
Phospholipids in XL-VLDL				NA			NA			NA			NA			NA			NA	
Cholesterol in XL-VLDL				NA			NA			NA			NA			NA			NA	
Triglycerides in XL-VLDL				NA			NA			NA			NA			NA			NA	
Phospholipids in L-VLDL				NA			NA			NA			NA			NA			NA	
Cholesterol in L-VLDL				NA			NA			NA			NA			NA			NA	
Triglycerides in L-VLDL				NA			NA			NA			NA			NA			NA	
Phospholipids in M-VLDL				NA			NA			NA			NA			NA			NA	
Cholesterol in M-VLDL				NA			NA			NA			NA			NA			NA	
Triglycerides in M-VLDL				NA			NA			NA			NA			NA			NA	

			HOMA2-IR (C-peptide)			HOMA2-IR (Insulin)			AUC (C-peptide/Glucose)			AUC (Insulin/Glucose)			oDI (C-peptide)			oDI (Insulin)		
Metabolite		Model	n	Beta ± 95% CI	p-value	n	Beta ± 95% CI	p-value	n	Beta ± 95% CI	p-value	n	Beta ± 95% CI	p-value	n	Beta ± 95% CI	p-value	n	Beta ± 95% CI	p-value
Phospholipids in S-VLDL	*log*	unadj.	114	0.00 [-0.19; 0.19]	>0.99	114	0.03 [-0.16; 0.21]	0.78	113	-0.08 [-0.27; 0.11]	0.39	113	-0.04 [-0.22; 0.15]	0.70	111	-0.15 [-0.34; 0.04]	0.12	111	-0.14 [-0.33; 0.05]	0.15
	log	mod1	114	-0.01 [-0.19; 0.18]	0.95	114	0.02 [-0.17; 0.21]	0.83	113	-0.10 [-0.27; 0.08]	0.28	113	-0.05 [-0.23; 0.13]	0.59	111	-0.16 [-0.34; 0.03]	0.098	111	-0.14 [-0.33; 0.04]	0.13
Cholesterol in S-VLDL	*log*	unadj.	114	0.00 [-0.18; 0.19]	0.97	114	0.03 [-0.16; 0.21]	0.78	113	-0.08 [-0.27; 0.11]	0.41	113	-0.04 [-0.23; 0.15]	0.69	111	-0.15 [-0.34; 0.04]	0.12	111	-0.14 [-0.33; 0.05]	0.14
	log	mod1	114	-0.01 [-0.19; 0.18]	0.95	114	0.02 [-0.17; 0.20]	0.86	113	-0.10 [-0.28; 0.08]	0.26	113	-0.06 [-0.24; 0.12]	0.53	111	-0.16 [-0.35; 0.03]	0.093	111	-0.15 [-0.34; 0.04]	0.13
Triglycerides in S-VLDL	*log*	unadj.	114	-0.04 [-0.22; 0.15]	0.71	114	0.01 [-0.18; 0.20]	0.91	113	-0.07 [-0.26; 0.11]	0.43	113	-0.02 [-0.20; 0.17]	0.87	111	-0.13 [-0.32; 0.05]	0.16	111	-0.12 [-0.31; 0.06]	0.19
	log	mod1	114	-0.05 [-0.24; 0.14]	0.59	114	-0.01 [-0.19; 0.18]	0.95	113	-0.11 [-0.29; 0.07]	0.21	113	-0.05 [-0.23; 0.14]	0.61	111	-0.15 [-0.34; 0.03]	0.11	111	-0.14 [-0.33; 0.05]	0.16
Phospholipids in XS-VLDL	*log*	unadj.	114	-0.06 [-0.25; 0.12]	0.51	114	-0.01 [-0.20; 0.18]	0.92	113	-0.11 [-0.29; 0.08]	0.26	113	-0.06 [-0.25; 0.13]	0.54	111	-0.10 [-0.28; 0.09]	0.32	111	-0.09 [-0.28; 0.09]	0.32
	log	mod1	114	-0.07 [-0.26; 0.12]	0.46	114	-0.02 [-0.21; 0.17]	0.83	113	-0.13 [-0.30; 0.05]	0.16	113	-0.07 [-0.26; 0.11]	0.42	111	-0.10 [-0.29; 0.09]	0.28	111	-0.10 [-0.29; 0.09]	0.32
Cholesterol in XS-VLDL	*log*	unadj.	114	-0.05 [-0.23; 0.14]	0.63	114	0.01 [-0.17; 0.20]	0.89	113	-0.10 [-0.29; 0.09]	0.29	113	-0.05 [-0.24; 0.13]	0.57	111	-0.09 [-0.28; 0.10]	0.34	111	-0.10 [-0.29; 0.09]	0.31
	log	mod1	114	-0.05 [-0.24; 0.14]	0.58	114	0.00 [-0.18; 0.19]	0.98	113	-0.12 [-0.29; 0.06]	0.19	113	-0.07 [-0.25; 0.11]	0.46	111	-0.10 [-0.29; 0.09]	0.31	111	-0.10 [-0.29; 0.09]	0.32
Triglycerides in XS-VLDL	*log*	unadj.	114	-0.07 [-0.26; 0.12]	0.47	114	-0.03 [-0.22; 0.15]	0.72	113	-0.11 [-0.29; 0.08]	0.26	113	-0.06 [-0.25; 0.13]	0.52	111	-0.12 [-0.31; 0.07]	0.21	111	-0.10 [-0.29; 0.09]	0.30
	log	mod1	114	-0.08 [-0.27; 0.11]	0.40	114	-0.05 [-0.23; 0.14]	0.62	113	-0.13 [-0.31; 0.04]	0.14	113	-0.08 [-0.26; 0.10]	0.36	111	-0.13 [-0.32; 0.06]	0.17	111	-0.11 [-0.30; 0.08]	0.28
Phospholipids in IDL	*log*	unadj.	114	-0.05 [-0.23; 0.14]	0.63	114	-0.00 [-0.19; 0.18]	0.98	113	-0.14 [-0.32; 0.05]	0.15	113	-0.09 [-0.28; 0.09]	0.32	111	-0.08 [-0.27; 0.11]	0.42	111	-0.08 [-0.27; 0.11]	0.40
	log	mod1	114	-0.04 [-0.22; 0.15]	0.69	114	0.01 [-0.18; 0.19]	0.95	113	-0.12 [-0.30; 0.06]	0.19	113	-0.08 [-0.26; 0.10]	0.40	111	-0.07 [-0.26; 0.12]	0.48	111	-0.07 [-0.27; 0.12]	0.44
Cholesterol in IDL	*log*	unadj.	114	-0.04 [-0.23; 0.14]	0.64	114	-0.01 [-0.20; 0.18]	0.92	113	-0.13 [-0.32; 0.05]	0.16	113	-0.09 [-0.28; 0.10]	0.34	111	-0.11 [-0.30; 0.08]	0.25	111	-0.10 [-0.29; 0.09]	0.29
	log	mod1	114	-0.05 [-0.24; 0.14]	0.61	114	-0.02 [-0.20; 0.17]	0.85	113	-0.14 [-0.32; 0.03]	0.11	113	-0.10 [-0.28; 0.08]	0.27	111	-0.11 [-0.30; 0.08]	0.24	111	-0.10 [-0.29; 0.09]	0.31
Triglycerides in IDL	*log*	unadj.	114	-0.08 [-0.26; 0.11]	0.42	114	-0.04 [-0.22; 0.15]	0.70	113	-0.13 [-0.32; 0.06]	0.17	113	-0.08 [-0.27; 0.10]	0.38	111	-0.11 [-0.30; 0.08]	0.26	111	-0.09 [-0.28; 0.10]	0.33
	log	mod1	114	-0.08 [-0.27; 0.11]	0.39	114	-0.04 [-0.23; 0.14]	0.66	113	-0.14 [-0.32; 0.04]	0.12	113	-0.09 [-0.27; 0.09]	0.32	111	-0.11 [-0.30; 0.08]	0.24	111	-0.09 [-0.28; 0.10]	0.32
Phospholipids in L-LDL	*log*	unadj.	114	-0.02 [-0.21; 0.17]	0.83	114	0.00 [-0.19; 0.19]	>0.99	113	-0.11 [-0.30; 0.07]	0.23	113	-0.08 [-0.26; 0.11]	0.43	111	-0.04 [-0.23; 0.15]	0.64	111	-0.04 [-0.23; 0.15]	0.66
	log	mod1	114	-0.01 [-0.20; 0.18]	0.91	114	0.01 [-0.17; 0.20]	0.90	113	-0.09 [-0.27; 0.09]	0.31	113	-0.06 [-0.24; 0.13]	0.54	111	-0.03 [-0.22; 0.16]	0.72	111	-0.04 [-0.23; 0.15]	0.69
Cholesterol in L-LDL	*log*	unadj.	114	-0.00 [-0.19; 0.19]	>0.99	114	0.02 [-0.17; 0.21]	0.85	113	-0.08 [-0.26; 0.11]	0.42	113	-0.05 [-0.23; 0.14]	0.63	111	-0.03 [-0.21; 0.16]	0.79	111	-0.03 [-0.22; 0.16]	0.79
	log	mod1	114	0.02 [-0.17; 0.21]	0.85	114	0.04 [-0.15; 0.23]	0.66	113	-0.03 [-0.21; 0.14]	0.70	113	-0.01 [-0.19; 0.17]	0.92	111	-0.01 [-0.20; 0.18]	0.94	111	-0.02 [-0.21; 0.17]	0.84
Triglycerides in L-LDL	*log*	unadj.	114	-0.06 [-0.24; 0.13]	0.56	114	-0.03 [-0.22; 0.15]	0.72	113	-0.11 [-0.30; 0.07]	0.23	113	-0.08 [-0.26; 0.11]	0.42	111	-0.01 [-0.20; 0.18]	0.91	111	0.00 [-0.19; 0.19]	0.97
	log	mod1	114	-0.05 [-0.24; 0.14]	0.61	114	-0.02 [-0.21; 0.16]	0.79	113	-0.10 [-0.27; 0.08]	0.29	113	-0.06 [-0.24; 0.12]	0.51	111	-0.00 [-0.19; 0.19]	0.98	111	0.01 [-0.18; 0.20]	0.93
Phospholipids in M-LDL	*log*	unadj.	114	-0.03 [-0.21; 0.16]	0.78	114	0.00 [-0.18; 0.19]	0.98	113	-0.11 [-0.30; 0.08]	0.24	113	-0.07 [-0.25; 0.12]	0.48	111	-0.06 [-0.25; 0.13]	0.54	111	-0.06 [-0.25; 0.13]	0.51
	log	mod1	114	-0.02 [-0.21; 0.16]	0.81	114	0.01 [-0.18; 0.19]	0.93	113	-0.10 [-0.28; 0.08]	0.26	113	-0.06 [-0.24; 0.12]	0.51	111	-0.06 [-0.25; 0.13]	0.56	111	-0.07 [-0.26; 0.13]	0.50
Cholesterol in M-LDL	*log*	unadj.	114	-0.01 [-0.20; 0.18]	0.91	114	0.02 [-0.17; 0.20]	0.87	113	-0.08 [-0.27; 0.11]	0.41	113	-0.04 [-0.23; 0.15]	0.68	111	-0.05 [-0.24; 0.14]	0.60	111	-0.05 [-0.24; 0.14]	0.58
	log	mod1	114	-0.00 [-0.19; 0.19]	0.98	114	0.03 [-0.16; 0.21]	0.76	113	-0.06 [-0.24; 0.12]	0.53	113	-0.02 [-0.20; 0.16]	0.81	111	-0.04 [-0.23; 0.15]	0.65	111	-0.05 [-0.25; 0.14]	0.57
Triglycerides in M-LDL	*log*	unadj.	114	-0.06 [-0.25; 0.13]	0.52	114	-0.03 [-0.21; 0.16]	0.78	113	-0.12 [-0.30; 0.07]	0.22	113	-0.07 [-0.26; 0.12]	0.46	111	-0.03 [-0.22; 0.16]	0.79	111	-0.02 [-0.21; 0.17]	0.83
	log	mod1	114	-0.06 [-0.25; 0.13]	0.52	114	-0.02 [-0.21; 0.16]	0.80	113	-0.12 [-0.29; 0.06]	0.20	113	-0.07 [-0.25; 0.11]	0.44	111	-0.03 [-0.22; 0.16]	0.78	111	-0.02 [-0.21; 0.17]	0.82
Phospholipids in S-LDL	*log*	unadj.	114	-0.13 [-0.31; 0.06]	0.17	114	-0.11 [-0.30; 0.07]	0.22	113	-0.15 [-0.34; 0.03]	0.10	113	-0.13 [-0.32; 0.05]	0.16	111	-0.04 [-0.23; 0.15]	0.65	111	-0.00 [-0.19; 0.19]	0.97
	log	mod1	114	-0.13 [-0.31; 0.06]	0.17	114	-0.11 [-0.30; 0.07]	0.23	113	-0.15 [-0.33; 0.02]	0.091	113	-0.13 [-0.31; 0.05]	0.15	111	-0.04 [-0.23; 0.15]	0.66	111	-0.01 [-0.20; 0.19]	0.96
Cholesterol in S-LDL	*log*	unadj.	114	-0.08 [-0.27; 0.10]	0.37	114	-0.07 [-0.25; 0.12]	0.48	113	-0.15 [-0.33; 0.04]	0.12	113	-0.11 [-0.30; 0.07]	0.23	111	-0.06 [-0.25; 0.13]	0.53	111	-0.04 [-0.23; 0.15]	0.69
	log	mod1	114	-0.08 [-0.27; 0.11]	0.39	114	-0.06 [-0.24; 0.13]	0.53	113	-0.14 [-0.31; 0.04]	0.13	113	-0.11 [-0.29; 0.07]	0.25	111	-0.06 [-0.25; 0.13]	0.54	111	-0.04 [-0.23; 0.15]	0.68
Triglycerides in S-LDL	*log*	unadj.	114	-0.12 [-0.30; 0.07]	0.21	114	-0.10 [-0.29; 0.09]	0.29	113	-0.15 [-0.33; 0.04]	0.12	113	-0.11 [-0.30; 0.07]	0.23	111	-0.07 [-0.26; 0.12]	0.46	111	-0.03 [-0.22; 0.16]	0.73
	log	mod1	114	-0.13 [-0.31; 0.06]	0.19	114	-0.10 [-0.29; 0.08]	0.27	113	-0.16 [-0.34; 0.01]	0.068	113	-0.13 [-0.31; 0.05]	0.17	111	-0.08 [-0.27; 0.11]	0.41	111	-0.04 [-0.23; 0.15]	0.66
Phospholipids in XL-HDL	*log*	unadj.	114	-0.06 [-0.25; 0.13]	0.53	114	-0.00 [-0.19; 0.19]	0.99	113	-0.14 [-0.32; 0.05]	0.15	113	-0.11 [-0.30; 0.08]	0.25	111	-0.01 [-0.20; 0.18]	0.95	111	-0.03 [-0.22; 0.16]	0.75
	log	mod1	114	-0.04 [-0.23; 0.15]	0.67	114	0.02 [-0.17; 0.21]	0.84	113	-0.09 [-0.27; 0.09]	0.31	113	-0.07 [-0.25; 0.11]	0.45	111	0.02 [-0.17; 0.21]	0.84	111	-0.01 [-0.21; 0.18]	0.89
Cholesterol in XL-HDL	*log*	unadj.	114	-0.07 [-0.26; 0.12]	0.45	114	-0.01 [-0.20; 0.17]	0.89	113	-0.14 [-0.33; 0.04]	0.13	113	-0.12 [-0.30; 0.07]	0.22	111	0.00 [-0.19; 0.19]	0.99	111	-0.03 [-0.22; 0.16]	0.78
	log	mod1	114	-0.05 [-0.24; 0.14]	0.58	114	0.01 [-0.18; 0.20]	0.94	113	-0.10 [-0.28; 0.08]	0.28	113	-0.08 [-0.26; 0.10]	0.39	111	0.03 [-0.17; 0.22]	0.79	111	-0.01 [-0.20; 0.18]	0.92
Triglycerides in XL-HDL	*log*	unadj.	114	-0.07 [-0.26; 0.12]	0.46	114	0.01 [-0.18; 0.20]	0.92	113	-0.14 [-0.32; 0.05]	0.15	113	-0.07 [-0.25; 0.12]	0.49	111	-0.10 [-0.29; 0.09]	0.30	111	-0.11 [-0.30; 0.08]	0.26
	log	mod1	114	-0.06 [-0.25; 0.12]	0.50	114	0.01 [-0.17; 0.20]	0.88	113	-0.12 [-0.30; 0.05]	0.17	113	-0.06 [-0.24; 0.13]	0.55	111	-0.09 [-0.28; 0.09]	0.33	111	-0.10 [-0.29; 0.08]	0.28
Phospholipids in L-HDL	*log*	unadj.	114	-0.03 [-0.22; 0.16]	0.75	114	0.00 [-0.18; 0.19]	0.98	113	-0.14 [-0.33; 0.04]	0.13	113	-0.11 [-0.30; 0.08]	0.25	111	-0.04 [-0.23; 0.14]	0.64	111	-0.04 [-0.23; 0.14]	0.64
	log	mod1	114	-0.01 [-0.20; 0.18]	0.93	114	0.03 [-0.16; 0.22]	0.77	113	-0.09 [-0.27; 0.09]	0.31	113	-0.07 [-0.25; 0.12]	0.48	111	-0.02 [-0.21; 0.17]	0.85	111	-0.03 [-0.22; 0.16]	0.76
Cholesterol in L-HDL	*log*	unadj.	114	-0.04 [-0.23; 0.14]	0.65	114	-0.01 [-0.20; 0.17]	0.88	113	-0.17 [-0.35; 0.02]	0.079	113	-0.15 [-0.33; 0.04]	0.12	111	-0.03 [-0.22; 0.16]	0.75	111	-0.04 [-0.23; 0.15]	0.69
	log	mod1	114	-0.02 [-0.21; 0.17]	0.85	114	0.01 [-0.18; 0.20]	0.89	113	-0.11 [-0.29; 0.07]	0.24	113	-0.10 [-0.28; 0.09]	0.30	111	0.00 [-0.19; 0.19]	>0.99	111	-0.02 [-0.21; 0.17]	0.84
Triglycerides in L-HDL	*log*	unadj.	114	-0.03 [-0.22; 0.15]	0.72	114	0.04 [-0.15; 0.23]	0.66	113	-0.09 [-0.28; 0.09]	0.33	113	-0.01 [-0.20; 0.18]	0.90	111	-0.09 [-0.28; 0.10]	0.34	111	-0.09 [-0.28; 0.10]	0.36
	log	mod1	114	-0.03 [-0.21; 0.16]	0.78	114	0.05 [-0.14; 0.24]	0.60	113	-0.08 [-0.25; 0.10]	0.40	113	0.00 [-0.18; 0.18]	0.98	111	-0.08 [-0.27; 0.11]	0.39	111	-0.08 [-0.27; 0.11]	0.39
Phospholipids in M-HDL	*log*	unadj.	114	-0.01 [-0.20; 0.17]	0.88	114	-0.02 [-0.21; 0.16]	0.81	113	-0.10 [-0.29; 0.09]	0.29	113	-0.06 [-0.24; 0.13]	0.55	111	-0.10 [-0.29; 0.09]	0.30	111	-0.05 [-0.24; 0.14]	0.58
	log	mod1	114	-0.01 [-0.19; 0.18]	0.95	114	-0.01 [-0.20; 0.18]	0.91	113	-0.08 [-0.26; 0.10]	0.39	113	-0.04 [-0.22; 0.14]	0.68	111	-0.09 [-0.28; 0.10]	0.35	111	-0.05 [-0.24; 0.14]	0.59
Cholesterol in M-HDL	*log*	unadj.	114	-0.03 [-0.21; 0.16]	0.78	114	-0.05 [-0.24; 0.13]	0.58	113	-0.12 [-0.30; 0.07]	0.22	113	-0.09 [-0.27; 0.10]	0.37	111	-0.08 [-0.27; 0.10]	0.38	111	-0.04 [-0.23; 0.15]	0.71
	log	mod1	114	-0.01 [-0.20; 0.18]	0.93	114	-0.03 [-0.22; 0.16]	0.77	113	-0.07 [-0.25; 0.11]	0.43	113	-0.05 [-0.23; 0.14]	0.61	111	-0.07 [-0.26; 0.13]	0.50	111	-0.03 [-0.23; 0.16]	0.74
Triglycerides in M-HDL	*log*	unadj.	114	-0.02 [-0.21; 0.16]	0.79	114	0.01 [-0.18; 0.20]	0.93	113	-0.06 [-0.25; 0.12]	0.50	113	-0.01 [-0.19; 0.18]	0.95	111	-0.12 [-0.31; 0.07]	0.22	111	-0.09 [-0.28; 0.10]	0.34
	log	mod1	114	-0.04 [-0.23; 0.15]	0.68	114	-0.01 [-0.19; 0.18]	0.94	113	-0.10 [-0.28; 0.08]	0.27	113	-0.03 [-0.22; 0.15]	0.71	111	-0.13 [-0.32; 0.06]	0.16	111	-0.10 [-0.29; 0.09]	0.30
Phospholipids in S-HDL	*log*	unadj.	114	0.00 [-0.18; 0.19]	0.97	114	-0.02 [-0.21; 0.16]	0.80	113	-0.07 [-0.26; 0.12]	0.45	113	-0.03 [-0.22; 0.15]	0.72	111	-0.12 [-0.31; 0.07]	0.20	111	-0.07 [-0.26; 0.12]	0.49
	log	mod1	114	0.00 [-0.19; 0.19]	0.99	114	-0.02 [-0.21; 0.16]	0.80	113	-0.08 [-0.26; 0.10]	0.39	113	-0.04 [-0.22; 0.14]	0.67	111	-0.13 [-0.31; 0.06]	0.18	111	-0.07 [-0.26; 0.12]	0.45
Cholesterol in S-HDL	*log*	unadj.	114	0.02 [-0.17; 0.21]	0.83	114	-0.03 [-0.21; 0.16]	0.78	113	-0.07 [-0.26; 0.12]	0.47	113	-0.04 [-0.23; 0.15]	0.68	111	-0.13 [-0.32; 0.06]	0.18	111	-0.07 [-0.26; 0.12]	0.45
	log	mod1	114	0.02 [-0.17; 0.21]	0.83	114	-0.02 [-0.21; 0.16]	0.80	113	-0.07 [-0.24; 0.11]	0.46	113	-0.04 [-0.22; 0.14]	0.67	111	-0.13 [-0.32; 0.06]	0.17	111	-0.08 [-0.27; 0.11]	0.41
Triglycerides in S-HDL	*log*	unadj.	114	-0.03 [-0.22; 0.16]	0.74	114	-0.02 [-0.20; 0.17]	0.86	113	-0.08 [-0.26; 0.11]	0.42	113	-0.03 [-0.22; 0.16]	0.74	111	-0.12 [-0.31; 0.07]	0.20	111	-0.09 [-0.28; 0.10]	0.34
	log	mod1	114	-0.05 [-0.24; 0.14]	0.61	114	-0.04 [-0.22; 0.15]	0.69	113	-0.12 [-0.30; 0.06]	0.18	113	-0.07 [-0.25; 0.11]	0.46	111	-0.14 [-0.33; 0.05]	0.14	111	-0.10 [-0.29; 0.09]	0.29

		HOMA2-IR (C-peptide)			HOMA2-IR (Insulin)			AUC (C-peptide/Glucose)			AUC (Insulin/Glucose)			oDI (C-peptide)			oDI (Insulin)		
**Metabolite**	**Model**	**n**	**Beta ± 95% CI**	**p-value**	**n**	**Beta ± 95% CI**	**p-value**	**n**	**Beta ± 95% CI**	**p-value**	**n**	**Beta ± 95% CI**	**p-value**	**n**	**Beta ± 95% CI**	**p-value**	**n**	**Beta ± 95% CI**	**p-value**
**Apolipoproteins**																			
Apolipoprotein B	unadj.	114	-0.08 [-0.27; 0.11]	0.40	114	-0.05 [-0.24; 0.13]	0.57	113	-0.16 [-0.35; 0.02]	0.089	113	-0.12 [-0.30; 0.07]	0.21	111	-0.09 [-0.28; 0.10]	0.36	111	-0.07 [-0.26; 0.12]	0.46
	mod1	114	-0.08 [-0.27; 0.10]	0.39	114	-0.06 [-0.24; 0.13]	0.53	113	-0.17 [-0.34; 0.01]	0.064	113	-0.12 [-0.30; 0.06]	0.18	111	-0.09 [-0.28; 0.10]	0.36	111	-0.07 [-0.26; 0.12]	0.49
Apolipoprotein A1	unadj.	114	-0.06 [-0.24; 0.13]	0.56	114	-0.04 [-0.23; 0.15]	0.68	113	-0.14 [-0.32; 0.05]	0.15	113	-0.09 [-0.28; 0.09]	0.32	111	-0.09 [-0.28; 0.10]	0.33	111	-0.06 [-0.25; 0.13]	0.53
	mod1	114	-0.05 [-0.24; 0.14]	0.61	114	-0.03 [-0.22; 0.15]	0.74	113	-0.12 [-0.30; 0.06]	0.18	113	-0.08 [-0.26; 0.10]	0.39	111	-0.08 [-0.27; 0.10]	0.38	111	-0.06 [-0.25; 0.13]	0.56
Ratio of apolipoprotein B to apolipoprotein A1	unadj.	114	-0.07 [-0.26; 0.11]	0.43	114	-0.03 [-0.22; 0.16]	0.76	113	-0.15 [-0.33; 0.04]	0.12	113	-0.11 [-0.29; 0.08]	0.26	111	-0.08 [-0.27; 0.11]	0.38	111	-0.10 [-0.28; 0.09]	0.32
	mod1	114	-0.08 [-0.26; 0.11]	0.41	114	-0.03 [-0.22; 0.15]	0.73	113	-0.16 [-0.33; 0.02]	0.082	113	-0.11 [-0.29; 0.07]	0.22	111	-0.09 [-0.28; 0.10]	0.36	111	-0.10 [-0.29; 0.09]	0.32
**Fatty acids**																			
Total fatty acids	unadj.	114	-0.11 [-0.30; 0.08]	0.25	114	-0.07 [-0.26; 0.11]	0.45	113	-0.19 [-0.37; -0.00]	0.045	113	-0.14 [-0.32; 0.05]	0.14	111	-0.10 [-0.29; 0.09]	0.30	111	-0.08 [-0.27; 0.11]	0.38
	mod1	114	-0.12 [-0.30; 0.07]	0.22	114	-0.08 [-0.27; 0.10]	0.38	113	-0.21 [-0.38; -0.03]	0.021	113	-0.15 [-0.33; 0.03]	0.096	111	-0.11 [-0.30; 0.08]	0.26	111	-0.08 [-0.27; 0.11]	0.38
Degree of unsaturation	unadj.	114	-0.07 [-0.26; 0.11]	0.44	114	-0.05 [-0.24; 0.14]	0.60	113	-0.16 [-0.34; 0.03]	0.098	113	-0.10 [-0.29; 0.09]	0.29	111	-0.14 [-0.33; 0.04]	0.13	111	-0.12 [-0.31; 0.07]	0.22
	mod1	114	-0.07 [-0.26; 0.12]	0.46	114	-0.04 [-0.23; 0.15]	0.67	113	-0.15 [-0.32; 0.03]	0.10	113	-0.09 [-0.27; 0.09]	0.32	111	-0.15 [-0.34; 0.04]	0.12	111	-0.13 [-0.32; 0.06]	0.19
Omega-3 fatty acids	unadj.	114	-0.09 [-0.28; 0.09]	0.33	114	-0.05 [-0.24; 0.14]	0.61	113	-0.16 [-0.34; 0.03]	0.095	113	-0.09 [-0.28; 0.10]	0.35	111	-0.16 [-0.35; 0.03]	0.094	111	-0.14 [-0.33; 0.05]	0.15
	mod1	114	-0.10 [-0.29; 0.09]	0.29	114	-0.05 [-0.24; 0.13]	0.58	113	-0.18 [-0.35; -0.00]	0.050	113	-0.10 [-0.29; 0.08]	0.26	111	-0.17 [-0.36; 0.01]	0.069	111	-0.15 [-0.34; 0.04]	0.11
Omega-6 fatty acids	unadj.	114	-0.14 [-0.32; 0.05]	0.14	114	-0.10 [-0.29; 0.09]	0.29	113	-0.21 [-0.40; -0.03]	0.024	113	-0.17 [-0.35; 0.02]	0.079	111	-0.10 [-0.29; 0.09]	0.32	111	-0.08 [-0.27; 0.11]	0.41
	mod1	114	-0.15 [-0.33; 0.04]	0.12	114	-0.11 [-0.29; 0.08]	0.24	113	-0.23 [-0.40; -0.05]	0.011	113	-0.18 [-0.36; 0.00]	0.051	111	-0.10 [-0.29; 0.09]	0.29	111	-0.08 [-0.27; 0.11]	0.43
PUFA	unadj.	114	-0.14 [-0.32; 0.05]	0.15	114	-0.09 [-0.28; 0.09]	0.32	113	-0.21 [-0.39; -0.03]	0.025	113	-0.16 [-0.34; 0.03]	0.093	111	-0.11 [-0.30; 0.08]	0.24	111	-0.09 [-0.28; 0.10]	0.33
	mod1	114	-0.14 [-0.33; 0.04]	0.13	114	-0.10 [-0.29; 0.08]	0.27	113	-0.23 [-0.40; -0.05]	0.011	113	-0.17 [-0.35; 0.01]	0.061	111	-0.12 [-0.31; 0.07]	0.21	111	-0.09 [-0.28; 0.10]	0.33
MUFA	unadj.	114	-0.11 [-0.29; 0.08]	0.26	114	-0.07 [-0.25; 0.12]	0.49	113	-0.19 [-0.37; -0.00]	0.046	113	-0.14 [-0.32; 0.05]	0.14	111	-0.08 [-0.27; 0.11]	0.39	111	-0.08 [-0.27; 0.11]	0.42
	mod1	114	-0.11 [-0.30; 0.07]	0.23	114	-0.08 [-0.26; 0.11]	0.41	113	-0.21 [-0.38; -0.03]	0.020	113	-0.15 [-0.33; 0.02]	0.091	111	-0.09 [-0.28; 0.10]	0.34	111	-0.08 [-0.27; 0.11]	0.42
SFA	unadj.	114	-0.09 [-0.27; 0.10]	0.36	114	-0.06 [-0.24; 0.13]	0.55	113	-0.17 [-0.35; 0.02]	0.079	113	-0.12 [-0.30; 0.07]	0.22	111	-0.10 [-0.29; 0.09]	0.29	111	-0.08 [-0.27; 0.11]	0.40
	mod1	114	-0.09 [-0.28; 0.09]	0.33	114	-0.07 [-0.25; 0.12]	0.48	113	-0.18 [-0.36; -0.01]	0.043	113	-0.13 [-0.31; 0.05]	0.16	111	-0.11 [-0.30; 0.08]	0.26	111	-0.08 [-0.27; 0.11]	0.41
Linoleic acid	unadj.	114	-0.17 [-0.36; 0.01]	0.067	114	-0.12 [-0.30; 0.07]	0.22	113	-0.24 [-0.43; -0.06]	0.0096 *	113	-0.19 [-0.38; -0.01]	0.041	111	-0.10 [-0.29; 0.09]	0.31	111	-0.09 [-0.28; 0.10]	0.35
	mod1	114	-0.18 [-0.37; 0.00]	0.052	114	-0.13 [-0.32; 0.05]	0.16	113	-0.27 [-0.44; -0.09]	0.0027 **	113	-0.21 [-0.39; -0.03]	0.020	111	-0.11 [-0.30; 0.08]	0.26	111	-0.09 [-0.28; 0.10]	0.36
Docosahexaenoic acid	unadj.	114	-0.21 [-0.39; -0.02]	0.027	114	-0.12 [-0.30; 0.07]	0.22	113	-0.31 [-0.49; -0.13]	<0.001 **	113	-0.23 [-0.41; -0.05]	0.015	111	-0.16 [-0.35; 0.02]	0.084	111	-0.17 [-0.36; 0.01]	0.070
	mod1	114	-0.21 [-0.39; -0.03]	0.024	114	-0.12 [-0.31; 0.06]	0.19	113	-0.32 [-0.49; -0.15]	<0.001 **	113	-0.24 [-0.41; -0.06]	0.0087 *	111	-0.17 [-0.36; 0.01]	0.070	111	-0.18 [-0.36; 0.01]	0.068
Omega-3 FA / total FA	unadj.	114	-0.00 [-0.19; 0.18]	0.96	114	0.02 [-0.17; 0.21]	0.82	113	-0.06 [-0.25; 0.13]	0.52	113	-0.00 [-0.19; 0.19]	>0.99	111	-0.17 [-0.36; 0.02]	0.072	111	-0.15 [-0.34; 0.03]	0.11
	mod1	114	-0.00 [-0.19; 0.19]	0.98	114	0.03 [-0.16; 0.22]	0.73	113	-0.06 [-0.24; 0.12]	0.51	113	0.00 [-0.18; 0.19]	>0.99	111	-0.18 [-0.38; 0.01]	0.057	111	-0.18 [-0.37; 0.01]	0.068
Omega-6 FA / total FA	unadj.	114	-0.04 [-0.22; 0.15]	0.70	114	-0.07 [-0.25; 0.12]	0.49	113	0.05 [-0.14; 0.24]	0.60	113	-0.02 [-0.20; 0.17]	0.87	111	0.12 [-0.07; 0.31]	0.22	111	0.12 [-0.07; 0.31]	0.22
	mod1	114	-0.03 [-0.22; 0.16]	0.74	114	-0.07 [-0.26; 0.12]	0.48	113	0.06 [-0.12; 0.24]	0.48	113	-0.00 [-0.19; 0.18]	0.96	111	0.13 [-0.06; 0.32]	0.18	111	0.13 [-0.06; 0.32]	0.17
PUFA / total FA	unadj.	114	-0.05 [-0.24; 0.14]	0.58	114	-0.08 [-0.26; 0.11]	0.43	113	0.03 [-0.16; 0.22]	0.76	113	-0.02 [-0.21; 0.17]	0.82	111	0.05 [-0.14; 0.24]	0.62	111	0.06 [-0.13; 0.25]	0.54
	mod1	114	-0.04 [-0.23; 0.14]	0.64	114	-0.07 [-0.26; 0.12]	0.46	113	0.05 [-0.13; 0.23]	0.60	113	-0.01 [-0.19; 0.18]	0.95	111	0.06 [-0.13; 0.25]	0.56	111	0.07 [-0.12; 0.26]	0.49
MUFA / total FA	unadj.	114	-0.07 [-0.25; 0.12]	0.49	114	-0.02 [-0.21; 0.17]	0.83	113	-0.13 [-0.32; 0.06]	0.17	113	-0.08 [-0.27; 0.11]	0.40	111	-0.04 [-0.23; 0.15]	0.71	111	-0.07 [-0.25; 0.12]	0.50
	mod1	114	-0.08 [-0.27; 0.11]	0.41	114	-0.03 [-0.22; 0.15]	0.73	113	-0.16 [-0.33; 0.02]	0.078	113	-0.10 [-0.28; 0.08]	0.26	111	-0.05 [-0.24; 0.14]	0.59	111	-0.07 [-0.27; 0.12]	0.44
SFA / total FA	unadj.	114	0.22 [0.04; 0.40]	0.018	114	0.17 [-0.01; 0.36]	0.065	113	0.20 [0.02; 0.38]	0.033	113	0.19 [0.01; 0.38]	0.042	111	-0.01 [-0.20; 0.18]	0.88	111	0.02 [-0.17; 0.21]	0.81
	mod1	114	0.23 [0.05; 0.41]	0.013	114	0.19 [0.00; 0.37]	0.044	113	0.22 [0.05; 0.40]	0.012	113	0.21 [0.03; 0.39]	0.020	111	0.00 [-0.19; 0.19]	0.98	111	0.03 [-0.16; 0.22]	0.78
Linoleic acid / total FA	unadj.	114	-0.22 [-0.40; -0.04]	0.018	114	-0.15 [-0.33; 0.04]	0.12	113	-0.23 [-0.41; -0.05]	0.014	113	-0.22 [-0.41; -0.04]	0.017	111	-0.05 [-0.24; 0.14]	0.61	111	-0.09 [-0.27; 0.10]	0.37
	mod1	114	-0.25 [-0.43; -0.06]	0.0094 *	114	-0.18 [-0.37; 0.00]	0.054	113	-0.28 [-0.45; -0.11]	0.0019 **	113	-0.27 [-0.44; -0.09]	0.0039 *	111	-0.07 [-0.27; 0.12]	0.47	111	-0.08 [-0.28; 0.11]	0.40
Docosahexaenoic acid / total FA	unadj.	114	-0.15 [-0.34; 0.03]	0.10	114	-0.08 [-0.27; 0.10]	0.37	113	-0.19 [-0.37; -0.00]	0.048	113	-0.15 [-0.33; 0.04]	0.12	111	-0.09 [-0.28; 0.09]	0.32	111	-0.12 [-0.31; 0.06]	0.19
	mod1	114	-0.16 [-0.34; 0.03]	0.095	114	-0.09 [-0.27; 0.10]	0.35	113	-0.19 [-0.37; -0.02]	0.029	113	-0.15 [-0.33; 0.02]	0.090	111	-0.10 [-0.29; 0.09]	0.28	111	-0.13 [-0.32; 0.06]	0.18
PUFA / MUFA	unadj.	114	0.01 [-0.17; 0.20]	0.89	114	-0.02 [-0.21; 0.16]	0.81	113	0.08 [-0.10; 0.27]	0.38	113	0.02 [-0.16; 0.21]	0.80	111	0.05 [-0.14; 0.24]	0.60	111	0.07 [-0.12; 0.26]	0.47
	mod1	114	0.02 [-0.17; 0.21]	0.81	114	-0.01 [-0.20; 0.17]	0.88	113	0.11 [-0.07; 0.28]	0.24	113	0.04 [-0.14; 0.23]	0.63	111	0.06 [-0.13; 0.25]	0.50	111	0.08 [-0.11; 0.27]	0.41
Omega-6 FA / omega-3 FA	unadj.	114	0.02 [-0.16; 0.21]	0.81	114	0.01 [-0.18; 0.19]	0.96	113	0.10 [-0.09; 0.29]	0.30	113	0.04 [-0.15; 0.22]	0.71	111	0.19 [-0.00; 0.37]	0.052	111	0.14 [-0.05; 0.33]	0.14
	mod1	114	0.02 [-0.17; 0.21]	0.84	114	-0.00 [-0.19; 0.18]	0.96	113	0.09 [-0.09; 0.27]	0.31	113	0.03 [-0.15; 0.21]	0.75	111	0.19 [-0.00; 0.37]	0.051	111	0.15 [-0.04; 0.34]	0.11
**Phospholipids**																			
Phosphoglycerides	unadj.	113	-0.11 [-0.30; 0.08]	0.25	113	-0.04 [-0.23; 0.15]	0.67	112	-0.16 [-0.34; 0.03]	0.097	112	-0.13 [-0.32; 0.06]	0.17	110	-0.04 [-0.24; 0.15]	0.64	110	-0.09 [-0.28; 0.10]	0.37
	mod1	113	-0.11 [-0.30; 0.07]	0.24	113	-0.04 [-0.23; 0.14]	0.64	112	-0.17 [-0.34; 0.01]	0.064	112	-0.14 [-0.32; 0.04]	0.13	110	-0.05 [-0.24; 0.14]	0.60	110	-0.09 [-0.28; 0.10]	0.35
Ratio of triglycerides to phosphoglycerides	unadj.	113	0.02 [-0.16; 0.21]	0.81	113	0.10 [-0.09; 0.29]	0.29	112	0.07 [-0.12; 0.26]	0.47	112	0.12 [-0.07; 0.31]	0.21	110	-0.08 [-0.27; 0.11]	0.43	110	-0.10 [-0.29; 0.09]	0.28
	mod1	113	-0.01 [-0.20; 0.19]	0.93	113	0.07 [-0.12; 0.26]	0.48	112	-0.01 [-0.19; 0.18]	0.94	112	0.06 [-0.13; 0.24]	0.54	110	-0.12 [-0.31; 0.08]	0.24	110	-0.13 [-0.32; 0.07]	0.19
Total cholines	unadj.	114	-0.11 [-0.29; 0.08]	0.26	114	-0.08 [-0.26; 0.11]	0.42	113	-0.20 [-0.38; -0.02]	0.033	113	-0.15 [-0.34; 0.03]	0.11	111	-0.10 [-0.29; 0.09]	0.30	111	-0.08 [-0.27; 0.11]	0.39
	mod1	114	-0.11 [-0.29; 0.08]	0.25	114	-0.08 [-0.26; 0.11]	0.40	113	-0.20 [-0.38; -0.03]	0.023	113	-0.15 [-0.33; 0.02]	0.091	111	-0.10 [-0.29; 0.09]	0.29	111	-0.08 [-0.27; 0.11]	0.41
Phosphatidylcholines	unadj.	114	-0.11 [-0.30; 0.07]	0.24	114	-0.07 [-0.26; 0.11]	0.43	113	-0.20 [-0.38; -0.01]	0.036	113	-0.15 [-0.34; 0.04]	0.11	111	-0.10 [-0.29; 0.09]	0.30	111	-0.09 [-0.27; 0.10]	0.37
	mod1	114	-0.12 [-0.30; 0.07]	0.22	114	-0.08 [-0.27; 0.10]	0.39	113	-0.21 [-0.38; -0.03]	0.021	113	-0.16 [-0.34; 0.02]	0.084	111	-0.10 [-0.29; 0.09]	0.28	111	-0.08 [-0.27; 0.11]	0.39
Sphingomyelins	unadj.	114	-0.05 [-0.23; 0.14]	0.62	114	-0.04 [-0.23; 0.15]	0.67	113	-0.14 [-0.33; 0.04]	0.14	113	-0.11 [-0.29; 0.08]	0.27	111	-0.09 [-0.28; 0.09]	0.33	111	-0.06 [-0.25; 0.13]	0.51
	mod1	114	-0.04 [-0.23; 0.14]	0.65	114	-0.04 [-0.22; 0.15]	0.68	113	-0.13 [-0.31; 0.04]	0.14	113	-0.10 [-0.28; 0.08]	0.28	111	-0.09 [-0.28; 0.10]	0.35	111	-0.06 [-0.25; 0.13]	0.54
