# Supplementary material for: Maternal and umbilical cord serum lipids in gestational diabetes predict offspring insulin secretion and resistance at the age of nine years
Source: Metabolomics. 2025 Jun 22;21(4):87. doi: 10.1007/s11306-025-02281-9 (PMC12183131; doi:10.1007/s11306-025-02281-9)
Supplement: Supplementary file 4 — Supplementary Table 6– Associations between serum metabolites and offspring ISOBMI at nine years age [file 11306_2025_2281_MOESM4_ESM.pdf]

Supplementary table 6 – Univariate associations between serum metabolites and offspring ISOBMI at nine years age

Associations are given for regression coefficients (beta) with 95% confidence intervals (CI). P-values below 0.05 are denoted with #. Cord blood lipoprotein lipids were log converted and marked with "*log*" in the table.

	Maternal serum (h30)			Maternal serum (h36)			Cord blood			
**Metabolite**	**n**	**Beta ± 95% CI**	**p-value**	**n**	**Beta ± 95% CI**	**p-value**		**n**	**Beta ± 95% CI**	**p-value**
**Lipids**										
Total cholesterol	122	-0.10 [-0.28; 0.08]	0.29	115	-0.12 [-0.31; 0.06]	0.20		118	-0.08 [-0.26; 0.10]	0.39
Total cholesterol minus HDL-C	122	-0.07 [-0.25; 0.11]	0.42	115	-0.09 [-0.28; 0.09]	0.32	*log*	118	-0.06 [-0.25; 0.12]	0.50
Remnant cholesterol	122	-0.07 [-0.25; 0.11]	0.43	115	-0.10 [-0.28; 0.09]	0.30	*log*	118	-0.03 [-0.22; 0.15]	0.72
VLDL cholesterol	122	-0.05 [-0.23; 0.13]	0.60	115	-0.08 [-0.27; 0.10]	0.38	*log*	118	0.01 [-0.17; 0.20]	0.90
Clinical LDL cholesterol	122	-0.08 [-0.26; 0.10]	0.40	115	-0.09 [-0.27; 0.10]	0.36	*log*	118	-0.10 [-0.28; 0.08]	0.27
LDL cholesterol	122	-0.07 [-0.25; 0.11]	0.42	115	-0.09 [-0.27; 0.10]	0.35	*log*	118	-0.10 [-0.29; 0.08]	0.26
HDL cholesterol	122	-0.09 [-0.27; 0.09]	0.34	115	-0.10 [-0.29; 0.08]	0.28	*log*	118	-0.17 [-0.35; 0.01]	0.062
Total triglycerides	122	-0.02 [-0.20; 0.16]	0.86	115	-0.07 [-0.25; 0.12]	0.48		118	-0.01 [-0.19; 0.18]	0.95
Triglycerides in VLDL	122	-0.01 [-0.19; 0.17]	0.89	115	-0.06 [-0.24; 0.13]	0.54	*log*	118	0.04 [-0.14; 0.23]	0.65
Triglycerides in LDL	122	-0.02 [-0.20; 0.17]	0.86	115	-0.06 [-0.25; 0.13]	0.52	*log*	118	-0.03 [-0.22; 0.15]	0.74
Triglycerides in HDL	122	-0.05 [-0.23; 0.13]	0.62	115	-0.12 [-0.30; 0.07]	0.22	*log*	118	-0.02 [-0.20; 0.17]	0.85
Total phospholipids in lipoprotein particles	122	-0.11 [-0.29; 0.07]	0.21	115	-0.17 [-0.35; 0.02]	0.078		118	-0.08 [-0.27; 0.10]	0.37
Phospholipids in VLDL	122	-0.03 [-0.21; 0.15]	0.76	115	-0.07 [-0.26; 0.11]	0.43	*log*	118	0.02 [-0.16; 0.20]	0.83
Phospholipids in LDL	122	-0.08 [-0.26; 0.10]	0.39	115	-0.09 [-0.28; 0.09]	0.33	*log*	118	-0.08 [-0.26; 0.10]	0.38
Phospholipids in HDL	122	-0.09 [-0.27; 0.09]	0.32	115	-0.12 [-0.31; 0.06]	0.20	*log*	118	-0.14 [-0.32; 0.04]	0.13
Total lipids in lipoprotein particles	122	-0.09 [-0.27; 0.09]	0.35	115	-0.13 [-0.31; 0.06]	0.17		118	-0.07 [-0.25; 0.12]	0.47
Total lipids in VLDL	122	-0.03 [-0.21; 0.15]	0.76	115	-0.07 [-0.26; 0.11]	0.44	*log*	118	0.03 [-0.16; 0.21]	0.78
Total lipids in LDL	122	-0.07 [-0.25; 0.11]	0.43	115	-0.09 [-0.27; 0.10]	0.35	*log*	118	-0.08 [-0.27; 0.10]	0.36
Total lipids in HDL	122	-0.09 [-0.27; 0.09]	0.30	115	-0.12 [-0.31; 0.06]	0.18	*log*	118	-0.15 [-0.33; 0.03]	0.10
Total concentration of lipoprotein particles	122	-0.10 [-0.28; 0.08]	0.29	115	-0.16 [-0.34; 0.03]	0.093		118	-0.11 [-0.29; 0.07]	0.24
Concentration of VLDL particles	122	-0.03 [-0.21; 0.15]	0.71	115	-0.09 [-0.27; 0.10]	0.37	*log*	118	0.00 [-0.18; 0.19]	0.99
Concentration of LDL particles	122	-0.06 [-0.24; 0.12]	0.48	115	-0.08 [-0.27; 0.10]	0.38	*log*	118	-0.06 [-0.24; 0.13]	0.54
Concentration of HDL particles	122	-0.08 [-0.26; 0.10]	0.36	115	-0.14 [-0.32; 0.05]	0.14	*log*	118	-0.13 [-0.31; 0.05]	0.16
Average diameter for VLDL particles	122	-0.02 [-0.20; 0.16]	0.80	115	-0.03 [-0.21; 0.16]	0.77		118	0.07 [-0.12; 0.25]	0.47
Average diameter for LDL particles	122	-0.02 [-0.20; 0.16]	0.85	115	-0.01 [-0.19; 0.18]	0.94		118	-0.05 [-0.23; 0.14]	0.63
Average diameter for HDL particles	122	-0.11 [-0.29; 0.07]	0.23	115	-0.09 [-0.28; 0.09]	0.31		118	-0.13 [-0.31; 0.06]	0.17
**Detailed lipoprotein contents**										
Concentration of XXL-VLDL particles	122	0.01 [-0.17; 0.19]	0.91	115	-0.01 [-0.19; 0.18]	0.94			NA	
Concentration of XL-VLDL particles	122	-0.01 [-0.19; 0.17]	0.93	115	-0.05 [-0.23; 0.14]	0.62			NA	
Concentration of L-VLDL particles	122	-0.01 [-0.19; 0.17]	0.90	115	-0.07 [-0.25; 0.12]	0.47			NA	
Concentration of M-VLDL particles	122	-0.04 [-0.22; 0.14]	0.63	115	-0.09 [-0.28; 0.09]	0.33			NA	
Concentration of S-VLDL particles	122	-0.02 [-0.20; 0.16]	0.79	115	-0.09 [-0.27; 0.10]	0.36	*log*	118	0.02 [-0.16; 0.20]	0.82
Concentration of XS-VLDL particles	122	-0.04 [-0.22; 0.14]	0.64	115	-0.08 [-0.27; 0.11]	0.39	*log*	118	-0.04 [-0.22; 0.15]	0.71
Concentration of IDL particles	122	-0.08 [-0.26; 0.10]	0.39	115	-0.09 [-0.28; 0.09]	0.33	*log*	118	-0.16 [-0.34; 0.02]	0.083
Concentration of L-LDL particles	122	-0.06 [-0.24; 0.12]	0.48	115	-0.08 [-0.27; 0.10]	0.38	*log*	118	-0.04 [-0.22; 0.15]	0.69
Concentration of M-LDL particles	122	-0.06 [-0.24; 0.12]	0.51	115	-0.08 [-0.27; 0.10]	0.39	*log*	118	-0.04 [-0.22; 0.14]	0.68
Concentration of S-LDL particles	122	-0.07 [-0.25; 0.11]	0.46	115	-0.08 [-0.27; 0.10]	0.38	*log*	118	-0.11 [-0.29; 0.08]	0.25
Concentration of XL-HDL particles	122	-0.12 [-0.30; 0.06]	0.18	115	-0.14 [-0.32; 0.05]	0.15	*log*	118	-0.15 [-0.33; 0.03]	0.11
Concentration of L-HDL particles	122	-0.12 [-0.30; 0.06]	0.20	115	-0.14 [-0.32; 0.05]	0.14	*log*	118	-0.20 [-0.38; -0.02]	0.034 #
Concentration of M-HDL particles	122	-0.06 [-0.24; 0.12]	0.52	115	-0.11 [-0.29; 0.08]	0.25	*log*	118	-0.14 [-0.32; 0.04]	0.14
Concentration of S-HDL particles	122	-0.01 [-0.19; 0.17]	0.88	115	-0.08 [-0.27; 0.10]	0.38	*log*	118	-0.07 [-0.26; 0.11]	0.43
Total lipids in XXL-VLDL	122	0.01 [-0.17; 0.19]	0.94	115	-0.00 [-0.19; 0.18]	0.97			NA	
Total lipids in XL-VLDL	122	-0.01 [-0.19; 0.17]	0.91	115	-0.05 [-0.23; 0.14]	0.62			NA	
Total lipids in L-VLDL	122	-0.02 [-0.20; 0.16]	0.84	115	-0.07 [-0.26; 0.12]	0.46			NA	
Total lipids in M-VLDL	122	-0.05 [-0.23; 0.13]	0.61	115	-0.10 [-0.28; 0.09]	0.30			NA	
Total lipids in S-VLDL	122	-0.03 [-0.21; 0.15]	0.71	115	-0.09 [-0.27; 0.10]	0.35	*log*	118	0.02 [-0.16; 0.20]	0.83
Total lipids in XS-VLDL	122	-0.05 [-0.23; 0.13]	0.60	115	-0.08 [-0.27; 0.10]	0.38	*log*	118	-0.01 [-0.20; 0.17]	0.88
Total lipids in IDL	122	-0.09 [-0.27; 0.09]	0.31	115	-0.10 [-0.29; 0.08]	0.26	*log*	118	-0.08 [-0.26; 0.10]	0.39
Total lipids in L-LDL	122	-0.08 [-0.26; 0.10]	0.41	115	-0.09 [-0.28; 0.09]	0.32	*log*	118	-0.08 [-0.26; 0.11]	0.40
Total lipids in M-LDL	122	-0.06 [-0.24; 0.12]	0.50	115	-0.08 [-0.27; 0.10]	0.39	*log*	118	-0.05 [-0.24; 0.13]	0.56
Total lipids in S-LDL	122	-0.07 [-0.25; 0.11]	0.44	115	-0.08 [-0.27; 0.10]	0.39	*log*	118	-0.11 [-0.29; 0.07]	0.23
Total lipids in XL-HDL	122	-0.11 [-0.29; 0.07]	0.21	115	-0.12 [-0.30; 0.07]	0.21	*log*	118	-0.15 [-0.33; 0.03]	0.11
Total lipids in L-HDL	122	-0.11 [-0.29; 0.07]	0.25	115	-0.12 [-0.30; 0.07]	0.20	*log*	118	-0.18 [-0.36; -0.00]	0.047 #
Total lipids in M-HDL	122	-0.04 [-0.22; 0.14]	0.64	115	-0.08 [-0.27; 0.10]	0.39	*log*	118	-0.13 [-0.31; 0.06]	0.18
Total lipids in S-HDL	122	-0.00 [-0.18; 0.18]	>0.99	115	-0.08 [-0.26; 0.11]	0.41	*log*	118	-0.05 [-0.23; 0.13]	0.60
Phospholipids in XXL-VLDL	122	0.01 [-0.17; 0.19]	0.90	115	-0.00 [-0.19; 0.18]	0.97			NA	
Cholesterol in XXL-VLDL	122	0.01 [-0.17; 0.19]	0.95	115	-0.03 [-0.21; 0.16]	0.78			NA	
Triglycerides in XXL-VLDL	122	0.01 [-0.17; 0.19]	0.94	115	0.01 [-0.18; 0.19]	0.96			NA	
Phospholipids in XL-VLDL	122	-0.01 [-0.19; 0.17]	0.91	115	-0.05 [-0.23; 0.14]	0.63			NA	
Cholesterol in XL-VLDL	122	-0.03 [-0.21; 0.15]	0.77	115	-0.06 [-0.25; 0.12]	0.49			NA	
Triglycerides in XL-VLDL	122	-0.00 [-0.18; 0.18]	0.97	115	-0.04 [-0.22; 0.15]	0.69			NA	
Phospholipids in L-VLDL	122	-0.01 [-0.19; 0.17]	0.92	115	-0.06 [-0.25; 0.12]	0.50			NA	
Cholesterol in L-VLDL	122	-0.02 [-0.20; 0.16]	0.79	115	-0.08 [-0.27; 0.11]	0.39			NA	
Triglycerides in L-VLDL	122	-0.02 [-0.20; 0.16]	0.85	115	-0.06 [-0.25; 0.12]	0.51			NA	
Phospholipids in M-VLDL	122	-0.05 [-0.23; 0.13]	0.60	115	-0.09 [-0.28; 0.10]	0.34			NA	
Cholesterol in M-VLDL	122	-0.07 [-0.25; 0.11]	0.44	115	-0.09 [-0.27; 0.10]	0.37			NA	
Triglycerides in M-VLDL	122	-0.02 [-0.20; 0.16]	0.80	115	-0.09 [-0.27; 0.10]	0.35			NA	
Phospholipids in S-VLDL	122	-0.04 [-0.22; 0.14]	0.69	115	-0.09 [-0.27; 0.10]	0.36	*log*	118	0.01 [-0.17; 0.19]	0.92
Cholesterol in S-VLDL	122	-0.04 [-0.22; 0.14]	0.70	115	-0.07 [-0.26; 0.11]	0.43	*log*	118	0.02 [-0.16; 0.20]	0.83
Triglycerides in S-VLDL	122	-0.03 [-0.21; 0.15]	0.77	115	-0.10 [-0.28; 0.09]	0.30	*log*	118	0.03 [-0.16; 0.21]	0.77
Phospholipids in XS-VLDL	122	-0.04 [-0.22; 0.14]	0.69	115	-0.08 [-0.26; 0.11]	0.40	*log*	118	-0.01 [-0.19; 0.17]	0.92
Cholesterol in XS-VLDL	122	-0.07 [-0.25; 0.11]	0.46	115	-0.09 [-0.27; 0.10]	0.36	*log*	118	-0.02 [-0.21; 0.16]	0.79
Triglycerides in XS-VLDL	122	-0.01 [-0.19; 0.17]	0.94	115	-0.07 [-0.26; 0.11]	0.45	*log*	118	0.00 [-0.18; 0.19]	0.97
Phospholipids in IDL	122	-0.10 [-0.28; 0.08]	0.28	115	-0.11 [-0.30; 0.08]	0.24	*log*	118	-0.09 [-0.27; 0.09]	0.32
Cholesterol in IDL	122	-0.10 [-0.28; 0.08]	0.29	115	-0.10 [-0.29; 0.08]	0.27	*log*	118	-0.08 [-0.27; 0.10]	0.38

Metabolite	Maternal serum (h30) n	Beta ± 95% CI	p-value	Maternal serum (h36) n	Beta ± 95% CI	p-value	Cord blood	n	Beta ± 95% CI	p-value
Triglycerides in IDL	122	-0.02 [-0.20; 0.17]	0.87	115	-0.07 [-0.25; 0.12]	0.49	*log*	118	-0.03 [-0.21; 0.16]	0.77
Phospholipids in L-LDL	122	-0.08 [-0.26; 0.10]	0.36	115	-0.10 [-0.28; 0.09]	0.31	*log*	118	-0.09 [-0.27; 0.09]	0.33
Cholesterol in L-LDL	122	-0.08 [-0.26; 0.10]	0.40	115	-0.09 [-0.28; 0.09]	0.33	*log*	118	-0.08 [-0.26; 0.11]	0.40
Triglycerides in L-LDL	122	-0.01 [-0.19; 0.17]	0.88	115	-0.06 [-0.25; 0.13]	0.53	*log*	118	-0.07 [-0.26; 0.11]	0.42
Phospholipids in M-LDL	122	-0.07 [-0.25; 0.11]	0.46	115	-0.09 [-0.27; 0.10]	0.36	*log*	118	-0.07 [-0.25; 0.11]	0.46
Cholesterol in M-LDL	122	-0.06 [-0.24; 0.12]	0.49	115	-0.08 [-0.27; 0.11]	0.39	*log*	118	-0.05 [-0.23; 0.13]	0.59
Triglycerides in M-LDL	122	-0.02 [-0.20; 0.16]	0.86	115	-0.06 [-0.25; 0.12]	0.51	*log*	118	-0.06 [-0.24; 0.13]	0.55
Phospholipids in S-LDL	122	-0.07 [-0.25; 0.11]	0.42	115	-0.08 [-0.27; 0.10]	0.39	*log*	118	-0.13 [-0.32; 0.05]	0.15
Cholesterol in S-LDL	122	-0.07 [-0.25; 0.11]	0.44	115	-0.08 [-0.27; 0.10]	0.39	*log*	118	-0.11 [-0.29; 0.07]	0.23
Triglycerides in S-LDL	122	-0.02 [-0.21; 0.16]	0.79	115	-0.06 [-0.25; 0.12]	0.50	*log*	118	-0.08 [-0.27; 0.10]	0.37
Phospholipids in XL-HDL	122	-0.11 [-0.29; 0.07]	0.22	115	-0.12 [-0.30; 0.07]	0.21	*log*	118	-0.15 [-0.33; 0.03]	0.100
Cholesterol in XL-HDL	122	-0.11 [-0.29; 0.07]	0.21	115	-0.11 [-0.29; 0.08]	0.26	*log*	118	-0.15 [-0.33; 0.03]	0.11
Triglycerides in XL-HDL	122	-0.08 [-0.26; 0.10]	0.40	115	-0.12 [-0.30; 0.07]	0.22	*log*	118	-0.07 [-0.25; 0.11]	0.46
Phospholipids in L-HDL	122	-0.10 [-0.28; 0.08]	0.26	115	-0.12 [-0.30; 0.07]	0.21	*log*	118	-0.18 [-0.36; 0.00]	0.056
Cholesterol in L-HDL	122	-0.10 [-0.28; 0.08]	0.26	115	-0.11 [-0.29; 0.08]	0.25	*log*	118	-0.20 [-0.38; -0.01]	0.034 #
Triglycerides in L-HDL	122	-0.11 [-0.29; 0.07]	0.25	115	-0.16 [-0.35; 0.02]	0.078	*log*	118	-0.08 [-0.26; 0.11]	0.40
Phospholipids in M-HDL	122	-0.04 [-0.22; 0.14]	0.67	115	-0.08 [-0.26; 0.11]	0.41	*log*	118	-0.11 [-0.30; 0.07]	0.23
Cholesterol in M-HDL	122	-0.04 [-0.22; 0.14]	0.67	115	-0.06 [-0.25; 0.12]	0.50	*log*	118	-0.15 [-0.33; 0.03]	0.10
Triglycerides in M-HDL	122	-0.03 [-0.21; 0.15]	0.73	115	-0.11 [-0.30; 0.07]	0.23	*log*	118	0.00 [-0.18; 0.18]	>0.99
Phospholipids in S-HDL	122	-0.01 [-0.19; 0.17]	0.88	115	-0.08 [-0.27; 0.10]	0.38	*log*	118	-0.05 [-0.24; 0.13]	0.57
Cholesterol in S-HDL	122	0.02 [-0.16; 0.20]	0.83	115	-0.04 [-0.22; 0.15]	0.69	*log*	118	-0.06 [-0.24; 0.13]	0.55
Triglycerides in S-HDL	122	-0.00 [-0.18; 0.18]	0.98	115	-0.07 [-0.26; 0.11]	0.45	*log*	118	0.02 [-0.16; 0.21]	0.81
**Apolipoproteins**										
Apolipoprotein B	122	-0.06 [-0.24; 0.12]	0.48	115	-0.09 [-0.27; 0.10]	0.36		118	-0.06 [-0.25; 0.12]	0.50
Apolipoprotein A1	122	-0.10 [-0.28; 0.08]	0.28	115	-0.13 [-0.32; 0.05]	0.16		118	-0.12 [-0.30; 0.06]	0.19
Ratio of apolipoprotein B to apolipoprotein A1	122	-0.00 [-0.18; 0.18]	0.97	115	0.01 [-0.18; 0.19]	0.95		118	-0.03 [-0.21; 0.16]	0.77
**Fatty acids**										
Total fatty acids	122	-0.06 [-0.24; 0.12]	0.48	113	-0.06 [-0.25; 0.13]	0.54		118	-0.07 [-0.25; 0.11]	0.44
Degree of unsaturation	122	-0.14 [-0.32; 0.04]	0.12	113	-0.16 [-0.35; 0.03]	0.090		118	-0.10 [-0.29; 0.08]	0.27
Omega-3 fatty acids	122	-0.06 [-0.24; 0.12]	0.50	113	-0.08 [-0.27; 0.10]	0.38		118	-0.06 [-0.25; 0.12]	0.49
Omega-6 fatty acids	122	-0.12 [-0.30; 0.06]	0.17	113	-0.11 [-0.30; 0.07]	0.23		118	-0.10 [-0.28; 0.09]	0.30
PUFA	122	-0.12 [-0.30; 0.06]	0.17	113	-0.12 [-0.30; 0.07]	0.21		118	-0.09 [-0.28; 0.09]	0.31
MUFA	122	-0.02 [-0.20; 0.16]	0.86	113	-0.02 [-0.21; 0.17]	0.85		118	-0.05 [-0.24; 0.13]	0.58
SFA	122	-0.05 [-0.23; 0.13]	0.57	113	-0.05 [-0.23; 0.14]	0.63		118	-0.07 [-0.25; 0.12]	0.48
Linoleic acid	122	-0.13 [-0.31; 0.05]	0.16	113	-0.12 [-0.31; 0.06]	0.19		118	-0.10 [-0.28; 0.08]	0.27
Docosahexaenoic acid	122	-0.05 [-0.23; 0.13]	0.57	113	-0.08 [-0.27; 0.11]	0.40		118	-0.13 [-0.31; 0.05]	0.16
Omega-3 FA / total FA	122	-0.05 [-0.23; 0.13]	0.56	113	-0.09 [-0.28; 0.10]	0.34		118	-0.04 [-0.23; 0.14]	0.65
Omega-6 FA / total FA	122	-0.03 [-0.21; 0.15]	0.75	113	-0.03 [-0.22; 0.16]	0.75		118	-0.01 [-0.20; 0.17]	0.89
PUFA / total FA	122	-0.05 [-0.23; 0.13]	0.61	113	-0.06 [-0.25; 0.13]	0.52		118	-0.05 [-0.23; 0.14]	0.62
MUFA / total FA	122	0.07 [-0.11; 0.25]	0.42	113	0.05 [-0.14; 0.24]	0.58		118	0.02 [-0.16; 0.21]	0.80
SFA / total FA	122	-0.02 [-0.20; 0.16]	0.86	113	0.04 [-0.15; 0.23]	0.66		118	0.04 [-0.15; 0.22]	0.69
Linoleic acid / total FA	122	-0.09 [-0.27; 0.09]	0.35	113	-0.09 [-0.28; 0.09]	0.32		118	-0.09 [-0.28; 0.09]	0.32
Docosahexaenoic acid / total FA	122	-0.01 [-0.19; 0.17]	0.92	113	-0.05 [-0.23; 0.14]	0.63		118	-0.06 [-0.24; 0.13]	0.54
PUFA / MUFA	122	-0.06 [-0.24; 0.12]	0.53	113	-0.06 [-0.25; 0.13]	0.53		118	-0.03 [-0.22; 0.15]	0.72
Omega-6 FA / omega-3 FA	122	0.09 [-0.09; 0.27]	0.32	115	0.08 [-0.11; 0.27]	0.40		118	0.10 [-0.08; 0.28]	0.27
**Phospholipids**										
Phosphoglycerides	121	-0.05 [-0.23; 0.13]	0.59	112	-0.10 [-0.29; 0.09]	0.31		117	-0.08 [-0.26; 0.11]	0.41
Ratio of triglycerides to phosphoglycerides	121	0.04 [-0.14; 0.22]	0.69	112	0.02 [-0.17; 0.21]	0.83		117	0.15 [-0.03; 0.34]	0.096
Total cholines	122	-0.10 [-0.28; 0.08]	0.26	112	-0.10 [-0.29; 0.09]	0.28		118	-0.10 [-0.28; 0.09]	0.30
Phosphatidylcholines	122	-0.11 [-0.29; 0.07]	0.24	112	-0.11 [-0.30; 0.07]	0.23		118	-0.09 [-0.28; 0.09]	0.31
Sphingomyelins	122	-0.10 [-0.28; 0.08]	0.28	112	-0.08 [-0.27; 0.10]	0.38		118	-0.07 [-0.26; 0.11]	0.43
