# Supplementary material for: Maternal and umbilical cord serum lipids in gestational diabetes predict offspring insulin secretion and resistance at the age of nine years
Source: Metabolomics. 2025 Jun 22;21(4):87. doi: 10.1007/s11306-025-02281-9 (PMC12183131; doi:10.1007/s11306-025-02281-9)
Supplement: Supplementary file 5 — Supplementary Table 7– Associations between serum metabolites and outcome variables with significant treatment group interactions [file 11306_2025_2281_MOESM5_ESM.pdf]

Supplementary table 7 – Associations between serum metabolites and outcome variables with singificant treatment group interactions

Metabolite	Model	Group	HOMA2-IR$_{CP}$				HOMA2-IR$_{Ins}$			
			n	*beta [95%CI]*	*p-value*	*interaction p-value*	*n*	*beta [95%CI]*	*p-value*	*interaction p-value*
Linoleic acid	Unadj.	Combined	114	-0.17 [-0.36; 0.01]	0.067		114	-0.12 [-0.30; 0.07]	0.22	
		Insulin	59	-0.48 [-0.75; -0.21]	<0.001 *	0.0035 *	59	-0.36 [-0.63; -0.08]	0.011	0.021
		Metformin	55	0.07 [-0.17; 0.31]	0.58		55	0.08 [-0.17; 0.32]	0.53	
Linoleic acid	Adj.	Combined	114	-0.18 [-0.37; 0.00]	0.052		114	-0.13 [-0.32; 0.05]	0.16	
		Insulin	59	-0.50 [-0.77; -0.23]	<0.001 *	0.0024 *	59	-0.38 [-0.65; -0.11]	0.0066	0.016
		Metformin	55	0.07 [-0.17; 0.31]	0.56		55	0.07 [-0.17; 0.32]	0.57	
Linoleic acid / total FA	Unadj.	Combined	114	-0.22 [-0.40; -0.04]	0.018		114	-0.15 [-0.33; 0.04]	0.12	
		Insulin	59	-0.45 [-0.67; -0.23]	<1e-04 *	<0.001 *	59	-0.43 [-0.65; -0.21]	<0.001 *	<1e-04 *
		Metformin	55	0.18 [-0.11; 0.47]	0.21		55	0.34 [0.06; 0.63]	0.019	
Linoleic acid / total FA	Adj.	Combined	114	-0.25 [-0.43; -0.06]	0.0094		114	-0.18 [-0.37; 0.00]	0.054	
		Insulin	59	-0.46 [-0.68; -0.24]	<1e-04 *	0.0013 *	59	-0.44 [-0.65; -0.22]	<1e-04 *	<1e-04 *
		Metformin	55	0.16 [-0.14; 0.46]	0.30		55	0.31 [0.01; 0.61]	0.042	

Metabolite	Model	Group	AUC$_{CP/Gluc}$				AUC$_{Ins/Gluc}$			
			n	*beta [95%CI]*	*p-value*	*interaction p-value*	*n*	*beta [95%CI]*	*p-value*	*interaction p-value*
Linoleic acid / total FA	Unadj.	Combined	113	-0.23 [-0.41; -0.05]	0.014		113	-0.22 [-0.41; -0.04]	0.017	
		Insulin	58	-0.48 [-0.70; -0.26]	<1e-04 *	<0.001 *	58	-0.49 [-0.71; -0.28]	<1e-04 *	<1e-04 *
		Metformin	55	0.20 [-0.08; 0.49]	0.16		55	0.25 [-0.03; 0.54]	0.083	
Linoleic acid / total FA	Adj.	Combined	113	-0.28 [-0.45; -0.11]	0.0019 *		113	-0.27 [-0.44; -0.09]	0.0039	
		Insulin	58	-0.50 [-0.70; -0.29]	<1e-05 *	<0.001 *	58	-0.51 [-0.71; -0.30]	<1e-05 *	<0.001 *
		Metformin	55	0.14 [-0.14; 0.42]	0.33		55	0.20 [-0.08; 0.49]	0.16	

Associations are given for regression coefficients (beta) with 95% confidence intervals (CI). Adjusted model (Adj.) is adjusted for maternal pre-pregnancy BMI. P-values below 0.0038 are denoted with *.
