# Supplementary material for: Maternal and umbilical cord serum lipids in gestational diabetes predict offspring insulin secretion and resistance at the age of nine years
Source: Metabolomics. 2025 Jun 22;21(4):87. doi: 10.1007/s11306-025-02281-9 (PMC12183131; doi:10.1007/s11306-025-02281-9)
Supplement: Supplementary file 6 — Supplementary Figure 1– Associations between outcome variables in the children at the age of nine years [file 11306_2025_2281_MOESM6_ESM.pdf]

SUPPLEMENTARY FIGURES

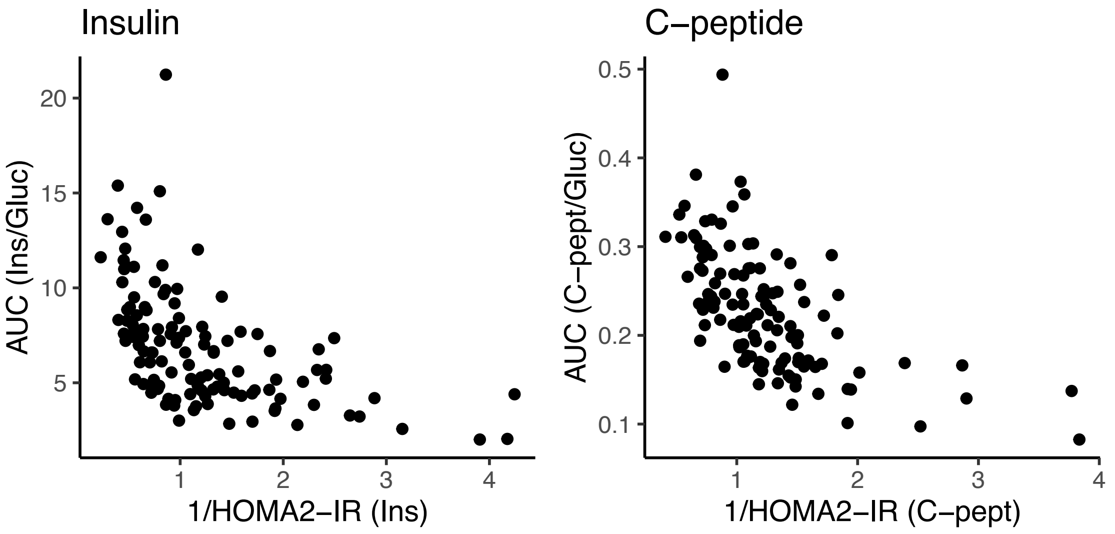

Supplementary figure 1 – Associations between outcome variables in the children at the age of nine years

Units for $AUC_{Ins/Gluc}$ is mU/mol and $AUC_{C\text{-}pept/Gluc}$ nmol/mol.
